# Ethnobotanical study on medicinal plant knowledge among three ethnic groups in peri-urban areas of south-central Ethiopia

**DOI:** 10.1186/s13002-023-00629-w

**Published:** 2023-11-23

**Authors:** Sintayehu Tamene, Mesele Negash, Fortunatus Bulabo Makonda, Linley Chiwona-Karltun, Kefyalew Sahle Kibret

**Affiliations:** 1 Wondo Genet College of Forestry and Natural Resources, Hawassa University, PO Box 05, Hawassa, Ethiopia; 2 College of Forestry, Wildlife, and Tourism, Sokoine University of Agriculture, Morogoro, Tanzania; 3 Department of Urban and Rural Development, Swedish University of Agricultural Sciences, Uppsala, Sweden

**Keywords:** Ethiopia, Ethnobotanical knowledge, Peri-urban, South-central Ethiopia, Medicinal plants

## Abstract

**Background:**

Documenting traditional knowledge on plant use among ethnic groups has enabled researchers to obtain a better understanding of how indigenous flora is seen and used in daily life. Their therapeutic applications will also encourage future conservation and phytochemical research, potentially leading to the development of novel drugs. However, past ethnobotanical studies conducted in Ethiopia mainly focused on rural areas, and limited coverage to document the ethnobotanical knowledge at the rural‒urban interface. Therefore, this study was conducted to document and analyze traditional ethnobotanical knowledge on medicinal plants among three selected ethnic groups in peri-urban areas of south-central Ethiopia. In addition, we attempted to investigate the range of cultural similarity and disparity between the studied ethnic groups in relation to traditional medicinal plants and diseases treated.

**Methods:**

Data were collected using semistructured questionnaires and in-depth interviews of 189 key informants, floristic species inventories, and field observations. Several cultural importance indices and Rahman’s similarity indices were applied to analyze the relevance of medicinal plants and cultural similarity among the ethnic groups.

**Results:**

A total of 189 therapeutic plants representing 159 genera and 69 families were identified and documented across the three studied ethnic groups. Of these, the Sidama, Gedeo, and Oromo ethnic groups reported 28, 34, and 38%, respectively. Most medicinal plants were represented by herbs (36%), followed by shrubs (31%), trees (27%), and herbaceous climbers (7%). Rahman's similarity index (RSI) revealed considerable ethnobotanical knowledge variation among ethnic groups. Oromo and Sidama showed the highest disparity (63.8%), followed by Gedeo and Oromo (63.2%). Of the total collected therapeutic plants, 78 most important medicinal plants were selected for the cultural importance analysis, which revealed that *Croton macrostachyus* Hochst. ex Delile scored the highest point in the Gedeo and Oromo ethnic groups and *Zingiber officinale* Roscoe in the Sidama ethnic group. Whereas *Cinnamomum verum* J.Presl*, Psidium guajava* L., and *Melia azedarach L.* are the least.

**Conclusion:**

The present study revealed the presence of cultural differences in medicinal plant knowledge practices and therapeutic plant use among the studied ethnic groups in rural–urban interface areas of south-central Ethiopia. The diverse healing potential of plants would support future pharmacological investigations, emphasizing the need for adequate documentation of indigenous knowledge and versatile flora to prevent their further loss.

**Supplementary Information:**

The online version contains supplementary material available at 10.1186/s13002-023-00629-w.

## Introduction

Traditional knowledge developed over time is influenced by elements of ancestral inheritance, intercultural connection, and interaction with the natural environment [1]. These facts explain the reasons for the variations of traditional knowledge among cultures, locations and ethnic groups [2, 3]. Studies conducted in various regions of the world [3] described the existence of cultural variety in plant use knowledge and treatment systems. Thus, comparative studies in traditional knowledge and plant use culture among ethnic groups have enabled researchers to better understand how indigenous flora is seen and used in daily life [4, 5]. Similarly, traditional medicinal plant knowledge has long been applied in Ethiopia [6], and most oral information has been passed down from generation to generation via professional healers, knowledgeable elders and/or regular people [7]. It is believed that over 80% of Ethiopians still rely on traditional medicine [8], and approximately 95% of traditional medicine preparations in the country are mentioned to be of plant origin [9]. Despite the important role of traditional medicine and medicinal plants in primary health care, little work has been done in the country to properly document and promote the associated knowledge [10] and only a few of the country's diverse cultures and languages have been considered. In addition, more surveys are needed in various sections of the nation, encompassing a broader range of sociocultural groups, with the goal of obtaining unique knowledge and cultural variations [11].

Moreover, in previous studies by Emery and Hurley [12] and Wubetu et al. [13], traditional medicinal plant knowledge in urban and peri-urban settings has been marginalized and not well addressed. Similarly, scientific evidence on the documentation of such medicinal plant species and knowledge linked to them in Ethiopian peri-urban contexts is sparse [14]. In addition, studies in various parts of Ethiopia [15, 16] show that the medicinal plants available in the study areas are increasingly threatened and the accompanying knowledge held by elders has received less attention, putting them on the verge of extinction. Therefore, exploring and documenting the knowledge of diverse ethnic groups on the use of medicinal plants would fill the gap in indigenous knowledge on medicinal plants and provide baseline information for future conservation efforts. Thus, the objective of this study was (1) to document and analyze ethnomedicinal plant resources among the three selected ethnic groups, namely, the peoples of Sidama, Oromo, and Gedeo, regarding the use of plants for the treatment of various human ailments residing in selected districts of the Sidama, Oromia, and Southern Ethiopia administrative regions, respectively; (2) to evaluate ethnobotanical knowledge diversity between the ethnic groups in connection to traditional medicine; and (3) to evaluate the cultural importance of medicinal plant species among ethnic groups. We hypothesized that ethnobotanical knowledge and medicinal plants used vary among the ethnic groups in peri-urban areas of south-central Ethiopia.

## Materials and methods

### Study areas and ethnographic information

**Fig. 1:**
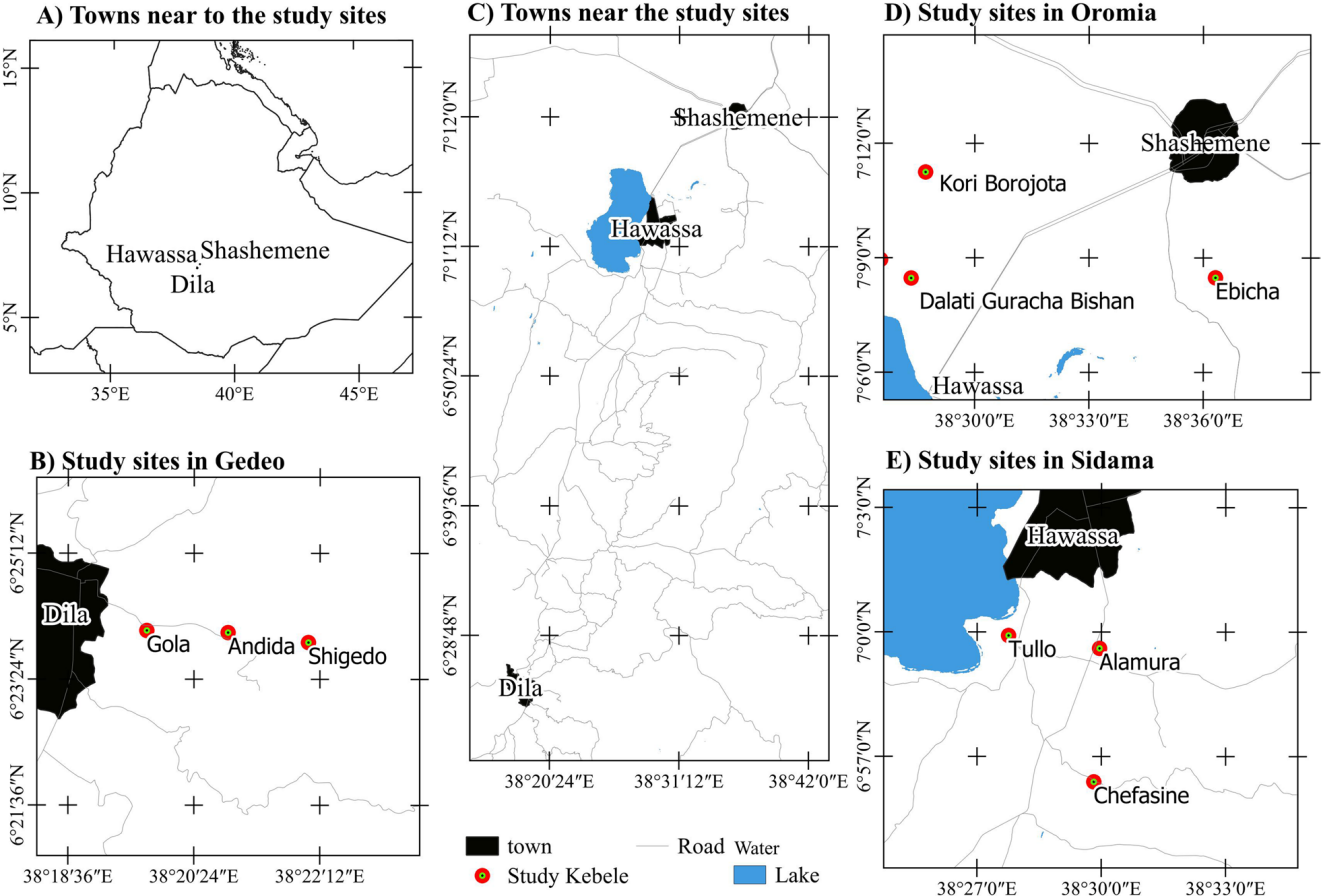
Map of the study areas Gedeo, Oromia, and Sidama

This research was carried out in three peri-urban areas in south-central Ethiopia. Nine peri-urban kebeles (lowest administrative units; three from each study site) were purposely selected at different distances from the peri-urban administrative parts of Hawassa, Shashemene, and Dilla (Fig. 1). These areas were chosen because the ethnic groups living in the districts have coexisted and interacted for many years in specific regions of south-central Ethiopia. Additionally, they are geographically close to the town, have similar urbanization pressures, and are facing aggressive degradation of natural resources owing to urbanization [17–20].

All of the studied ethnic groups speak the Cushitic language. According to Tadesse et al. [21], Sidama National Regional State is the primary coffee-producing region in Ethiopia and covers 73,030 hectares. The region is generally fertile, with varying climates ranging from warm to hot in flat lands, to warm to cold in highlands. The majority of the population in the area relies on subsistence farming, with Coffee (*Coffea arabica* L.) and Enset (*Ensete ventricosum* (Welw.) Cheesman) being the main crops produced [22]. The Sidama people are neighbors of the Oromo, Wolaita, and Gedeo sociocultural groups. The Oromo people belong to the Cushitic-speaking group of Eastern Africa. The majority of the population is engaged in agriculture, with crop production accounting for roughly half of the total national production [23]. The main cash crops produced in the Oromia region are coffee and khat (a mild stimulant). People in pastoral and agro-pastoral areas rely on livestock and its products for subsistence and as a source of income in addition to agricultural production [23], and neighbors for the Sidama, Wolaita, and Gedeo sociocultural groups. The Gedeo people are an ethnic group in southern Ethiopia who live in the Gedeo Zone which stretches along the main highway from Addis Ababa to Moyale for about 360 km [24]. The geological zone is 70% Woina Dega (mid-altitude), ranging from 1800 to 3200 m a.s.l.; 28% Dega (high land, 2400 to 3200 m a.s.l.); and 2% Kola (lowlands, 500 to 1800 m a.s.l.). In addition to Sidama, the Gedeo Zone is the leading producer of coffee, enset, and other agroforestry products [19, 25], and a neighbor of the Oromia, Wolaita, and Sidama sociocultural groups.

Hawassa city is situated 273 km south of Addis Ababa [26], at 06° 27'–07° 40' N and 37° 21'–39° 11' E. The borders of Hawassa city are defined by Lake Hawassa to the west, Oromia National Regional State to the north, Wondo Genet and Malga districts to the east, and Shebedino and Gorge districts to the south. The city's elevation ranges from 1697 to 1708 m a.s.l. [27]. Hawassa city had 15,720 hectares of land within its administrative boundary, while only 6,465 hectares (24.4%) were demarcated within the municipal boundary and planned as urban land, while the rest is rural land [26]. Administratively, the city is organized into three tiers of administration: City administration, 8 sub-cities, and 32 kebeles (lowest administrative units) (Hawassa City Administration annual unpublished report, 2019). Among the sub-cities, Hawella-Tulla and its 12 kebeles are categorized as rural and rural–urban interface areas, which is where the current study was conducted (Fig. 1). Residents of the city are ethnically and religiously diverse. The majority of indigenous people living in the area are Sidama (49%), followed by Amhara, Welaita, Oromo, and Gurage CSA [24]. More than half of the people in the research area practice protestant religions (59.7%), followed by Ethiopian Orthodox Christianity, Islam, and Catholicism. According to the CSA [24] population forecasts, the projected population for 2022 was 555,480, of whom 277,032 were males and 278,448 were females.

Shashemene district is located at 7° 04′50″ to 7° 22′45″ N and 38° 23′00″ to 38° 48′00″ E. Which is 250 km to the south of Ethiopia's capital city, Addis Ababa, and 25 km north of Hawassa, the capital city of Sidama National Regional State [28]. The Hawassa city borders it to the south, to the west by Seraro, to the north by Arsi Negele, and to the east by East Arsi Zone. Its elevation ranges from 1500 to 2300 m a.s.l. [28]. The district had 767.9km^2^ area with 458.3/Km^2^ population density [24]. The district rural and rural–urban interface area was assembled into 28 kebeles (lowest administrative units), in which the current study was conducted (Fig. 1). Residents of the area are ethnically and religiously diverse. The Oromo ethnic group makes up the majority of the indigenous inhabitants in the district (74.11%), followed by the Amhara, Welaita, Kembata, and Gurage CSA [24]. The majority of the inhabitants were Muslims, with 69.38% of the population, followed by Ethiopian Orthodox Christianity, Protestantism, and Catholicism. According to the CSA [24] population forecasts, the projected population for 2022 was 351,898, of whom 174,711 were males and 177,187 were females in the district.

Dilla district is located in southern Ethiopia, 359 km from the capital city, Addis Ababa [29]. With an altitude range of 1350–2550 m a.s.l. It is situated at 6°15′05" to 6°26′35 N and 38°15′55" to 38°24′02"E. The district had 122.3 km^2^ area with 1,047/Km^2^ population density [24]. In the district rural and rural–urban interface areas was assembled into 19 kebeles (lowest administrative units), where the current study was carried out (Fig. 1). The district residents are heterogeneous, both in ethnicity and in religion. The majority of indigenous people inhabiting the area belong to the Gedeo ethnic group (49%), followed by the Amhara, Welaita, Oromo, and the Gurage CSA [24]. The majority of the people were Protestants, accounting for 83.13% of the population, followed by Ethiopian Orthodox Christianity, Catholicism, and Islam. According to the CSA [24] population forecasts, the projected population for 2023 is expected to reach 128,050 of whom 64,276 were males and 63,774 were females in the district.

### Informant selection

To ensure a detailed representation of local knowledge and plant use, key informants were selected based on gender, age, religion, experience, and level of education. A total of 189 respondents (63 from each study site), aged 35 and above, were selected using purposive and snowball sampling techniques, following the approaches of Tongco [30], and Espinosa and Bieski [31]. These traditional healers were from the Sidama, Oromo, and Gedeo ethnic groups, and their communication languages were Sidamu affo, Afaan Oromo, and Gedeoffa, which is a native language to the respective research location. Following an approach of Alexiades [32] before the interviews, discussions were held with the informants with the help of local elders, and development agents to inform the aim of the study. This helped to get the respondents' confidence in providing truthful information without suspicion. Thus, informed consent was obtained from each informant who participated in the study.

### Ethnobotanical data collection

Sequential exploratory design and mixed method approach was applied [33]. An ethnobotanical survey was carried out from February to October 2022. Following Gollin et al*.* [34], a group discussion comprising traditional healers, local elders, and development agents was held at all research locations to test the relevance and acceptability of the questionnaires used for the interview.

Following this, key informant interviews and medicinal plant inventories were conducted to collect all relevant ethnobotanical and floristic species data in very close interaction with informants [35, 36]. Interviews were addressed in two sessions. The first session included information regarding the socio-demographic characteristics of the informants. Secondly, information related to local names of medicinal plants used, ailments treated, habitat of the species, sources of medicinal plants (wild or cultivated), abundance, parts used, condition of plant parts used (fresh or dried), methods of remedy preparation, source of knowledge, method of indigenous knowledge transfer, and traditional conservation practices were collected following the methods from [35, 36]. All semistructured interviews were carried out separately, allowing for further discussion with the informant and practical identification of traditionally used medicinal plants in the natural environment.

Floristic voucher specimens were collected with the help of traditional healers and development professionals. Specimens were identified in the field and later confirmed at the National Herbarium of Addis Ababa University using taxonomic keys and flora [37–41]. The species name and its categorization of the genus and family were further validated using the Plants of the World Online (https://powo.science.kew.org.) websites. Finally, the plants were dried, pressed, mounted on a herbarium sheet, and placed at the Herbarium of Wondo Genet College of Forestry and Natural Resources, Hawassa University, and the National Herbarium of Addis Ababa University.

### Ethnobotanical data analysis

The ethnobotanyR package, Version 0.1.8, 2022 was used for both qualitative and quantitative data analysis [42]. Following [4, 41–46], several quantitative ethnobotanical tools such as Rahman’s similarity index (RSI), use report (UR), frequency of citation (FC), number of uses (NU), cultural importance (CI), relative frequency of citation (RFC), cultural value (CV), relative importance (RI) were determined. These indices were used to evaluate ethnobotanical knowledge and shared cultural elements among the ethnic groups studied and to determine the cultural importance of the most commonly utilized and relevant medicinal plant species.

### Analysis of overlap for cited plant species

The ethnomedicinal plant species of the three ethnic groups studied were compared (Gedeo, Oromo, and Sidama). Data is represented as a Venn diagram using a package for creating highly customized Venn and Euler diagrams in R software. (https://bmcbioinformatics.biomedcentral.com/articles/10.1186/1471-2105-12-35#citeas).

### Rahman similarity index (RSI)

The index was used to investigate the cultural similarities and differences between ethnic groups of different areas by calculating unique and shared medicinal plant species used to treat the same medicinal usage [48].

$${\text{RSI}} = \frac{d}{a + b + c - d}$$

where “*a*” is the number of species unique in area *A*, “*b*” is the number of species unique in area *B*, “*c*” is the number of common species in both areas *A* and *B*, and “*d*” is the number of common species used for similar ailments in both areas *A* and *B*. While *a* & *b* ≠ 0 and *c* & *d* ≥ 0.

## 7The use report (UR)

According to Kufer et al*.* [43], the most fundamental ethnobotany calculation and common way to examine the cultural relevance of plants is to count the total number of usage reports (UR) for each species.

$${\text{UR}}_{s} = \sum\limits_{{u = u_{i} }}^{{^{u} {\text{NC}}}} {\sum\limits_{{i = i_{1} }}^{{^{i} N}} {{\text{UR}}_{{{\text{ui}}}} } }$$

It is the sum of all uses in each use category (from u1 to uNC) and the number of informants who mention each use-category NC for the species.

### Number of uses (NU)

This index gives the number of uses (NU) for each species in the data and is the total of all categories for which a species is helpful [43].

$${\text{NU}}_{s} = \sum\limits_{{u = u_{1} }}^{{^{u} NC}} {}$$

NC denotes the number of use categories, whereas NUs denote the total number of categories for which a species is helpful.

### Cultural importance index (CI)

According to Kufer et al*.* [4], this index was used to determine the most commonly used and culturally significant medicinal plant species based on the overall proportion of informants who mention the use of each species though taking into account the spread of usage (number of informants) for each species and the range of uses.

$${\text{CI}}_{s} = \sum\limits_{{u = u_{i} }}^{{^{u} {\text{NC}}}} {\sum\limits_{{i = i_{1} }}^{{^{i} N}} {{\text{UR}}_{ui/N} } }$$

The index's theoretical maximum value is the total number of various use categories (NC), which would be attained if all informants mentioned the use of the species in all of the use categories considered in the survey.

#### Relative frequency of citation (RFC)

This index has been used to investigate the significance or importance of each species found in the surrounding area. It was calculated by dividing the number of informants who confirmed the frequency of citation (FC) by the total number of informants (N) who participated in the survey [4, 46].

$${\text{RFC}}_{s} = \frac{{{\text{FC}}_{s} }}{N} = \frac{{\sum\nolimits_{{i = i_{1} }}^{{^{t} N}} {{\text{UR}}_{i} } }}{N}$$

Using the same terminology, the numerator can be interpreted as the sum of the UR of all informants questioned for the species without considering the use category. As a result, this index theoretically ranges from zero when no one mentions the plant's utility to one in the uncommon event that all informants remark on the species' utility.

#### Cultural value index (CV)

[44] Created this index by multiplying the relative values of the species' frequency of citation (FC/N), number of uses (NU/NC), and number of use reports (UR/N).

$$CV_{s} = \left[ {\frac{{NU_{s} }}{NC}} \right] \times \left[ {\frac{{FC_{s} }}{N}} \right] \times \left[ {\sum\limits_{{u = u_{i} }}^{{^{u} {\text{NC}}}} {\sum\limits_{{i = i_{1} }}^{{^{i} N}} {{\text{UR}}_{{\text{ui/N}}} } } } \right]$$

The theoretical maximum value would be obtained if all of the components were at their maximum; in the improbable event that all of the informants mentioned the use of the species (FCs = N) in all of the usage categories evaluated in the survey (NUs = NC). The first two variables would be one, and the third variable would be the total number of possible use categories (NC). As a result, this index ranges from zero to NC.

#### Relative importance index (RI)

This index only considers the use categories [4]. Where RFCs (max) is the relative frequency of citation over the maximum, calculated by dividing FCs by the maximum value in all of the survey's species, and RNUs (max) is the relative number of use categories over the maximum, calculated by dividing the number of uses of the species by the maximum value in all of the survey's species.

$${\text{RI}}_{s} = \frac{{{\text{RFC}}_{s(\max )} + {\text{RNU}}_{s(\max )} }}{2}$$

The RI index theoretically ranges from zero when no one acknowledges any usage of the plant to one when the plant was cited as useful the most frequently and in the highest number of use categories.

## Results

### Sociodemographic features of informants

The demographic features have been documented based on the information provided by traditional healers. Out of 189 key informants, 56 were females and 133 were males; 63 from each ethnic group were interviewed. Informants were divided into 4 age groups, and about 70% of those participating in this study were between the ages of 45 and 65. While those under 45 (young) and over 65 (elderly) accounted for 30% of the remaining population, nineteen (19) informants had completed high school; 81 had completed elementary school; and 89 were illiterate. The majority of the interviewees were protestant religious followers (101), 75 were Muslims, and 13 were Orthodox Christians.

### Medicinal plant composition and distributions

**Fig. 2:**
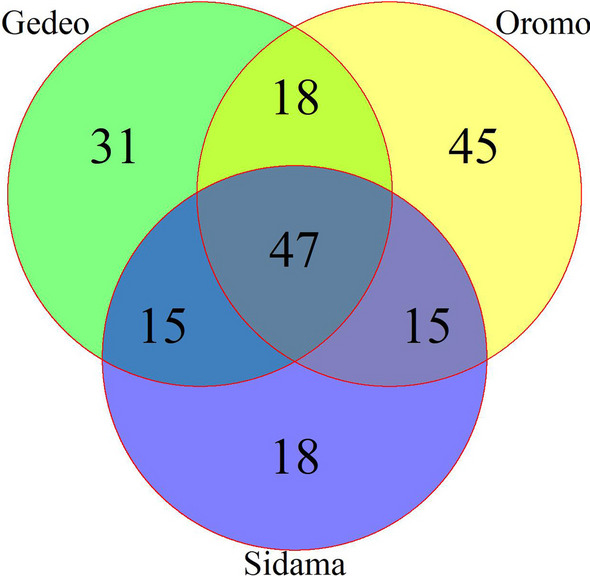
Venn diagram showing the distributions of medicinal plant resources among the studied ethnic groups

A total of 189 medicinal plant species representing 159 genera and 69 families were identified and documented as being utilized in traditional human ethnomedicine across the three ethnic groups studied (Additional file 1: Table S1). Among these, 95 plant species were reported by the Sidama ethnic group, 111 by the Gedeo, and 125 by the Oromo ethnic groups to treat 60, 65, and 65 different ailments, respectively (Fig. 2). Families Fabaceae, Asteraceae, Poaceae, and Lamiaceae were the most commonly used, represented by 16, 13, 12, and 11 species, respectively. Solanaceae was represented by 9 species, Rutaceae and Cucurbitaceae each represented by 7 and 6 species, and Malvaceae shared 5 (Additional file 1: Table S1). The results of the life form analysis showed that herbs and shrubs constituted the highest proportion (36% and 31%, respectively) of the identified species, followed by trees (27%) and herbaceous climbers (7%). Of the identified medicinal plant species, 71% were harvested from wild areas, 20% from home gardens, and 9% from both wild and home gardens (Additional file 1: Table S1).

All three ethnic groups’ ethnobotanical data (Gedeo, Oromo, and Sidama) were compared and illustrated in the form of a Venn diagram (Fig. 2). Analysis of the distribution of plant resources revealed that 25% of the plants studied overlapped among the three ethnic groups. Compared with other pairs, the Gedeo and Oromo ethnic groups showed more similarity in species distribution (9.5%), followed by Gedeo-Sidama and Oromo-Sidama (7.94% each) (Fig. 2). The Oromo ethnic group used 24% of medicinal plants distinctively, followed by the Gedeo ethnic group (16.4%).

### Ethnobotanical knowledge distribution between ethnic groups

In Fig. 2, we have seen overlaps in species utilization among ethnic groups. To elucidate and differentiate cultural relationships or species similarity and disparity in terms of ailment treatment between the studied ethnic groups, a new ethnobiological tool, the Rahman similarity index (RSI), was used and revealed a high disparity in ethnobotanical knowledge application between the studied ethnic groups (Table 1). A total of 1,055 uses were reported for the identified medicinal plant species. Of these, 336, 366, and 353 were reported from Gedeo, Oromo, and Sidama, respectively (Additional file 1: Table S1). Since some of the species had more than one use, the richness of usage was greater than the richness of species.

**Table 1:** Level of cultural similarity and disparity in ethnobotanical knowledge applications between the studied ethnic groups (Gedeo, Oromo, and Sidama)

Site 1	Site 2	Unique species at site 1	Unique species at site 2	Common species in both sites	Shared species treating similar ailments in both sites	Rahman’s similarity index (%)
Gedeo	Oromo	46	60	65	46	36.8
Gedeo	Sidama	49	33	62	41	39.8
Oromo	Sidama	63	33	62	42	36.2

### Cultural importance and comparisons of medicinal plants

From the collected 189 local flora, the most important 78 medicinal plant species, which were claimed by three or more informants as a remedy, were evaluated and revealed a great variation in preference and extent of utilization of plant species among ethnic groups (Additional file 1: Table S5). We have presented the first top 10 medicinal plant species based on the three essential values, namely, the frequency of citations (FC), usage reports (UR), and number of uses (NU) (Table 2). Accordingly, considerable disparities in species importance were observed across the Gedeo, Oromo, and Sidama ethnic groups. The different values of the CI, RFC, RI, and CV indices reflect the ranking of different plant species depending on each index. The recorded variation in the species ranking of the various indices indicates the relevance of plant species in the respective ethnic groups.

**Table 2:** Evaluation of useful plants of Sidama, Oromo, and Gedeo using four quantitative indices

Ethnic group	Species	Basic values			Indices				Rankings			
		FC	UR	NU	CI	RFC	RI	CV	CI	RFC	CV	RI
Sidama	*Zingiber officinale* Roscoe	12	46	13	0.730	0.190	0.786	0.030	1	6	2	2
	*Croton macrostachyus* Hochst. ex Delile	17	36	15	0.571	0.270	1.000	0.039	2	1	1	1
	*Gymnanthemum amygdalinum* (Delile) Sch.Bip	13	25	6	0.397	0.206	0.582	0.008	3	5	7	7
	*Allium sativum* L	8	23	9	0.365	0.127	0.535	0.007	4	13	8	9
	*Ruta chalepensis* L	14	21	8	0.333	0.222	0.678	0.010	5	3	5	5
	*Cucumis prophetarum* L	14	20	9	0.317	0.222	0.712	0.011	6	4	3	3
	*Ekebergia capensis* Sparrm	15	20	8	0.317	0.238	0.708	0.010	7	2	4	4
	*Eucalyptus globulus* Labill	9	20	11	0.317	0.143	0.631	0.008	8	11	6	6
	*Rotheca myricoides* (Hochst.) Steane & Mabb	11	16	7	0.254	0.175	0.557	0.005	9	8	10	8
	*Melia azedarach* L	8	15	9	0.238	0.127	0.535	0.005	10	14	9	10
Oromo	*Croton macrostachyus* Hochst. ex Delile	21	48	22	0.762	0.333	1.000	0.086	1	1	1	1
	*Aloe macrocarpa* Tod	16	38	14	0.603	0.254	0.699	0.033	2	3	3	3
	*Gymnanthemum amygdalinum* (Delile) Sch.Bip	21	34	15	0.540	0.333	0.841	0.042	3	2	2	2
	*Moringa stenopetala* (Baker f.) Cufod	13	25	14	0.397	0.206	0.628	0.018	4	4	5	5
	*Olea europaea subsp. cuspidata* (Wall. & G.Don) Cif	11	25	18	0.397	0.175	0.671	0.019	5	6	4	4
	*Ruta chalepensis* L	9	19	13	0.302	0.143	0.510	0.009	6	9	6	7
	*Eucalyptus globulus* Labill	9	16	5	0.254	0.143	0.328	0.003	7	10	14	13
	*Melia azedarach* L	11	16	11	0.254	0.175	0.512	0.008	8	7	7	6
	*Zingiber officinale* Roscoe	8	16	11	0.254	0.127	0.440	0.005	9	12	8	10
	*Calpurnia aurea* (Aiton) Benth	11	14	9	0.222	0.175	0.466	0.005	10	8	9	9
Gedeo	*Croton macrostachyus* Hochst. ex Delile	28	80	21	1.270	0.444	1.000	0.182	1	1	1	1
	*Albizia gummifera* (J.F.Gmel.) C.A.Sm	20	47	20	0.746	0.317	0.833	0.073	2	2	2	2
	*Calpurnia aurea* (Aiton) Benth	19	31	13	0.492	0.302	0.649	0.030	3	3	3	4
	*Ruta chalepensis* L	15	28	17	0.444	0.238	0.673	0.028	4	5	4	3
	*Afrocarpus falcatus* (Thunb.) C.N.Page	16	26	10	0.413	0.254	0.524	0.016	5	4	5	5
	*Solanecio gigas* (Vatke) C.Jeffrey	13	20	10	0.371	0.206	0.470	0.010	6	6	6	6
	*Celtis africana* Burm.f	10	18	11	0.286	0.159	0.440	0.008	7	8	7	7
	*Gymnanthemum amygdalinum* (Delile) Sch.Bip	11	17	8	0.270	0.175	0.387	0.006	8	7	8	8
	*Cymbopogon citratus* (DC.) Stapf	5	15	9	0.238	0.079	0.304	0.003	9	21	11	13
	*Ocimum gratissimum* L	7	15	6	0.238	0.111	0.268	0.002	10	12	17	18

## Discussion

### Medicinal plant resources of the studied ethnic groups

The present study provides the first quantitative analysis of 189 plant species utilized in traditional human ethnomedicine to address 100 ailments in peri-urban areas of south-central Ethiopia and is groundbreaking since it is the first quantitative ethnomedicinal research effort undertaken on extensively used plants in traditional medicine in the study districts. A higher number of medicinal plants have been recorded in Shashemene and Dilla peri-urban districts than in Hawassa per-urban areas. This could be due to the higher plant species diversity in the former districts due to the presence of natural forests, agroforestry practices, and plantations as well as higher plant knowledge acquisition by traditional healers in the studied areas. Moreover, the existence of interactions with agroforestry and natural resource institutions of learning, such as Wondo Genet College of Forestry and Natural Resources, Hawassa University, is attributed to the higher presence of plant resources. Several studies conducted in Ethiopia and abroad have reported varying quantities of therapeutic plants identified as a remedy for different human ailments. For instance, Chekole [7], Regassa et al. [49], Tefera and Kim [16], Tadeyos and Wendawek [50], and Mekuria and Abduro [51] compiled 51, 25, 70, 62, and 43 medicinal plant species, respectively, in their ethnobotanical investigations in different parts of Ethiopia. Ishtiaq et al. [52], Tugume et al. [53], Wiryono et al. [54], and Al-Robai et al. [55] documented 10, 27, 9, and 21 therapeutic plants, respectively, in Indonesia, Uganda, Pakistan, and Saudi Arabia.

Herbs were reported more frequently than other growth forms. Presumably, due to their widespread availability, the presence of market accessibility, and their greater therapeutic efficacy. Several studies reported similar results [56–58]. Fabaceae, Asteraceae, Poaceae, Lamiaceae, Solanaceae, Rutaceae, Cucurbitaceae, and Euphorbiaceae had the most dominantly utilized plant families among the three ethnic groups, suggesting that these plant families continue to provide a wide range of medical benefits to the local community. Several scientists have also developed hypotheses about specific plant families as part of their ethnobotanical study. For instance, Amjad et al*.* [59] demonstrated that the dominance of Asteraceae and Lamiaceae species in treating ailments is most likely due to the presence of secondary metabolites. In addition, [60–62] suggested that the dominance of these families in disease treatment might be due to their aromatic properties and abundance of essential oils. Furthermore, the findings of [41] indicate that these families were dominant in the flora of Ethiopia and Eritrea. Similar findings have also been observed elsewhere in the tropics [47, 49] and [54–59]. Furthermore, the discussions with informants also revealed that the local people have been using these medicinal plants for many generations and have acquired these skills through repeated, long-term practices.

### Cross-cultural analysis of ethnobotanical knowledge

This finding strongly confirmed our hypothesis, which revealed a big difference in the knowledge of medicinal plants utilized among ethnic groups and ethnobotanical knowledge richness (Additional file 2: Table S2, Additional file 3: Table S3, Additional file 4: Table S4). The disparity was comparable in shared medicinal plant knowledge between the Oromo and Sidama ethnic groups (63.8%), and the Gedeo and Oromo ethnic groups (63.2%; Table 1). The difference in the use of reports may suggest social barriers [48]. For instance, the majority of Oromo healers were Muslims, and their language is different from that of the Sidama and Gedeo ethnic groups. This might hamper the sharing of ethnobotanical knowledge across ethnic groups, particularly the secrecy of healers [46, 60]. In comparison, the Oromo ethnic group solely reported a higher number of unique medicinal plant species than Sidama and Gedeo (Table 1). This might be due to the wider market access, intermarriage, mobile pastoralists, and proximity to natural and plantation forests, which make the knowledge broader than that of other ethnic groups. For instance [3, 46, 61, 62] conducted similar cross-cultural ethnobotanical knowledge comparisons in various countries and concluded that ethnicity and cultural practices have shaped traditional ethnobotanical knowledge among local inhabitants and might result in isolated knowledge of plant utilization [68–70].

For instance, *Achyranthes aspera* L*.* is a common medicinal plant species among the three studied ethnic groups. Although it has been reported to heal a variety of illnesses. It is claimed to heal respiratory organ illnesses between the Gedeo and Oromo ethnic groups (Additional file 2: Table S2), which is a differed use report from other pairs, and to cure spiritual complications between Gedeo and Sidama ethnic groups (Additional file 3: Table S3), and stomachache between Oromo and Sidama (Additional file 4: Table S4), respectively. Similarly, *Aloe macrocarpa* Tod. is a shared medicinal plant between the Gedeo and Oromo and Oromo and Sidama ethnic groups. However, it was described as a curative plant against gonorrhea between the Gedeo and Oromo ethnic groups, as well as malaria between the Oromo and Sidama ethnic groups (Additional file 2: Table S2, Additional file 4: Table S4). Again, *Croton macrostachyus* Hochst. ex Delile was a common medicinal plant across all the studied ethnic groups. However, in each pair, it was reported to cure three district ailments. These findings might indicate disparities in knowledge about the medicinal use of various plants among the ethnic groups studied (Additional file 2: Table S2, Additional file 3: Table S3, Additional file 4: Table S4).

The other interesting finding in this study is that there were cultural similarities between ethnic groups (Table 1). As shown in Fig. 1, the study site of the Dilla peri-urban area is geographically far from the Shashemene and Hawassa study areas. However, this study revealed that the Gedeo and Sidama ethnic groups shared greater ethnobotanical knowledge, which was 39.8% higher than the other pairs (Table 1). This demonstrated that these ethnic groups used the same medicinal plants to treat the same ailments and were more culturally related than any other comparable pairs (Additional file 2: Table S2). For instance, both ethnic groups used *Ajuga integrifolia* Buch.-Ham. ex D.Don, *Albizia gummifera* (J.F.Gmel.) C.A.Sm., *Allium sativum* L., *Artemisia abyssinica* Sch.Bip. ex A.Rich., *Calpurnia aurea* (Aiton) Benth., *Carica papaya* L., *Catha edulis* (Vahl) Forssk. ex Endl., *Cinnamomum verum* J. Presl, and *Croton macrostachyus* Hochst. ex Delile against stomachache, dizziness, typhoid, spiritual complications, jaundice, and malaria. This similarity in plant use might be due to a common body of information about sickness, the wide distribution of species in the area, the historical stratifications of the studied ethnic groups, as well as similar sociocultural adaptations and interactions between people and their environments [3, 65, 66]. According to [72], similarities in how various ethnic groups use the same plants may be explained by the fact that some of them have had social connections with others. In addition, religious and linguistic cohesions may promote the transmission of knowledge on medicinal plant usage and illness treatment similarities between the two ethnic groups [73].

### Comparing different indices of ethnomedicine in healing human ailments

Scholars believe that the high cultural importance value indicates that medicinal plants are widely used and highlights a high level of agreement in the survey culture about the species [74]. In this study, the overall analysis of the above-captioned indices revealed the presence of a high level of utilization of medicinal plants among the three ethnic groups studied. In the Sidama ethnic group, *Zingiber officinale* Roscoe scored the highest cultural importance value (0.73), followed by *Croton macrostachyus* Hochst. ex Delile (0.571) and *Gymnanthemum amygdalinum* (Delile) Sch.Bip. (0.397), which claimed to treat various ailments (Table 2). In this ethnic group, because of its use of diversity, *Croton macrostachyus* Hochst. ex Delile ranked at the top by the RFC (0.27), RI (1), and CV (0.039) indices. However, according to Tardío and Pardo-De-Santayana [4], *Zingiber officinale* Roscoe was considered a culturally more significant medicinal plant than *Croton macrostachyus* Hochst. ex Delile. With the same approach, *Croton macrostachyus* Hochst. ex Delile scored a high cultural significance value (0.762) and ranked first by all indices used, followed by *Aloe macrocarpa* Tod. (0.603) and *Gymnanthemum amygdalinum* (Delile) Sch.Bip. (0.54) in the Oromo ethnic group (Table 2). Interestingly, *Croton macrostachyus* Hochst. ex Delile again scored the highest cultural importance value (1.27) in the Gedeo ethnic group than Sidama and Oromo, followed by *Albizia gummifera* (J.F.Gmel.) C.A.Sm. (0.746) and *Calpurnia aurea* (Aiton) Benth. (0.492) (Table 2). Thus, the Gedeo ethnic group, followed by the Oromo, had stronger cultural practices to identify the medicinal potentials of the species (*Croton macrostachyus* Hochst. ex Delile) than the Sidama ethnic group.

It was also true that plants with higher use reports (UR) always had higher utilization levels [75]. Our findings are similar to previous findings by [55] and [76]. They conducted an ethnobotanical survey in different parts of Bangladesh and Saudi Arabia and found that plants with high use reports have high use values. In this study, *Albizia gummifera* (J.F.Gmel.) C.A.Sm. is a shared plant species among ethnic groups, and ranked 12^th^ in the Sidama ethnic group with 13 use reports. Whereas it ranked 18^th^ in the Oromia ethnic group with 10 use reports, and interestingly, it ranked second in having culturally relevant plants to cure various illnesses in the Gedeo ethnic group with 47 use reports (Additional file 5: Table S5). Moreover, *Gymnanthemum amygdalinum* (Delile) Sch. Bip., is again a species shared by all studied ethnic groups, ranked 3^rd^ in the Sidama and Oromo ethnic groups with 25 and 34 use reports as culturally significant medicinal plant species, respectively, whereas 8^th^ in the Gedeo ethnic group with 17 use reports (Table 2). This shows that, when compared among the ethnic groups, *Gymnanthemum amygdalinum* (Delile) Sch. Bip. is found to be a more culturally significant medicinal plant for the Oromo and Sidama ethnic groups.

In addition, medicinal plant species with high RFC and RI values should be recommended for pharmacological and phytochemical studies, as they are widely used and expected to have therapeutic properties [45, 57, 72, 73]. This emphasizes the importance of understanding the potential therapeutic properties of plants for various ethnic groups. In our study, RFC ranged in ascending order from *Cinnamomum verum* J.Presl*, Moringa stenopetala* (Baker f.) Cufod., and *Ricinus communis* L., (0.048 each) to *Croton macrostachyus* Hochst. ex Delile (0.27) in the Sidama ethnic group. Whereas, *Psidium guajava* L. (0.048) to *Croton macrostachyus* Hochst. ex Delile (0.333), *and Lagenaria siceraria* (Molina) Standl., *Melia azedarach* L., *Nigella sativa* L., *Ocimum lamiifolium* Hochst. ex Benth, *Phytolacca dodecandra* L'Hér., and *Zingiber officinale* Roscoe (0.048 each) to *Croton macrostachyus* Hochst. ex Delile (0.444), in the Oromo and Gedeo ethnic group, respectively (Additional file 5: Table S5). Moreover, *Aloe macrocarpa* Tod., *Albizia gummifera* (J.F.Gmel.) C.A.Sm., *Calpurnia aurea* (Aiton) Benth., *Croton macrostachyus* Hochst. ex Delile, *Cucumis prophetarum* L., *Ekebergia capensis Sparrm., Gymnanthemum amygdalinum* (Delile) Sch. Bip., *Afrocarpus falcatus* (Thunb.) C.N.Page, and *Ruta chalepensis* L. had scored the highest RFC and RI values across the studied ethnic groups, thus indicating their strong local medicinal role (Table 2) and highlighting their potential for pharmacological study in the future [78]. Furthermore, [70, 72] suggested that plants with lower RFC and RI scores may be less essential, but their low values may indicate that local people are unfamiliar with their wider utilization, potentially leading to knowledge extinction (Additional file 5: Table S5).

### Public health and the marketability of medicinal plants in the study areas and beyond

The development of new medications and therapies for public health is greatly influenced by ethnobotanical knowledge studies [80, 81]. Because several ethnobotanical investigations could lead to the identification of new medicinal plant sources or the elaboration of the mechanisms behind traditional remedies [79]. For instance, such studies have resulted in the development of important drugs such as reserpine from *Rauvolfia serpentina* (L.) Benth. ex Kur plant species to treat hypertension, podophyllotoxin from *Podophyllum peltatum* L. to treat cancer, and bromelain from *Ananas comosus* (L.) Merr. to treat cancer [79]. According to several studies conducted elsewhere, medicinal plants and spices have recently been produced as natural, efficient antibacterial agents against a wide range of harmful microbes [81]. *Allium sativum* L., *Croton macrostachyus* Hochst. ex Delile, and *Zingiber officinale* Roscoe have been identified as potential plant sources for managing antibacterial, antifungal, and antiviral properties. They are potentially cost-effective in disease management and the problem of drug resistance [79–83].

The present study revealed that plants with high RI and RFC values have promising potential against different pathogens and play a great role in maintaining general public health in the studied areas and beyond. *Afrocarpus falcatus* (Thunb.) C.N.Page, *Aloe macrocarpa* Tod., *Albizia gummifera* (J.F.Gmel.) C.A.Sm., *Calpurnia aurea* (Aiton) Benth., *Croton macrostachyus* Hochst. ex Delile, *Cucumis prophetarum* L., *Ekebergia capensis* Sparrm., *Gymnanthemum amygdalinum* (Delile) Sch. Bip., and *Ruta chalepensis* L. are the most cited medicinal plant species against communicable and non-communicable diseases in the communities we studied (Additional file 1: Table S1). Besides in vitro, investigations of some of the aforementioned medicinal plant species were reported and found to be most effective against various disease-causing pathogens in various parts of Ethiopia [83, 85], and further research is recommended on their pharmacological contents.

During our key informants’ survey and market observation, we realized that income to traditional healers from patient treatment and the sale of medicinal plants was insignificant. Lack of awareness, cheap pricing, and less market access of the traditional medicine are likely influence the income. 80% of the collected medicinal plant resources did not have market access and were limited in dissemination. This would imply that most medicinal plants are only collected from the wild for remedy preparations only when needed. The same findings were reported in elsewhere in Ethiopia [86, 87]. While 20% of the medicinal plants were marketable, for example, *Aframomum corrorima* (A.Braun) P.C.M.Jansen*, Allium cepa* L., *Allium sativum* L., *Aloe macrocarpa* Tod., *Aloe vera* (L.) Burm.f., *Artemisia absinthium* L., *Artemisia abyssinica* Sch.Bip. ex Oliv. & Hiern*, Calpurnia aurea* (Aiton) Benth., *Capsicum frutescens* L., *Carica papaya* L., *Coriandrum sativum* L., *Catha edulis* (Vahl) Forssk. ex Endl., *Cinnamomum verum* J.Presl*, Citrus* × *aurantiifolia* (Christm.) Swingle*, Citrus limon* (L.) Osbeck*, Coffea arabica* L., *Cucurbita pepo* L., *Cymbopogon citratus* (DC.) Stapf, *Echinops kebericho, Eucalyptus globulus* Labill., *Hagenia abyssinica, Indigofera arrecta* Hochst. ex A.Rich., *Kalanchoe densiflora* Rolfe*, Kalanchoe petitiana* A.Rich., *Lactuca inermis* Forssk., *Linum usitatissimum* L., *Lepidium sativum* L., *Mentha spicata* L., *Moringa stenopetala* (Baker f.) Cufod., *Nicotiana tabacum* L., *Nigella sativa* L., *Olea europaea subsp. cuspidata* (Wall. & G.Don) Cif., *Ruta chalepensis* L., *Rhamnus prinoides* L'Hér., *Taverniera abyssinica* A.Rich., *Triticum turgidum subsp. dicoccum* (Schrank ex Schübl.) Thell., *Vicia lens* (L.) Coss. & Germ., *Withania somnifera,* and *Zingiber officinale* Roscoe. Although an in-depth valuation of traditional medicinal plant marketability in the respective research sites was outside the scope of this study, some healers appealed the importance of traditional medicinal plant marketability in the study areas. In overall, the present study could also be used as baseline for a future detailed investigation of the market potential and value chain of medicinal plant resources in the study regions and beyond.

## Conclusion

In this study, 189 medicinal plant species were identified and documented as being utilized in traditional human ethnomedicine across the three ethnic groups studied. This revealed people in the study districts have plentiful traditional ethnobotanical knowledge that has been passed down through generations and have a diverse range of medicinally important plant species capable of treating a wide range of human illnesses. Notably, *Aframomum corrorima* (A. Braun) P.C.M. Jansen*, Afrocarpus falcatus* (Thunb.) C.N.Page, *Aloe macrocarpa* Tod., *Albizia gummifera* (J.F.Gmel.) C.A. Sm, *Cucumis prophetarum* L., *Croton macrostachyus* Hochst. ex Delile*, Ekebergia capensis Sparrm*., *Erythrina abyssinica* Lam*.*, *Gymnanthemum amygdalinum* (Delile) Sch. Bip*.*, *Moringa stenopetala* (Baker f.) Cufod*.*, *Ocimum lamiifolium* Hochst. ex Benth*., Ruta chalepensis* L., *Terminalia brownii* Fresen., *Zingiber officinale* Roscoe, and *Ziziphus spina-christi* (L.) Willd. has been claimed to treat a variety of ailments between ethnic groups. Even though peri-urban areas are rich in medicinal plant diversity, efforts to conserve plants and associated indigenous knowledge are extremely limited. The efforts of some traditional practitioners to cultivate medicinal plants in home gardens necessitate continued government support to promote overall conservation strategies for medicinal plants in the area. It is also recommended that a traditional healers' association be formed as soon as possible to help traditional healers by providing professional support, as such groups can contribute to the conservation of diverse local floras.

### Supplementary Information

**Additional file 1**. List of medicinal plants used to treat human ailments in the study areas.

**Additional file 2**. Rahman similarity index between Gedeo and Oromo ethnic groups.

**Additional file 3**. Rahman similarity index between Gedeo and Sidama ethnic groups.

**Additional file 4**. Rahman similarity index between Oromo and Sidama ethnic groups.

**Additional file 5**. Evaluation of useful plants of the three studied ethnic groups, using four quantitative indices.

## Data Availability

All data generated or analyzed in this study are included in this manuscript, and its supplementary information files are attached as Additional file 1, Additional file 2, Additional file 3, Additional file 4, and Additional file 5.

## Abbreviations

CSA: Central statistical agency of Ethiopia
CV: Cultural value
CI: Cultural importance
DTAPA: Dilla town administration population affairs
FC: Frequency of citation
NU: Number of uses
RSI: Rahman’s similarity index
RFC: Relative frequency of citations
RI: Relative importance
UR: Use report
