# Supplementary material for: Ethnobotanical study on medicinal plant knowledge among three ethnic groups in peri-urban areas of south-central Ethiopia
Source: J Ethnobiol Ethnomed. 2023 Nov 23;19:55. doi: 10.1186/s13002-023-00629-w (PMC10668360; doi:10.1186/s13002-023-00629-w)
Supplement: Supplementary file 1 — Additional file 1. List of medicinal plants used to treat human ailments in the study areas. [file 13002_2023_629_MOESM1_ESM.docx]

Table 1. Medicinal plants of the three ethnic groups (Sidama (S), Gedeo (G), and Oromo (O)) used to treat human ailments with their family and vernacular names, habits, plant parts used, ailments treated in South-central Ethiopia

| Scientific name | Family | Local name | Growth form | Parts used | Ethnic group | Used against | Voucher number |
| --- | --- | --- | --- | --- | --- | --- | --- |
| *Allium cepa* L. | Amaryllidaceae | Qulubi addi | Herb | Fresh bulb | G | Nasal bleeding, Passive sexual interest, Weight loss | St 2022 (1) |
| *Ananas comosus* (L.) Merr. | Bromeliaceae | Anannase | Herb | Fresh fruit, Fresh fruit bark | G | Skin infection | St 2022 (2) |
| *Oldeania alpina* (K.Schum.) Stapleton | Poaceae | Lemma | Shrub | Ash | G | Wound | St 2022 (3) |
| *Arundo donax* L. | Poaceae | Hophetikka | Shrub | Dry leaf | G | Swellings | St 2022 (4) |
| *Bidens macroptera* (Sch.Bip. ex Chiov.) Mesfin. | Asteraceae | Addeyi | Herb | Fresh root | G | Abnormal menstruation cycle, Amoeba, Diarrhea | St 2022 (5) |
| *Capsicum frutescens* L. | Solanaceae | Mixmixxo | Herb | Fresh fruit | G | Amoeba, Intestinal worms | St 2022 (6) |
| *Celtis africana* Burm.f. | Cannabaceae | Shishu | Tree | Fresh bark, Dry bark, Old root, Young fresh leaf, Young root, Fresh seed | G | Stomachache, Jaundice, Skin infection, Wound, Headache, Diarrhea, Asthma, Intestinal worms, Glandular, Lung infection, Giardia | St 2022 (7) |
| *Clausena anisata* (Willd.) Hook.f. ex Benth. | Rutaceae | Lichee/Limich | Shrub | Fresh leaf | G | Swellings | St 2022 (8) |
| *Clutia lanceolata* Forssk*.* | Peraceae | Kudhure | Shrub | Fresh leaf | G | Ear infection | St 2022 (9) |
| *Colocasia esculenta* (L.) Schott | Araceae | Colcomma | Herb | Dry root, Fresh root | G | Cancer, Toothache | St 2022 (10) |
| *Cymbopogon citratus* (DC.) Stapf | Poaceae | Hancura | Herb | Fresh leaf | G | Abortion, Bath of mother after giving birth, Blood pressure, Cancer, Cholesterol, Kidney infection, Stomachache, Gonorrhea, Vomiting | St 2022 (11) |
| *Dalbergia lactea* Vatke | Fabaceae | Batissa | Shrub | Fresh leaf, Fresh seed | G | Gonorrhea, Amoeba | St 2022 (12) |
| *Drynaria volkensii* Heiron. | Polypodiaceae | Bobile | Epiphytes | Fresh root, Fresh leaf | G | Cancer, Ear infection, Swellings, Nasal bleeding | St 2022 (13) |
| *Embelia schimperi* Vatke | Primulaceae | Honkoko | Tree | Fresh leaf | G | Glandular, Gonorrhea, Jaundice | St 2022 (14) |
| *Euphorbia pulcherrima* Willd. ex Klotzsch | Euphorbiaceae | Qorsa abeba | Shrub | Fresh root | G | Fever | St 2022 (15) |
| *Euphorbia tirucalli* L. | Euphorbiaceae | Qinchibi | Shrub | Fresh latex | G | Cancer | St 2022 (16) |
| *Fagaropsis angolensis* (Engl.) H.M.Gardner | Rutaceae | Sissa | Tree | Dry seed, Fresh leaf | G | Stomachache, Wound, Swellings | St 2022 (17) |
| *Flacourtia indica* (Burm.f.) Merr. | Salicaceae | Hagala | Shrub | Fresh fruit, Fresh root | G | Snake poison, Respiratory organ infection | St 2022 (18) |
| *Hibiscus macranthus* Hochst. ex A. Rich. | Malvaceae | Abeba | Shrub | Fresh leaf | G | Fire accident | St 2022 (19) |
| *Hyparrhenia rufa* (Nees) Stapf | Poaceae | Qoricha bekekko | Herb | Fresh leaf | G | Swellings, Cough, Lung infection | St 2022 (20) |
| *Juniperus procera* Hochst. ex Endl. | Cupressaceae | Honcho | Tree | Dry seed | G | Respiratory organ infection | St 2022 (21) |
| *Kanahia laniflora* (Forssk.) R.Br. | Asclepidaceae | Cigga | Shrub | Fresh leaf | G | Jaundice | St 2022 (22) |
| *Leucas tomentosa* Gürke | Lamiaceae | Balbalato | Herb | Fresh leaf | G | Febrile illness | St 2022 (23) |
| *Rubia cordifolia* L. | Rubiaceae | Dummo | Climber | Fresh leaf | G | Malaria | St 2022 (24) |
| *Sesbania sesban* (L.) Merr. | Fabaceae | Shashatto | Shrub | Fresh leaf | G | Rabies, Snake poison | St 2022 (25) |
| *Sida ovata* Forssk. | Malvaceae | Qirqixxe | Shrub | Fresh leaf | G | Bone cancer | St 2022 (26) |
| *Solanum indicum* L. | Solanaceae | Dimoxxa | Shrub | Fresh leaf | G | Nasal bleeding, Skin infection, Snake poison | St 2022 (27) |
| *Sorghum bicolor* (L.) Moench. | Poaceae | Xinqisha | Herb | Fresh root | G | Febrile illness, Respiratory organ infection | St 2022 (28) |
| *Strychnos spinosa* Lam. | Loganiaceae | Goqqumma | Tree | Fresh leaf, Fresh bark | G | Spiritual, Fire accident, Toothache | St 2022 (29) |
| *Gymnanthemum myrianthum* (Hook.f.) H.Rob. | Asteraceae | Rejii | Shrub | Fresh leaf, Dry root | G | Headache, Respiratory organ infection | St 2022 (30) |
| *Xanthium strumarium.* L. | Asteraceae | Qorsi butika | Herb | Fresh leaf | G | Nerve case | St 2022 (31) |
| *Vachellia oerfota* (Forssk.) Kyal. & Boatwr. | Fabaceae | Ajoo | Shrub | Fresh root, Fresh bark | O | Spiritual, General health | St 2022 (32) |
| *Vachellia seyal* (Delile) P.J.H.Hurter | Fabaceae | Waccu | Tree | Fresh bark | O | Intestinal worms | St 2022 (33) |
| *Vachellia tortilis (*Forssk.) Galasso & Banfi | Fabaceae | Dhadacha | Tree | Fresh bark | O | Malaria, Spiritual | St 2022 (34) |
| *Argemone mexicana* L. | Papaveraceae | Wajota | Herb | Fresh latex, Fresh leaf | O | Cancer, Blood pressure, Wound, Jaundice | St 2022 (35) |
| *Beta vulgaris* L. | Amaranthaceae | Keyisir | Herb | Fresh root, Fresh leaf | O | Anemia, Wound | St 2022 (36) |
| *Calendula officinalis* L. | Asteraceae | Olaati | Herb | Fresh seed | O | Amoeba | St 2022 (37) |
| *Capsella bursa-pastoris* Medik. | Brassicaceae | Bursi | Herb | Fresh root | O | Lung infection, Asthma, Cough | St 2022 (38) |
| *Casimiroa edulis* La Llave | Rutaceae | Kazmiree | Tree | Fresh fruit | O | Gastric diseases | St 2022 (39) |
| *Casuarina equisetifolia* L. | Casuarinaceae | Shawshawee | Tree | Fresh leaf | O | Rabies | St 2022 (40) |
| *Citrus limon* (L.) Osbeck | Rutaceae | Lomme | Tree | Fresh leaf, Fresh fruit | O | Blood pressure, Fever, Stomachache, Common cold, Amoeba | St 2022 (41) |
| *Rotheca myricoides* (Hochst.) Steane & Mabb. | Lamiaceae | Marachissa | Shrub | Fresh bark, Fresh leaf | O | Rabies, Stomachache, Spiritual | St 2022 (42) |
| *Cyathula polycephala* Baker | Amaranthaceae | Hixxicho | Herb | Fresh leaf | O | Febrile illness | St 2022 (43) |
| *Daucus carota* L. | Apiaceae | Karotee | Herb | Fresh root, Fresh fruit | O | Jaundice, Passive sexual interest | St 2022 (44) |
| *Eleusine coracana* (L.) Gaertn. | Poaceae | Dagussa | Herb | Fresh seed | O | Bone injury, Wound | St 2022 (45) |
| *Eragrostis tef* (Zuccagni) Trotter | Poaceae | Gashee | Herb | Fresh seed | O | Bone injury, Wound | St 2022 (46) |
| *Erica arborea* L. | Ericaceae | Satto | Shrub | Fresh root, Dry root, Dry leaf | O | Malaria, Spiritual, Wound | St 2022 (47) |
| *Corymbia citriodora* (Hook.) K.D.Hill & L.A.S.Johnson | Myrtaceae | Bargamo sayiti | Tree | Fresh leaf | O | Gonorrhea | St 2022 (48) |
| *Ficus sycomorus* L. | Moraceae | Odda | Tree | Dry seed, Dry bark, Fresh seed | O | Tonsillitis, Glandular | St 2022 (49) |
| *Helianthus annuus* L*.* | Asteraceae | Suffa | Herb | Fresh seed | O | Febrile illness, Tung infection | St 2022 (50) |
| *Indigofera arrecta* Hochst. ex A.Rich. | Fabaceae | Hinna | Shrub | Fresh leaf | O | General health | St 2022 (51) |
| *Kalanchoe densiflora* Rolfe | Crassulaceae | Hanculule Ancura | Herb | Fresh leaf | O | Muscular/joint pain | St 2022 (52) |
| *Kniphofia foliosa* Hochst. | Asphodelaceae | Shushune | Shrub | Fresh root | O | Stomachache | St 2022 (53) |
| *Lantana camara* L. | Verbenaceae | Qoso jarti | Shrub | Fresh leaf | O | Sneezing | St 2022 (54) |
| *Lippia abyssinica* (Otto & A.Dietr.) Cufod. | Verbenaceae | Sukayi | Herb | Dry leaf | O | Blood pressure, Diarrhea, Stomachache | St 2022 (55) |
| *Gymnosporia senegalensis* (Lam.) Loes. | Celastraceae | Kombolcha | Shrub | Fresh bark | O | Jaundice, Malaria, Skin infection | St 2022 (56) |
| *Mimusops kummel* Bruce ex A.DC. | Sapotaceae | Olaatee | Tree | Fresh seed, Dry seed | O | Diarrhea, Lung infection | St 2022 (57) |
| *Myrica salicifolia* Hochst. ex A.Rich. | Myricaceae | Qammo | Tree | Fresh bark | O | Spiritual | St 2022 (58) |
| *Pavonia urens* Cav. | Malvaceae | Hincinnii | Herb | Fresh leaf | O | Spiritual | St 2022 (59) |
| *Persicaria senegalensis* (Meisn.) Soják | Polygonaceae | Shulta | Herb | Fresh leaf | O | Jaundice, Malaria | St 2022 (60) |
| *Pittosporum viridiflorum* Sims | Pittosporaceae | Harbu | Tree | Fresh leaf, Fresh bark | O | Rabies, Fever, Spiritual | St 2022 (61) |
| *Plantago lanceolata* L. | Plantaginaceae | Qorxxo | Herb | Fresh root | O | Epilepsy | St 2022 (62) |
| *Aningeria altissima* (A.Chev.) Aubrév. & Pellegr. | Sapotaceae | Kore | Tree | Fresh bark | O | Swellings | St 2022 (63) |
| *Rubus apetalus* Poir. | Rosaceae | Goorra | Shrub | Young bud, Fresh seed | O | Toothache | St 2022 (64) |
| *Rubus steudneri* Schweinf. | Rosaceae | Goorra | Shrub | Dry bark, Fresh bark, Fresh root, Fresh leaf | O | Headache, Nasal bleeding,  Skin infection, Amoeba, Diarrhea, Urinary organ infection, Febrile illness, Stomachache | St 2022 (65) |
| *Salvia nilotica* Juss. ex Jacq. | Lamiaceae | Hulegebi | Herb | Ash | O | Heart case | St 2022 (66) |
| *Schinus molle* L. | Anacardiaceae | Qondo | Tree | Fresh leaf, Fresh seed, Young bud | O | Jaundice, Tonsillitis, Nasal bleeding | St 2022 (67) |
| *Schrebera alata* (Hochst.) Welw. | Oleaceae | Dhamma’e | Tree | Fresh root | O | Cancer, Swellings | St 2022 (68) |
| *Senna auriculata* (L.) Roxb. | Fabaceae | Ajawa | Shrub | Dry root, Fresh leaf | O | Constipation, Skin infection | St 2022 (69) |
| *Solanum marginatum* L.f. | Solanaceae | Hidhi oromo | Shrub | Fresh root, Fresh fruit, Fresh leaf | O | Febrile illness, Acid injury, Nasal bleeding, Snake poison, Autism, Spiritual | St 2022 (70) |
| *Vepris nobilis* (Delile) Mziray | Rutaceae | Hadhessa | Tree | Fresh leaf, Dry leaf, Fresh root | O | Blood pressure, Skin infection, Dry skin treatment, Ear infection, Eye infection | St 2022 (71) |
| *Terminalia brownii* Fresen. | Combretaceae | Rukessa | Shrub | Fresh leaf | O | Common cold, Headache | St 2022 (72) |
| *Trichilia dregeana* Sond. | Meliaceae | Sissa | Tree | Dry seed, Fresh leaf | O | Jaundice | St 2022 (73) |
| *Ximenia americana* L. | Olacaceae | Hudha | Shrub | Fresh seed, Fresh root | O | Swellings, Intestinal worms, Wound, Stomachache | St 2022 (74) |
| *Zea mays* L. | Poaceae | Badala | Herb | Dry seed | O | Sneezing | St 2022 (75) |
| *Ziziphus spina-christi* (L.) Willd. | Rhamnaceae | Qurqura | Tree | Fresh leaf, Fresh root, Dry seed, Fresh latex, Fresh bark | O | Skin infection, Spiritual, Rabies, Giardia, Gonorrhea, Eye infection, Intestinal worms, Wound | St 2022 (76) |
| *Aloe pirottae* A.Berger | Asphodelaceae | Sibri | Herb | Fresh leaf, Dry leaf | O, G | Gastric diseases, Jaundice, Kidney infection, Menstruation cycle disorder, Passive sexual interest, Vaginal infection, Ear infection | St 2022 (77) |
| *Asparagus africanus* Lam*.* | Asparagaceae | Siriti | Shrub | Fresh bark, Fresh leaf, Dry seed | O, G | Rabies, Breast cancer, Jaundice, Ear infection, Skin infection, Epilepsy, Swellings, Lung infection, Cancer | St 2022 (78) |
| *Brassica carinata* A.Braun | Brassicaceae | Shaaana | Herb | Fresh leaf, Dry seed | O, G | Constipation, Fever, Skin infection, Toothache, Cough, Lung infection | St 2022 (79) |
| *Commelina benghalensis* L. | Commelinaceae | Butikka Lalunxe | Herb | Fresh stolen, Fresh latex | O, G | Swellings, Amoeba, Skin infection | St 2022 (80) |
| *Delonix elata* (L.) Gamble | Fabaceae | Harangama | Shrub | Dry seed, Fresh seed | O, G | Stomachache | St 2022 (81) |
| *Euphorbia ampliphylla* Pax | Euphorbiaceae | Caree | Shrub | Fresh root, Fresh latex, Dry root | O, G | Epilepsy, Cancer, Spiritual | St 2022 (82) |
| *Grewia ferruginea* Hochst. ex A.Rich. | Malvaceae | Dhoqona | Shrub | Fresh bark, Fresh leaf, Dry leaf, Fresh root, Fresh seed | O, G | Respiratory organ infection, Jaundice, Febrile illness, Headache, Swellings, Wound, Epilepsy, Cancer, Amoeba | St 2022 (83) |
| *Hagenia abyssinica* (Bruce) J.F.Gmel. | Rosaceae | Hexxo | Tree | Dry seed, Fresh bark, Fresh root | O, G | Tapeworms, Amoeba, Diarrhea, Gonorrhea, Febrile illness, Intestinal worms | St 2022 (84) |
| *Hordeum vulgare* L. | Poaceae | Hayixxe | Herb | Fresh seed, Dry seed | O, G | Bone injury, Wound, Lightning | St 2022 (85) |
| *Lepidium sativum* L. | Brassicaceae | Fexxo Sinfa | Herb | Dry seed, Fresh root | O, G | Common cold, Febrile illness, Malaria, Vaginal infection, Dry skin treatment, Gastric diseases | St 2022 (86) |
| *Maesa lanceolata* Forssk. | Primulaceae | Abbaye | Shrub | Fresh root, Fresh leaf, Dry seed, Fresh bark | O, G | Jaundice, Nerve case, Muscular/joint pain, Skin infection, Gastric diseases, Ear infection, Amoeba, Gonorrhea, Cough | St 2022 (87) |
| *Nuxia congesta* R.Br. ex Fresen. | Stilbaceae | Burcana | Tree | Dry bark, Fresh bark | O, G | Cancer, Breast cancer, Skin infection, Wound | St 2022 (88) |
| *Ocimum gratissimum* L. | Lamiaceae | Qoricha michi | Shrub | Fresh leaf | O, G | Febrile illness, Fever, Eye infection, Vomiting, Malaria, Stomachache, Amoeba, Kidney infection | St 2022 (89) |
| *Prunus africana* (Hook.f.) Kalkman | Rosaceae | Garbicho | Tree | Fresh bark, Fresh seed | O, G | Skin infection, Glandular, Goiter | St 2022 (90) |
| *Psydrax schimperianus* (A.Rich.) Bridson | Rubiaceae | Gallo | Tree | Fresh bark, Fresh leaf, Young fresh leaf | O, G | Blood pressure, Febrile illness, Skin infection, Cancer, Wound, Muscle pain, Muscular/joint pain | St 2022 (91) |
| *Searsia pyroides* (Burch.) Moffett | Anacardiaceae | Dobobessa | Shrub | Fresh leaf, Fresh seed, Dry seed, Dry root | O, G | Breast cancer, Autism, Passive sexual interest, Common cold, Spiritual, Epilepsy | St 2022 (92) |
| *Sida schimperiana* Hochst. ex A.Rich. | Malvaceae | Koti jebessa | Shrub | Fresh root, Fresh bark, Dry leaf, Dry bark, Fresh leaf, Dry root | O, G | Swellings, Gonorrhea, Headache, Lung infection, Toothache, Wound, Fever, Jaundice, Glandular | St 2022 (93) |
| *Solanecio gigas* (Vatke) C.Jeffrey | Asteraceae | Dumbolla Yeshikoko gomen | Shrub | Fresh seed, Fresh leaf, Young root, Dry seed, Young fresh leaf, Fresh root | O, G | Lung infection, Gastric diseases, Jaundice, Malaria, Swellings, Amoeba, Diarrhea, Nasal bleeding, Glandular, Fever | St 2022 (94) |
| *Aframomum corrorima* (A.Braun) P.C.M.Jansen | Zingiberaceae | Janjiwello | Herb | Dry seed | S | Skin infection, Tonsillitis | St 2022 (95) |
| *Antiaris toxicaria* (J.F.Gmel.) Lesch. | Moraceae | Dimbicho | Tree | Fresh leaf, Fresh bark | S | Rabies | St 2022 (96) |
| *Artemisia absinthium* L. | Asteraceae | Arity | Herb | Fresh leaf | S | Diabetes, Spiritual | St 2022 (97) |
| *Commelina africana* L. | Commelinaceae | Lalunxe | Herb | Fresh leaf, Fresh latex | S | Skin infection | St 2022 (98) |
| *Coriandrum sativum* L. | Apiaceae | Dimbilale | Herb | Dry seed | S | Overall health | St 2022 (99) |
| *Cucumis dipsaceus* Ehrenb. ex Spach | Cucurbitaceae | Basu baqula | Climber | Fresh fruit | S | Jaundice | St 2022 (100) |
| *Cucumis prophetarum* L. | Cucurbitaceae | Basu baqula | Climber | Fresh fruit, Fresh root, Dry seed, Dry root | S | Cancer, Amoeba, Diarrhea, Lung infection, Jaundice, Rheumatic, Balanced diet, Glandular, Respiratory organ infection | St 2022 (101) |
| *Cynodon dactylon* (L.) Pers. | Poaceae | Qorcisha | Herb | Fresh stolon | S | Swellings | St 2022 (102) |
| *Cynoglossum coeruleum* Hochst. ex A.DC. | Boraginaceae | Hifaticho | Herb | Fresh leaf, Dry rhizome, Fresh root | S | Skin infection, Lung infection | St 2022 (103) |
| *Dovyalis caffra* (Hook.f. & Harv.) Warb. | Salicaceae | Faranjete shisho | Shrub | Fresh bark | S | Snake poison | St 2022 (104) |
| *Echinops kebericho* Mesfin | Asteraceae | Kebericho | Herb | Dry root, Fresh root | S | Common cold, Febrile illness, Headache, Fever | St 2022 (105) |
| *Euclea racemosa subsp. schimperi* (A.DC.) F.White | Ebenaceae | Mi’essa | Shrub | Dry bark | S | Stomachache | St 2022 (106) |
| *Vicia lens* (L.) Coss. & Germ. | Fabaceae | Misirra | Herb | Dry seed, Fresh seed | S | Chickenpox, Spider poison, Wound | St 2022 (107) |
| *Lippia javanica* (Burm.f.) Spreng. | Verbenaceae | Hanasho | Shrub | Fresh leaf | S | Blood pressure | St 2022 (108) |
| *Momordica boivinii* Baill. | Cucurbitaceae | Kiree | Climber | Fresh root, Fresh leaf, Fresh fruit, Dry seed | S | Spiritual, Lung infection, Jaundice, Stomachache, Toothache, Amoeba | St 2022 (109) |
| *Premna schimperi* Engl. | Lamiaceae | Uddo | Shrub | Fresh root, Fresh leaf | S | Lung infection, Febrile illness | St 2022 (110) |
| *Searsia natalensis* (Bernh. ex Krauss) F.A.Barkley | Anacardiaceae | Dawowesa | Tree | Fresh bark, Old root, Fresh leaf, Fresh seed | S | Snake poison | St 2022 (111) |
| *Triticum turgidum subsp. dicoccum* (Schrank ex Schübl.) Thell. | Poaceae | Ajja | Herb | Fresh seed | S | Wound | St 2022 (112) |
| *Ajuga integrifolia* Buch.-Ham. ex D.Don | Lamiaceae | Anamuro | Herb | Fresh leaf | S, G | Pain relief, Anemia, Stomachache, Malaria, Weight loss | St 2022 (113) |
| *Capsicum annuum* L. | Solanaceae | Mitmita | Herb | Fresh fruit, Fresh leaf | S, G | Intestinal worms, Anemia, Common cold, Tonsillitis | St 2022 (114) |
| *Cinnamomum verum* J.Presl | Lauraceae | Kerefa | Tree | Dry bark | S, G | Asthma, Common cold, Fever | St 2022 (115) |
| *Cucurbita pepo* L. | Cucurbitaceae | Baqulla | Climber | Dry seed | S, G | Tapeworms, Intestinal worms, Amoeba | St 2022 (116) |
| *Ensete ventricosum* (Welw.) Cheesman | Musaceae | Wesse | Herb | Fresh leaf, Young fresh leaf | S, G | Lightning, Swellings, Amoeba, Gastric diseases | St 2022 (117) |
| *Galinsoga quadriradiata* Ruiz & Pav. | Asteraceae | Qoricha | Herb | Fresh flower | S, G | Goiter, Tonsillitis, Toothache, Cancer, Swellings | St 2022 (118) |
| *Impatiens ethiopica* Grey-Wilson | Balsaminaceae | Enshoshila | Herb | Fresh root, Fresh leaf | S, G | Gonorrhea | St 2022 (119) |
| *Linum usitatissimum* L. | Linaceae | Telba | Herb | Dry seed, Dry rhizome | S, G | Gastric diseases, Blood pressure, Diabetes, Weight loss, Kidney infection, Cough, Lung infection, Tuberculosis | St 2022 (120) |
| *Coleus igniarius* Schweinf. | Lamiaceae | Tontona | Shrub | Fresh leaf, Fresh root, Dry rhizome | S, G | Amoeba, Skin infection, Febrile illness, Spiritual, Evil eye, Wound, Stomachache, Intestinal worms | St 2022 (121) |
| *Solanum nigrum* L. | Solanaceae | Awuxxi | Shrub | Fresh leaf, seed and fruit | S, G | Stomachache | St 2022 (122) |
| *Rumex nepalensis* Spreng. | Polygonaceae | Tulte | Herb | Fresh root, Dry leaf | S, G | Stomachache, Intestinal worms, Wound | St 2022 (123) |
| *Saccharum officinarum* L. | Poaceae | Sukari | Herb | Fresh stem | S, G | Gastric diseases | St 2022 (124) |
| *Thymus schimperi* Ronniger | Lamiaceae | Tosign | Herb | Dry leaf, Fresh leaf | S, G | Blood pressure, Cholesterol, Spiritual | St 2022 (125) |
| *Trigonella foenum-graecum* L. | Fabaceae | Abish | Herb | Dry seed, Fresh seed | S, G | Cholesterol, Blood pressure, Kidney infection, Cough, Lung infection, Tuberculosis, Abnormal menstruation cycle, Weight loss, Gastric diseases, Loss of appetite, Menstruation cycle disorder, Stomachache | St 2022 (126) |
| *Vicia faba* L. | Fabaceae |  | Herb | Fresh seed | S, G | Gastric diseases | St 2022 (127) |
| *Aloe vera* (L.) Burm.f. | Asphodelaceae | Argissa Algae | Herb | Fresh leaf, Fresh latex | S, O | Amoeba, Malaria, Blood pressure, Stomachache | St 2022 (128) |
| *Balanites aegyptiaca* (L.) Delile | Zygophyllaceae | Gidicho | Tree | Dry seed, Fresh bark, Fresh seed, Dry stem | S, O | Amoeba, Diarrhea, Stomachache, Mental case, Headache | St 2022 (129) |
| *Carissa spinarum* L. | Apocynaceae | Gora Hagamssa | Climber | Fresh root, Fresh seed, Fresh bark, Fresh leaf | S, O | Gonorrhea, Diarrhea, Spiritual, Febrile illness, Headache | St 2022 (130) |
| *Dodonaea viscosa subsp. angustifolia* (L.f.) J.G.West | Sapindaceae | Itancha | Shrub | Fresh leaf, Fresh bark, Fresh root | S, O | Lung infection, Headache, Bone injury, Circumcision wound, Gastric diseases, Stomachache | St 2022 (131) |
| *Euclea divinorum* Hiern | Ebenaceae | Mi’essa | Shrub | Fresh root, Dry leaf, Fresh leaf, Fresh bark | S, O | Intestinal worms, Skin infection, Weight loss, Circumcision wound | St 2022 (132) |
| *Mentha spicata* L. | Lamiaceae | Nana | Herb | Fresh leaf, Dry leaf | S, O | Blood pressure | St 2022 (133) |
| *Ocimum jamesii* Sebald | Lamiaceae | Ambibish a | Shrub | Fresh leaf | S, O | Febrile illness, Worms | St 2022 (134) |
| *Persea americana* Mill. | Lauraceae | Avocado | Tree | Dry seed | S, O | Passive sexual interest, Blood pressure, Amoeba | St 2022 (135) |
| *Pittosporum abyssinicum* Delile | Pittosporaceae | Boncho | Tree | Fresh leaf, Fresh bark | S, O | Tuberculosis, Intestinal worms | St 2022 (136) |
| *Rhamnus prinoides* L'Hér. | Rhamnaceae | Xaaddo | Shrub | Young bud, Old root, Fresh leaf, Fresh root, Fresh seed | S, O | Tonsillitis, Gonorrhea, Skin infection, Stomachache | St 2022 (137) |
| *Searsia glutinosa* (Hochst. ex A.Rich.) Moffett | Anacardiaceae | Oloncho | Tree | Fresh leaf, Fresh root, Dry bark, Fresh bark | S, O | Lung infection, Spiritual, Glandular | St 2022 (138) |
| *Rumex abyssinicus* Jacq. | Polygonaceae | Shishone | Herb | Fresh root, Fresh bark | S, O | Skin infection, Gastric diseases, Amoeba, Cancer | St 2022 (139) |
| *Solanum incanum* L. | Solanaceae | Borbodhicho | Shrub | Fresh leaf, Fresh root | S, O | Nasal bleeding, Snake poison, Spiritual, Diarrhea | St 2022 (140) |
| *Taverniera abyssinica* A.Rich. | Fabaceae | Dingetegn | Shrub | Dry rhizome, Fresh root, Dry stem | S, O | Febrile illness, Fever, Headache | St 2022 (141) |
| *Zehneria scabra* (L.f.) Sond. | Cucurbitaceae | Aba ejole Aba ejole | Climber | Dry seed, Fresh leaf, Fresh root | S, O | Cancer, Gastric diseases, Spiritual, Swellings | St 2022 (142) |
| *Achyranthes aspera* L*.* | Amaranthaceae | Maxxane Cikicho | Herb | Fresh root, Fresh leaf | S, O, G | Gonorrhea, Stomachache, Headache, Muscle pain, Joint pain, Cancer, Skin infection, Diarrhea, Respiratory organ infection, Spiritual, Jaundice, Lung infection, Ear infection, Nerve case | St 2022 (143) |
| *Acokanthera schimperi* (A.DC.) Benth. & Hook.f. ex Schweinf. | Apocynaceae | Qararo | Shrub | Dry seed, Fresh leaf, Fresh seed | S, O, G | Spiritual, Skin infection, Wound | St 2022 (144) |
| *Albizia gummifera* (J.F.Gmel.) C.A.Sm. | Fabaceae | Gorbe Maticho | Tree | Fresh bark, Dry bark, Fresh leaf, Fresh root | S, O, G | Cancer, Goiter, Toothache, Dizziness, Stomachache, Jaundice,  Lung infection, Amoeba, Malaria, Fire accident, Skin infection, Epilepsy, Febrile illness, Glandular, Gonorrhea, Swellings, Fever, Spiritual, Cough, Tuberculosis, Menstruation cycle disorder, Typhoid, Intestinal worms | St 2022 (145) |
| *Allium sativum* L. | Amaryllidaceae | Wajjo tuma | Herb | Fresh bulb | S, O, G | Common cold, Malaria, Typhoid, Fever, Headache, Febrile illness, Gonorrhea, Chicken pox, Tonsillitis, Blood pressure, Skin infection, Stomachache, Asthma, Tung infection | St 2022 (146) |
| *Aloe macrocarpa* Tod. | Asphodelaceae | Algae Hargissa | Herb | Fresh latex, Fresh leaf, Dry leaf | S, O, G | Malaria, Jaundice, Typhoid, Fever, Breast cancer, Lung infection, Gonorrhea, Pain relief, Urinary organ infection, Intestinal worms, Wound, Cancer, Stomachache, Diarrhea | St 2022 (147) |
| *Artemisia abyssinica* Sch.Bip. ex Oliv. & Hiern | Asteraceae | Ciqugn | Herb | Fresh leaf | S, O, G | Spiritual, Blood pressure, Malaria, Nasal bleeding, Chicken pox, Febrile illness, Skin infection, Bath of mother after giving birth, Headache | St 2022 (148) |
| *Bersama abyssinica* Fresen. | Francoaceae | Lolichissa Xewerako | Tree | Young bud, Fresh leaf, Fresh seed, Dry bark, Fresh bark, Young fresh leaf | S, O, G | Jaundice, Spiritual, Amoeba, Cancer, Intestinal worms, Skin infection, Stomachache, Lung infection | St 2022 (149) |
| *Brucea antidysenterica* J.F.Mill. | Simaroubaceae | Laffa, Abalcho (Waginos) | Tree | Fresh seed, Dry seed, Dry bark, Fresh bark | S, O, G | Gonorrhea, Diarrhea, Stomachache | St 2022 (150) |
| *Calpurnia aurea* (Aiton) Benth. | Fabaceae | Cekatta/Luxxa Cekata Cekatta | Shrub | Fresh leaf, Fresh seed, Dry seed, Dry root, Fresh root, Fresh bark, Dry leaf, Dry stem | S, O, G | Lung infection, Typhoid, Intestinal worms, Jaundice, Spiritual, Amoeba, Headache, Stomachache, Toothache, Fever, Skin infection, Wound, Circumcision wound, Febrile illness, Cancer, Bone cancer, Breast cancer, Swellings, Glandular, Respiratory organ infection | St 2022 (151) |
| *Carica papaya* L. | Caricaceae | Papaye | Tree | Dry seed, Fresh seed, Fresh fruit, Fresh leaf, Fresh latex | S, O, G | Malaria, Intestinal worms, Gastric diseases, Fever, Bath of mother after giving birth, Blood pressure, Cancer, Typhoid, Skin infection | St 2022 (152) |
| *Catha edulis* (Vahl) Forssk. ex Endl. | Celastraceae | Catte | Shrub | Fresh root, Fresh leaf, Young fresh leaf, Old root | S, O, G | Amoeba, Depression, Gonorrhea, Spiritual, Skin infection, Diarrhea | St 2022 (153) |
| *Citrus × aurantiifolia* (Christm.) Swingle | Rutaceae | Qomxaxxe | Shrub | Dry seed, Fresh fruit | S, O, G | Amoeba, Anemia, Blood pressure, Giardia | St 2022 (154) |
| *Clematis hirsuta* Perr. & Guill. | Ranunculaceae | Labbicha Fidhe Fittii | Climber | Fresh root, Fresh leaf | S, O, G | Jaundice, Cancer, Wound, Breast cancer, Ear infection | St 2022 (155) |
| *Clutia abyssinica* Jaub. & Spach | Peraceae | Binjile Ullefoni | Herb | Fresh root, Fresh seed, Whole parts, Fresh leaf, Fresh latex | S, O, G | Cancer, Diarrhea, Swellings, Spiritual, Snake poison, Bone cancer, Wound, Toothache, Breast cancer | St 2022 (156) |
| *Coffea arabica* L. | Rubiaceae | Bunna | Shrub | Dry seed, Dry leaf, Fresh leaf, Fresh bark, Fresh fruit bark | S, O, G | Gastric diseases, Malaria, Wound, Sneezing, Cancer, Kidney infection, Swellings, Jaundice, Breast cancer, Toothache, Depression, Gastric cancer | St 2022 (157) |
| *Cordia africana* Lam. | Boraginaceae | Wadicho, Wodessa | Tree | Fresh bark, Fresh seed, Dry seed | S, O, G | Nerve case, Passive sexual interest, Spiritual, Blood pressure, Diarrhea, Bone cancer | St 2022 (158) |
| *Croton macrostachyus* Hochst. ex Delile | Euphorbiaceae | Makkonissa Masina Mokonissa | Tree | Fresh bark, Fresh latex, Fresh leaf, Young bud, Dry bark, Fresh root, Dry leaf, Dry seed, Fresh seed, Leaf latex, Old root | S, O, G | Cancer, Eye infection, Lightning, Tetanus, Lung infection, Gonorrhea, Dizziness, Febrile illness, Wound, Spiritual, Diarrhea, Jaundice, Amoeba, Glandular, Giardia, Abortion, Intestinal worms, Malaria, Asthma, Breast cancer, Typhoid, Skin infection, Placental delay during birth, Circumcision wound, Stomachache, Ear infection, Allergy, Bone cancer, Menstruation cycle disorder | St 2022 (159) |
| *Datura stramonium* L. test | Solanaceae | Banje | Herb | Fresh leaf, Dry seed, Fresh root | S, O, G | Skin infection, Head skin infection, Toothache, Rabies | St 2022 (160) |
| *Ehretia cymosa* Thonn. | Boraginaceae | Gidincho | Tree | Fresh leaf, Fresh bark, Fresh seed | S, O, G | Wound, Nasal bleeding, Skin infection, Lung infection, Cancer, Stomachache, Swellings | St 2022 (161) |
| *Ekebergia capensis* Sparrm. | Meliaceae | Oloncho Onnonna | Tree | Dry seed, Fresh bark, Fresh seed, Fresh leaf, Dry rhizome, Root bark, Dry bark | S, O, G | Amoeba, Goiter, Jaundice, Gonorrhea, Tuberculosis, Typhoid, Fever, Stomachache, Cancer, Placental delay during birth, Skin infection, Spiritual, Swellings, Wound, Glandular, Bone cancer, Diarrhea, Febrile illness | St 2022 (162) |
| *Erythrina abyssinica* Lam. | Fabaceae | Welako Wallenu | Tree | Fresh bark, Fresh seed, Fresh leaf, Dry bark | S, O, G | Toothache, Spiritual, Diarrhea, Rabies, Intestinal worms, Lung infection, Goiter, Fever, Malaria, Eye infection, Cough, Skin infection, Tuberculosis, Liver infection | St 2022 (163) |
| *Eucalyptus globulus* Labill. | Myrtaceae | Wajo barzafe Bargamo addi | Tree | Fresh leaf | S, O, G | Asthma, Common cold, Pain relief, Bath of mother after giving birth, Fever, Mental case, Headache, Dry skin treatment, Skin infection, Spiritual, Nerve case, Nasal bleeding, Amoeba | St 2022 (164) |
| *Justicia schimperiana* (Hochst. ex Nees) T.Anderson | Acanthaceae | Cikicho Gulbana | Shrub | Fresh root, Fresh leaf, Old root | S, O, G | Amoeba, Rabies, Gonorrhea, Stomachache, Sneezing, Jaundice, Ear infection, Glandular, Goiter, Malaria, Epilepsy | St 2022 (165) |
| *Kalanchoe petitiana* A.Rich. | Crassulaceae | Hanculule | Herb | Fresh leaf, Fresh root | S, O, G | A broken bone, Pain relief, Muscular/joint pain, Glandular, Diarrhea, Bone injury | St 2022 (166) |
| *Lactuca inermis* Forssk. | Asteraceae | Ameessa Amessa | Herb | Fresh leaf, Whole parts | S, O, G | A balanced diet, Weight loss, Anemia, Febrile illness, Stomachache | St 2022 (167) |
| *Lagenaria siceraria* (Molina) Standl. | Cucurbitaceae | Surupha Basu baqula | Climber | Old root, Fresh root, Dry seed, Fresh fruit, Fresh leaf | S, O, G | Lung infection, Jaundice, Glandular, Fever, Joint pain, Amoeba, Goiter, Pain relief | St 2022 (168) |
| *Melia azedarach* L. | Meliaceae | Niim, Nimm | Tree | Fresh bark, Fresh leaf, Fresh seed, Dry root, Dry bark | S, O, G | Diabetes, Malaria, Stomachache, Depression, Diarrhea, Blood pressure, Gastric diseases, Nasal bleeding, Pain relief, Jaundice, Toothache, Cancer, Intestinal worms, Typhoid, Fever, Glandular, Breast cancer | St 2022 (169) |
| *Millettia ferruginea* (Hochst.) Hochst. ex Baker | Fabaceae | Hengedicho | Tree | Fresh bark, Fresh root, Fresh bulb, Fresh leaf, Dry bark | S, O, G | Amoeba, Gonorrhea, Typhoid, Skin infection, Blood pressure, Stomachache, Malaria, Cancer, Jaundice, Toothache, Ear infection, Goiter, Lung infection, Pain relief | St 2022 (170) |
| *Moringa stenopetala* (Baker f.) Cufod. | Moringaceae | Shiferaw | Tree | Dry leaf, Fresh bark, Fresh leaf, Fresh root, Dry bark | S, O, G | Blood pressure, Glandular, Jaundice, Malaria, Diarrhea, Kidney infection, Lung infection, Cancer, Gastric diseases, Cholesterol, Nerve case, Pain relief, Intestinal worms, Typhoid | St 2022 (171) |
| *Nicotiana tabacum* L. | Solanaceae | Araddo Arado | Herb | Dry leaf | S, O, G | Headache, Wound, Depression, Common cold | St 2022 (172) |
| *Nigella sativa* L. | Ranunculaceae | Wajjo azmude | Herb | Dry seed | S, O, G | Common cold, Respiratory organ infection, Febrile illness, Skin infection, Amoeba, Nasal bleeding, Fever, Malaria, Asthma, Pain relief, Stomachache, Nerve case, Bone injury, Cancer | St 2022 (173) |
| *Ocimum lamiifolium* Hochst. ex Benth. | Lamiaceae | Michete xagicho | Shrub | Fresh leaf, Fresh root | S, O, G | Headache, Malaria, Febrile illness, Fever, Stomachache, Muscular/joint pain, Amoeba, Gonorrhea, Typhoid, Diarrhea | St 2022 (174) |
| *Olea europaea subsp. cuspidata* (Wall. & G.Don) Cif. | Oleaceae | Ejerissa | Tree | Dry stem, Fresh seed, Fresh leaf, Fresh bark, Fresh root, Young petiole, Dry bark | S, O, G | Breast cancer, Skin infection, Wound, Anemia, Blood pressure, Cough, Malaria, Respiratory organ infection, Spiritual, Tuberculosis, Jaundice, Cancer, Swellings, Toothache, Kidney infection, Vaginal infection, Intestinal worms, Pain relief, Asthma | St 2022 (175) |
| *Olinia rochetiana* A.Juss. | Penaeaceae | Noole Gunna | Tree | Fresh leaf, Fresh bark, Dry leaf, Dry bark | S, O, G | Stomachache, Glandular, Skin infection, Wound, Circumcision wound, Toothache, Cancer, Tuberculosis | St 2022 (176) |
| *Phytolacca dodecandra* L'Hér. | Phytolaccaceae | Haranjicho | Shrub | Fresh root, Fresh leaf, Old root, Young root | S, O, G | Abortion, Amoeba, Intestinal worms, Gonorrhea, Swellings, Giardia, Stomachache, Skin infection | St 2022 (177) |
| *Afrocarpus falcatus* (Thunb.) C.N.Page | Podocarpaceae | Dagucho | Tree | Fresh leaf, Fresh bark, Dry bark | S, O, G | Gonorrhea, Typhoid, Malaria, Jaundice, Cancer, Wound, Breast cancer, Glandular, Toothache, Amoeba | St 2022 (178) |
| *Psidium guajava* L. | Myrtaceae | Zaytunna | Tree | Fresh leaf, Dry leaf | S, O, G | Blood pressure, Malaria, Diabetes, Stomachache, Cancer, Intestinal worms, Typhoid | St 2022 (179) |
| *Ricinus communis* L. | Euphorbiaceae | Qomboho | Shrub | Fresh root, Dry seed, Young root, Fresh seed | S, O, G | Jaundice, Lung infection, Swellings, Tonsillitis, Wound, Skin infection | St 2022 (180) |
| *Ruta chalepensis* L. | Rutaceae | Sunkurta | Herb | Fresh leaf, Fresh root, Fresh flower, Dry leaf | S, O, G | Gonorrhea, Typhoid, Febrile illness, Goiter, Tuberculosis, Skin infection, Diarrhea, Spiritual, Malaria, Vomiting, Nerve case, Dry skin treatment, Stomachache, Giardia, Jaundice, Nasal bleeding, Placental delay during birth, Epilepsy, Swellings, Asthma, Headache, Anemia, Glandular, Breast cancer, Bath of mother after giving a birth, Menstruation cycle disorder | St 2022 (181) |
| *Stephania abyssinica* (Quart.-Dill. & A.Rich.) Walp*.* | Menispermaceae | Kelala | Climber | Fresh leaf, Fresh root | S, O, G | Jaundice, Glandular, Lung infection, Gonorrhea, Cancer | St 2022 (123) |
| *Syzygium guineense* (Willd.) DC. | Myrtaceae | Badessa Duwancho Baddessa | Tree | Fresh bark, Fresh leaf, Dry root, Dry leaf, Fresh root, Young root | S, O, G | Amoeba, Diarrhea, Muscle pain, Spiritual, Skin infection, Lung infection, Weight loss, Glandular, Circumcision wound, Pain relief, Breast cancer, Swellings, Cancer | St 2022 (183) |
| *Urtica dioica* L. | Urticaceae | Lalesa | Herb | Fresh root, Dry root | S, O, G | Amoeba, Spiritual, Gonorrhea, Febrile illness, Cancer | St 2022 (184) |
| *Urtica simensis* Hochst. ex A.Rich. | Urticaceae | Sonicho | Herb | Fresh leaf, Fresh root, Dry root | S, O, G | Spiritual, Fire accident, Febrile illness, Gastric diseases, Amoeba, Intestinal worms, Stomachache | St 2022 (185) |
| *Gymnanthemum amygdalinum* (Delile) Sch.Bip. | Asteraceae | Hecho Ebicha | Shrub | Fresh leaf, Dry rhizome, Fresh root, Young root | S, O, G | Amoeba, Malaria, Skin infection, Stomachache, Diarrhea, Head skin infection, Gonorrhea, Rabies, Febrile illness, Intestinal worms, Gastric diseases, Lung infection, Blood pressure, Jaundice, Vomiting, Typhoid | St 2022 (186) |
| *Gymnanthemum auriculiferum* (Hiern) Isawumi | Asteraceae | Rejii | Shrub | Fresh leaf, Fresh root | S, O, G | Spiritual, Snake poison, Bath of mother after giving a birth | St 2022 (187) |
| *Withania somnifera* (L.) Dunal | Solanaceae | Bula | Shrub | Fresh leaf, Fresh bark, Dry seed, Fresh root, Dry bark, Dry stem, Old root | S, O, G | Spiritual, Asthma, Cough, Skin infection, Febrile illness | St 2022 (188) |
| *Zingiber officinale* Roscoe | Zingiberaceae | Janjiwello | Herb | Dry rhizome, Fresh rhizome | S, O, G | Asthma, Blood pressure, Passive sexual interest, Common cold, Tonsillitis, Typhoid, Headache, Malaria, Wound, Fever, Cough, Tung infection, Goiter, Constipation, Febrile illness, Amoeba, Gastric diseases, Stomachache | St 2022 (189) |
