# Supplementary material for: Ethnobotanical study on medicinal plant knowledge among three ethnic groups in peri-urban areas of south-central Ethiopia
Source: J Ethnobiol Ethnomed. 2023 Nov 23;19:55. doi: 10.1186/s13002-023-00629-w (PMC10668360; doi:10.1186/s13002-023-00629-w)
Supplement: Supplementary file 2 — Additional file 2. Rahman similarity index between Gedeo and Oromo ethnic groups. [file 13002_2023_629_MOESM2_ESM.docx]

Supplementary Table 2: Rahman similarity index between Gedeo and Oromo ethnic groups. ‘Yes’ indicates shared medicinal plants to treat the same ailments between the two ethnic groups. Whereas ‘Yes or No’ indicates a disparity between ethnic groups.

| **Species** | **Ailment** | **Ethnic groups** | |
| --- | --- | --- | --- |
|  |  | **Gedeo** | **Oromo** |
| *Achyranthes aspera* L. | Respiratory organ infection | Yes | Yes |
| *Acokanthera schimperi* (A.DC.) Benth. & Hook.f. ex Schweinf. | Spiritual | Yes | Yes |
| *Albizia gummifera* (J.F.Gmel.) C.A.Sm. | Dizziness | Yes | Yes |
| *Albizia gummifera* (J.F.Gmel.) C.A.Sm. | Febrile illness | Yes | Yes |
| *Albizia gummifera* (J.F.Gmel.) C.A.Sm. | Fire accident | Yes | Yes |
| *Albizia gummifera* (J.F.Gmel.) C.A.Sm. | Malaria | Yes | Yes |
| *Albizia gummifera* (J.F.Gmel.) C.A.Sm. | Skin infection | Yes | Yes |
| *Albizia gummifera* (J.F.Gmel.) C.A.Sm. | Stomachache | Yes | Yes |
| *Allium sativum* L. | Blood pressure | Yes | Yes |
| *Allium sativum* L. | Common cold | Yes | Yes |
| *Allium sativum* L. | Typhoid | Yes | Yes |
| *Aloe macrocarpa* Tod. | Gonorrhea | Yes | Yes |
| *Aloe pirottae* A.Berger | Passive sexual interest | Yes | Yes |
| *Artemisia abyssinica* Sch.Bip. ex A.Rich. | Spiritual | Yes | Yes |
| *Asparagus africanus* Lam*.* | Breast cancer | Yes | Yes |
| *Asparagus africanus* Lam*.* | Jaundice | Yes | Yes |
| *Asparagus africanus* Lam*.* | Skin infection | Yes | Yes |
| *Bersama abyssinica* Fresen. | Intestinal worms | Yes | Yes |
| *Brucea antidysenterica* J.F.Mill. | Diarrhea | Yes | Yes |
| *Calpurnia aurea* (Aiton) Benth. | Amoeba | Yes | Yes |
| *Calpurnia aurea* (Aiton) Benth. | Headache | Yes | Yes |
| *Calpurnia aurea* (Aiton) Benth. | Wound | Yes | Yes |
| *Carica papaya* L. | Cancer | Yes | Yes |
| *Carica papaya* L. | Malaria | Yes | Yes |
| *Carica papaya* L. | Typhoid | Yes | Yes |
| *Catha edulis* (Vahl) Forssk. ex Endl. | Diarrhea | Yes | Yes |
| *Catha edulis* (Vahl) Forssk. ex Endl. | Gonorrhea | Yes | Yes |
| *Citrus × aurantiifolia* (Christm.) Swingle | Giardia | Yes | Yes |
| *Clutia abyssinica* Jaub. & Spach | Cancer | Yes | Yes |
| *Coffea arabica* L. | Sneezing | Yes | Yes |
| *Coffea arabica* L. | Wound | Yes | Yes |
| *Cordia africana* Lam. | Passive sexual interest | Yes | Yes |
| *Croton macrostachyus* Hochst. ex Delile | Amoeba | Yes | Yes |
| *Croton macrostachyus* Hochst. ex Delile | Breast cancer | Yes | Yes |
| *Croton macrostachyus* Hochst. ex Delile | Cancer | Yes | Yes |
| *Croton macrostachyus* Hochst. ex Delile | Diarrhea | Yes | Yes |
| *Croton macrostachyus* Hochst. ex Delile | Dizziness | Yes | Yes |
| *Croton macrostachyus* Hochst. ex Delile | Eye infection | Yes | Yes |
| *Croton macrostachyus* Hochst. ex Delile | Febrile illness | Yes | Yes |
| *Croton macrostachyus* Hochst. ex Delile | Gonorrhea | Yes | Yes |
| *Croton macrostachyus* Hochst. ex Delile | Intestinal worms | Yes | Yes |
| *Croton macrostachyus* Hochst. ex Delile | Malaria | Yes | Yes |
| *Croton macrostachyus* Hochst. ex Delile | Skin infection | Yes | Yes |
| *Croton macrostachyus* Hochst. ex Delile | Stomachache | Yes | Yes |
| *Croton macrostachyus* Hochst. ex Delile | Typhoid | Yes | Yes |
| *Croton macrostachyus* Hochst. ex Delile | Wound | Yes | Yes |
| *Datura stramonium* L. test | Rabies | Yes | Yes |
| *Delonix elata* (L.) Gamble | Stomachache | Yes | Yes |
| *Ekebergia capensis* Sparrm. | Stomachache | Yes | Yes |
| *Ekebergia capensis* Sparrm. | Swellings | Yes | Yes |
| *Erythrina abyssinica* Lam. | Lung infection | Yes | Yes |
| *Eucalyptus globulus* Labill. | Asthma | Yes | Yes |
| *Eucalyptus globulus* Labill. | Bath of mother after giving a birth | Yes | Yes |
| *Eucalyptus globulus* Labill. | Common cold | Yes | Yes |
| *Eucalyptus globulus* Labill. | Nasal bleeding | Yes | Yes |
| *Justicia schimperiana* (Hochst. ex Nees) T.Anderson | Glandular | Yes | Yes |
| *Justicia schimperiana* (Hochst. ex Nees) T.Anderson | Goiter | Yes | Yes |
| *Justicia schimperiana* (Hochst. ex Nees) T.Anderson | Jaundice | Yes | Yes |
| *Kalanchoe petitiana* A.Rich. | Bone injury | Yes | Yes |
| *Lactuca inermis* Forssk. | Anemia | Yes | Yes |
| *Lactuca inermis* Forssk. | Stomachache | Yes | Yes |
| *Lactuca inermis* Forssk. | Weight loss | Yes | Yes |
| *Melia azedarach* L. | Jaundice | Yes | Yes |
| *Melia azedarach* L. | Skin infection | Yes | Yes |
| *Melia azedarach* L. | Blood pressure | Yes | Yes |
| *Melia azedarach* L. | Depression | Yes | Yes |
| *Melia azedarach* L. | Diarrhea | Yes | Yes |
| *Melia azedarach* L. | Stomachache | Yes | Yes |
| *Moringa stenopetala* (Baker f.) Cufod. | Blood pressure | Yes | Yes |
| *Moringa stenopetala* (Baker f.) Cufod. | Cancer | Yes | Yes |
| *Moringa stenopetala* (Baker f.) Cufod. | Glandular | Yes | Yes |
| *Moringa stenopetala* (Baker f.) Cufod. | Jaundice | Yes | Yes |
| *Moringa stenopetala* (Baker f.) Cufod. | Kidney infection | Yes | Yes |
| *Nigella sativa* L. | Asthma | Yes | Yes |
| *Nigella sativa* L. | Fever | Yes | Yes |
| *Nigella sativa* L. | Nasal bleeding | Yes | Yes |
| *Nigella sativa* L. | Stomachache | Yes | Yes |
| *Ocimum lamiifolium* Hochst. ex Benth. | Febrile illness | Yes | Yes |
| *Ocimum gratissimum* L. | Febrile illness | Yes | Yes |
| *Ocimum gratissimum* L. | Fever | Yes | Yes |
| *Ocimum gratissimum* L. | Malaria | Yes | Yes |
| *Ocimum gratissimum* L. | Stomachache | Yes | Yes |
| *Olea europaea subsp. cuspidata* (Wall. & G.Don) Cif. | Blood pressure | Yes | Yes |
| *Olea europaea subsp. cuspidata* (Wall. & G.Don) Cif. | Breast cancer | Yes | Yes |
| *Olea europaea subsp. cuspidata* (Wall. & G.Don) Cif. | Cancer | Yes | Yes |
| *Olea europaea subsp. cuspidata* (Wall. & G.Don) Cif. | Toothache | Yes | Yes |
| *Olea europaea subsp. cuspidata* (Wall. & G.Don) Cif. | Vaginal infection | Yes | Yes |
| *Olinia rochetiana* A.Juss. | Glandular | Yes | Yes |
| *Phytolacca dodecandra* L'Hér. | Abortion | Yes | Yes |
| *Phytolacca dodecandra* L'Hér. | Intestinal worms | Yes | Yes |
| *Phytolacca dodecandra* L'Hér. | Stomachache | Yes | Yes |
| *Afrocarpus falcatus* (Thunb.) C.N.Page | Typhoid | Yes | Yes |
| *Psidium guajava* L. | Blood pressure | Yes | Yes |
| *Ricinus communis* L. | Swellings | Yes | Yes |
| *Ruta chalepensis* L. | Febrile illness | Yes | Yes |
| *Ruta chalepensis* L. | Gonorrhea | Yes | Yes |
| *Ruta chalepensis* L. | Jaundice | Yes | Yes |
| *Ruta chalepensis* L. | Malaria | Yes | Yes |
| *Ruta chalepensis* L. | Nasal bleeding | Yes | Yes |
| *Ruta chalepensis* L. | Stomachache | Yes | Yes |
| *Ruta chalepensis* L. | Typhoid | Yes | Yes |
| *Ruta chalepensis* L. | Vomiting | Yes | Yes |
| *Sida schimperiana* Hochst. ex A.Rich. | Headache | Yes | Yes |
| *Solanecio gigas* (Vatke) C.Jeffrey | Lung infection | Yes | Yes |
| *Syzygium guineense* (Willd.) DC. | Glandular | Yes | Yes |
| *Gymnanthemum amygdalinum* (Delile) Sch.Bip. | Amoeba | Yes | Yes |
| *Gymnanthemum amygdalinum* (Delile) Sch.Bip. | Diarrhea | Yes | Yes |
| *Gymnanthemum amygdalinum* (Delile) Sch.Bip. | Intestinal worms | Yes | Yes |
| *Gymnanthemum amygdalinum* (Delile) Sch.Bip. | Malaria | Yes | Yes |
| *Gymnanthemum amygdalinum* (Delile) Sch.Bip. | Rabies | Yes | Yes |
| *Gymnanthemum amygdalinum* (Delile) Sch.Bip. | Skin infection | Yes | Yes |
| *Gymnanthemum amygdalinum* (Delile) Sch.Bip. | Stomachache | Yes | Yes |
| *Gymnanthemum auriculiferum* (Hiern) Isawumi | Snake poison | Yes | Yes |
| *Withania somnifera* (L.) Dunal | Spiritual | Yes | Yes |
| *Zingiber officinale* Roscoe | Common cold | Yes | Yes |
| *Zingiber officinale* Roscoe | Tonsillitis | Yes | Yes |
| *Zingiber officinale* Roscoe | Wound | Yes | Yes |
| *Achyranthes aspera* L. | Ear infection | Yes | No |
| *Achyranthes aspera* L. | Jaundice | Yes | No |
| *Achyranthes aspera* L. | Lung infection | Yes | No |
| *Achyranthes aspera* L. | Nerve case | Yes | No |
| *Ajuga integrifolia* Buch.-Ham. ex D.Don | Stomachache | Yes | No |
| *Albizia gummifera* (J.F.Gmel.) C.A.Sm. | Amoeba | Yes | No |
| *Albizia gummifera* (J.F.Gmel.) C.A.Sm. | Cancer | Yes | No |
| *Albizia gummifera* (J.F.Gmel.) C.A.Sm. | Cough | Yes | No |
| *Albizia gummifera* (J.F.Gmel.) C.A.Sm. | Fever | Yes | No |
| *Albizia gummifera* (J.F.Gmel.) C.A.Sm. | Glandular | Yes | No |
| *Albizia gummifera* (J.F.Gmel.) C.A.Sm. | Gonorrhea | Yes | No |
| *Albizia gummifera* (J.F.Gmel.) C.A.Sm. | Intestinal worms | Yes | No |
| *Albizia gummifera* (J.F.Gmel.) C.A.Sm. | Lung infection | Yes | No |
| *Albizia gummifera* (J.F.Gmel.) C.A.Sm. | Menstruation cycle disorder | Yes | No |
| *Albizia gummifera* (J.F.Gmel.) C.A.Sm. | Spiritual | Yes | No |
| *Albizia gummifera* (J.F.Gmel.) C.A.Sm. | Swellings | Yes | No |
| *Albizia gummifera* (J.F.Gmel.) C.A.Sm. | Toothache | Yes | No |
| *Albizia gummifera* (J.F.Gmel.) C.A.Sm. | Tuberculosis | Yes | No |
| *Albizia gummifera* (J.F.Gmel.) C.A.Sm. | Typhoid | Yes | No |
| *Allium cepa* L. | Nasal bleeding | Yes | No |
| *Allium cepa* L. | Passive sexual interest | Yes | No |
| *Allium cepa* L. | Weight loss | Yes | No |
| *Allium sativum* L. | Asthma | Yes | No |
| *Allium sativum* L. | Skin infection | Yes | No |
| *Allium sativum* L. | Stomachache | Yes | No |
| *Allium sativum* L. | Tung infection | Yes | No |
| *Aloe pirottae* A.Berger | Ear infection | Yes | No |
| *Ananas comosus* (L.) Merr. | Skin infection | Yes | No |
| *Artemisia abyssinica* Sch.Bip. ex A.Rich. | Bath of mother after giving a birth | Yes | No |
| *Artemisia abyssinica* Sch.Bip. ex A.Rich. | Chicken pox | Yes | No |
| *Artemisia abyssinica* Sch.Bip. ex A.Rich. | Febrile illness | Yes | No |
| *Artemisia abyssinica* Sch.Bip. ex A.Rich. | Headache | Yes | No |
| *Artemisia abyssinica* Sch.Bip. ex A.Rich. | Skin infection | Yes | No |
| *Oldeania alpina* (K.Schum.) Stapleton | Wound | Yes | No |
| *Arundo donax* L. | Swellings | Yes | No |
| *Asparagus africanus* Lam*.* | Cancer | Yes | No |
| *Asparagus africanus* Lam*.* | Epilepsy | Yes | No |
| *Asparagus africanus* Lam*.* | Lung infection | Yes | No |
| *Asparagus africanus* Lam*.* | Swellings | Yes | No |
| *Bersama abyssinica* Fresen. | Lung infection | Yes | No |
| *Bersama abyssinica* Fresen. | Skin infection | Yes | No |
| *Bersama abyssinica* Fresen. | Stomachache | Yes | No |
| *Bidens macroptera* (Sch.Bip. ex Chiov.) Mesfin. | Abnormal menstruation cycle | Yes | No |
| *Bidens macroptera* (Sch.Bip. ex Chiov.) Mesfin. | Amoeba | Yes | No |
| *Bidens macroptera* (Sch.Bip. ex Chiov.) Mesfin. | Diarrhea | Yes | No |
| *Brassica carinata* A.Braun | Cough | Yes | No |
| *Brassica carinata* A.Braun | Lung infection | Yes | No |
| *Brucea antidysenterica* J.F.Mill. | Stomachache | Yes | No |
| *Calpurnia aurea* (Aiton) Benth. | Bone cancer | Yes | No |
| *Calpurnia aurea* (Aiton) Benth. | Breast cancer | Yes | No |
| *Calpurnia aurea* (Aiton) Benth. | Cancer | Yes | No |
| *Calpurnia aurea* (Aiton) Benth. | Febrile illness | Yes | No |
| *Calpurnia aurea* (Aiton) Benth. | Glandular | Yes | No |
| *Calpurnia aurea* (Aiton) Benth. | Intestinal worms | Yes | No |
| *Calpurnia aurea* (Aiton) Benth. | Jaundice | Yes | No |
| *Calpurnia aurea* (Aiton) Benth. | Respiratory organ infection | Yes | No |
| *Calpurnia aurea* (Aiton) Benth. | Spiritual | Yes | No |
| *Calpurnia aurea* (Aiton) Benth. | Swellings | Yes | No |
| *Capsicum annuum* L. | Anemia | Yes | No |
| *Capsicum annuum* L. | Common cold | Yes | No |
| *Capsicum annuum* L. | Tonsillitis | Yes | No |
| *Capsicum frutescens* L. | Amoeba | Yes | No |
| *Capsicum frutescens* L. | Intestinal worms | Yes | No |
| *Carica papaya* L. | Skin infection | Yes | No |
| *Catha edulis* (Vahl) Forssk. ex Endl. | Depression | Yes | No |
| *Celtis africana* Burm.f. | Asthma | Yes | No |
| *Celtis africana* Burm.f. | Diarrhea | Yes | No |
| *Celtis africana* Burm.f. | Giardia | Yes | No |
| *Celtis africana* Burm.f. | Glandular | Yes | No |
| *Celtis africana* Burm.f. | Headache | Yes | No |
| *Celtis africana* Burm.f. | Intestinal worms | Yes | No |
| *Celtis africana* Burm.f. | Jaundice | Yes | No |
| *Celtis africana* Burm.f. | Lung infection | Yes | No |
| *Celtis africana* Burm.f. | Skin infection | Yes | No |
| *Celtis africana* Burm.f. | Stomachache | Yes | No |
| *Celtis africana* Burm.f. | Wound | Yes | No |
| *Cinnamomum verum* J.Presl | Asthma | Yes | No |
| *Cinnamomum verum* J.Presl | Common cold | Yes | No |
| *Clausena anisata* (Willd.) Hook.f. ex Benth. | Swellings | Yes | No |
| *Clematis* hirsuta Perr. & Guill. | Breast cancer | Yes | No |
| *Clematis* hirsuta Perr. & Guill. | Ear infection | Yes | No |
| *Clutia abyssinica* Jaub. & Spach | Breast cancer | Yes | No |
| *Clutia abyssinica* Jaub. & Spach | Toothache | Yes | No |
| *Clutia lanceolata* Forssk*.* | Ear infection | Yes | No |
| *Coffea arabica* L. | Breast cancer | Yes | No |
| *Coffea arabica* L. | Depression | Yes | No |
| *Coffea arabica* L. | Gastric cancer | Yes | No |
| *Coffea arabica* L. | Jaundice | Yes | No |
| *Coffea arabica* L. | Swellings | Yes | No |
| *Coffea arabica* L. | Toothache | Yes | No |
| *Colocasia esculenta* (L.) Schott | Cancer | Yes | No |
| *Colocasia esculenta* (L.) Schott | Toothache | Yes | No |
| *Commelina benghalensis* L. | Amoeba | Yes | No |
| *Commelina benghalensis* L. | Skin infection | Yes | No |
| *Cordia africana* Lam. | Bone cancer | Yes | No |
| *Croton macrostachyus* Hochst. ex Delile | Allergy | Yes | No |
| *Croton macrostachyus* Hochst. ex Delile | Bone cancer | Yes | No |
| *Croton macrostachyus* Hochst. ex Delile | Ear infection | Yes | No |
| *Croton macrostachyus* Hochst. ex Delile | Glandular | Yes | No |
| *Croton macrostachyus* Hochst. ex Delile | Lightning | Yes | No |
| *Croton macrostachyus* Hochst. ex Delile | Menstruation cycle disorder | Yes | No |
| *Croton macrostachyus* Hochst. ex Delile | Spiritual | Yes | No |
| *Cucurbita pepo* L. | Amoeba | Yes | No |
| *Cucurbita pepo* L. | Intestinal worms | Yes | No |
| *Cymbopogon citratus* (DC.) Stapf | Abortion | Yes | No |
| *Cymbopogon citratus* (DC.) Stapf | Bath of mother after giving a birth | Yes | No |
| *Cymbopogon citratus* (DC.) Stapf | Blood pressure | Yes | No |
| *Cymbopogon citratus* (DC.) Stapf | Cancer | Yes | No |
| *Cymbopogon citratus* (DC.) Stapf | Cholesterol | Yes | No |
| *Cymbopogon citratus* (DC.) Stapf | Gonorrhea | Yes | No |
| *Cymbopogon citratus* (DC.) Stapf | Kidney infection | Yes | No |
| *Cymbopogon citratus* (DC.) Stapf | Stomachache | Yes | No |
| *Cymbopogon citratus* (DC.) Stapf | Vomiting | Yes | No |
| *Datura stramonium* L. test | Head skin infection | Yes | No |
| *Dalbergia lactea* Vatke | Amoeba | Yes | No |
| *Dalbergia lactea* Vatke | Gonorrhea | Yes | No |
| *Drynaria volkensii* Heiron. | Cancer | Yes | No |
| *Drynaria volkensii* Heiron. | Ear infection | Yes | No |
| *Drynaria volkensii* Heiron. | Nasal bleeding | Yes | No |
| *Drynaria volkensii* Heiron. | Swellings | Yes | No |
| *Ehretia cymosa* Thonn. | Swellings | Yes | No |
| *Ekebergia capensis* Sparrm. | Amoeba | Yes | No |
| *Ekebergia capensis* Sparrm. | Diarrhea | Yes | No |
| *Ekebergia capensis* Sparrm. | Febrile illness | Yes | No |
| *Embelia schimperi* Vatke | Glandular | Yes | No |
| *Embelia schimperi* Vatke | Gonorrhea | Yes | No |
| *Embelia schimperi* Vatke | Jaundice | Yes | No |
| *Ensete ventricosum* (Welw.) Cheesman | Amoeba | Yes | No |
| *Ensete ventricosum* (Welw.) Cheesman | Gastric diseases | Yes | No |
| *Ensete ventricosum* (Welw.) Cheesman | Lightning | Yes | No |
| *Ensete ventricosum* (Welw.) Cheesman | Swellings | Yes | No |
| *Erythrina abyssinica* Lam. | Cough | Yes | No |
| *Erythrina abyssinica* Lam. | Eye infection | Yes | No |
| *Erythrina abyssinica* Lam. | Fever | Yes | No |
| *Erythrina abyssinica* Lam. | Liver infection | Yes | No |
| *Erythrina abyssinica* Lam. | Malaria | Yes | No |
| *Erythrina abyssinica* Lam. | Skin infection | Yes | No |
| *Erythrina abyssinica* Lam. | Tuberculosis | Yes | No |
| *Eucalyptus globulus* Labill. | Amoeba | Yes | No |
| *Euphorbia ampliphylla* Pax | Spiritual | Yes | No |
| *Euphorbia pulcherrima* Willd. ex Klotzsch | Fever | Yes | No |
| *Euphorbia tirucalli* L. | Cancer | Yes | No |
| *Fagaropsis angolensis* (Engl.) H.M.Gardner | Stomachache | Yes | No |
| *Fagaropsis angolensis* (Engl.) H.M.Gardner | Swellings | Yes | No |
| *Fagaropsis angolensis* (Engl.) H.M.Gardner | Wound | Yes | No |
| *Flacourtia indica* (Burm.f.) Merr. | Respiratory organ infection | Yes | No |
| *Flacourtia indica* (Burm.f.) Merr. | Snake poison | Yes | No |
| *Galinsoga quadriradiata* Ruiz & Pav. | Cancer | Yes | No |
| *Galinsoga quadriradiata* Ruiz & Pav. | Swellings | Yes | No |
| *Galinsoga quadriradiata* Ruiz & Pav. | Tonsillitis | Yes | No |
| *Galinsoga quadriradiata* Ruiz & Pav. | Toothache | Yes | No |
| *Grewia ferruginea* Hochst. ex A.Rich. | Amoeba | Yes | No |
| *Grewia ferruginea* Hochst. ex A.Rich. | Cancer | Yes | No |
| *Grewia ferruginea* Hochst. ex A.Rich. | Epilepsy | Yes | No |
| *Grewia ferruginea* Hochst. ex A.Rich. | Febrile illness | Yes | No |
| *Grewia ferruginea* Hochst. ex A.Rich. | Headache | Yes | No |
| *Grewia ferruginea* Hochst. ex A.Rich. | Jaundice | Yes | No |
| *Grewia ferruginea* Hochst. ex A.Rich. | Swellings | Yes | No |
| *Grewia ferruginea* Hochst. ex A.Rich. | Wound | Yes | No |
| *Hagenia abyssinica* (Bruce) J.F.Gmel. | Intestinal worms | Yes | No |
| *Hibiscus macranthus* Hochst. ex A. Rich. | Fire accident | Yes | No |
| *Hordeum vulgare* L. | Lightning | Yes | No |
| *Hyparrhenia rufa* (Nees) Stapf | Cough | Yes | No |
| *Hyparrhenia rufa* (Nees) Stapf | Lung infection | Yes | No |
| *Hyparrhenia rufa* (Nees) Stapf | Swellings | Yes | No |
| *Impatiens ethiopica* Grey-Wilson | Gonorrhea | Yes | No |
| *Juniperus procera* Hochst. ex Endl. | Respiratory organ infection | Yes | No |
| *Justicia schimperiana* (Hochst. ex Nees) T.Anderson | Epilepsy | Yes | No |
| *Justicia schimperiana* (Hochst. ex Nees) T.Anderson | Malaria | Yes | No |
| *Kanahia laniflora* (Forssk.) R.Br. | Jaundice | Yes | No |
| *Lagenaria siceraria* (Molina) Standl. | Amoeba | Yes | No |
| *Lagenaria siceraria* (Molina) Standl. | Goiter | Yes | No |
| *Lagenaria siceraria* (Molina) Standl. | Jaundice | Yes | No |
| *Lagenaria siceraria* (Molina) Standl. | Pain relief | Yes | No |
| *Lepidium sativum* L. | Gastric diseases | Yes | No |
| *Leucas tomentosa* Gürke | Febrile illness | Yes | No |
| *Linum usitatissimum* L. | Cough | Yes | No |
| *Linum usitatissimum* L. | Lung infection | Yes | No |
| *Linum usitatissimum* L. | Tuberculosis | Yes | No |
| *Maesa lanceolata* Forssk. | Amoeba | Yes | No |
| *Maesa lanceolata* Forssk. | Cough | Yes | No |
| *Maesa lanceolata* Forssk. | Ear infection | Yes | No |
| *Maesa lanceolata* Forssk. | Gastric diseases | Yes | No |
| *Maesa lanceolata* Forssk. | Gonorrhea | Yes | No |
| *Melia azedarach* L. | Breast cancer | Yes | No |
| *Melia azedarach* L. | Malaria | Yes | No |
| *Millettia ferruginea* (Hochst.) Hochst. ex Baker | Amoeba | Yes | No |
| *Millettia ferruginea* (Hochst.) Hochst. ex Baker | Cancer | Yes | No |
| *Millettia ferruginea* (Hochst.) Hochst. ex Baker | Ear infection | Yes | No |
| *Millettia ferruginea* (Hochst.) Hochst. ex Baker | Goiter | Yes | No |
| *Millettia ferruginea* (Hochst.) Hochst. ex Baker | Jaundice | Yes | No |
| *Millettia ferruginea* (Hochst.) Hochst. ex Baker | Lung infection | Yes | No |
| *Millettia ferruginea* (Hochst.) Hochst. ex Baker | Malaria | Yes | No |
| *Millettia ferruginea* (Hochst.) Hochst. ex Baker | Pain relief | Yes | No |
| *Millettia ferruginea* (Hochst.) Hochst. ex Baker | Skin infection | Yes | No |
| *Millettia ferruginea* (Hochst.) Hochst. ex Baker | Toothache | Yes | No |
| *Nicotiana tabacum* L. | Headache | Yes | No |
| *Nigella sativa* L. | Bone injury | Yes | No |
| *Nigella sativa* L. | Cancer | Yes | No |
| *Nigella sativa* L. | Respiratory organ infection | Yes | No |
| *Nuxia congesta* R.Br. ex Fresen. | Breast cancer | Yes | No |
| *Nuxia congesta* R.Br. ex Fresen. | Skin infection | Yes | No |
| *Nuxia congesta* R.Br. ex Fresen. | Wound | Yes | No |
| *Ocimum lamiifolium* Hochst. ex Benth. | Amoeba | Yes | No |
| *Ocimum lamiifolium* Hochst. ex Benth. | Diarrhea | Yes | No |
| *Ocimum lamiifolium* Hochst. ex Benth. | Gonorrhea | Yes | No |
| *Ocimum lamiifolium* Hochst. ex Benth. | Muscular/joint pain | Yes | No |
| *Ocimum lamiifolium* Hochst. ex Benth. | Typhoid | Yes | No |
| *Ocimum gratissimum* L. | Amoeba | Yes | No |
| *Ocimum gratissimum* L. | Kidney infection | Yes | No |
| *Olea europaea subsp. cuspidata* (Wall. & G.Don) Cif. | Skin infection | Yes | No |
| *Phytolacca dodecandra* L'Hér. | Giardia | Yes | No |
| *Phytolacca dodecandra* L'Hér. | Skin infection | Yes | No |
| *Coleus igniarius* Schweinf. | Intestinal worms | Yes | No |
| *Coleus igniarius* Schweinf. | Stomachache | Yes | No |
| *Afrocarpus falcatus* (Thunb.) C.N.Page | Amoeba | Yes | No |
| *Afrocarpus falcatus* (Thunb.) C.N.Page | Breast cancer | Yes | No |
| *Afrocarpus falcatus* (Thunb.) C.N.Page | Cancer | Yes | No |
| *Afrocarpus falcatus* (Thunb.) C.N.Page | Glandular | Yes | No |
| *Afrocarpus falcatus* (Thunb.) C.N.Page | Gonorrhea | Yes | No |
| *Afrocarpus falcatus* (Thunb.) C.N.Page | Jaundice | Yes | No |
| *Afrocarpus falcatus* (Thunb.) C.N.Page | Malaria | Yes | No |
| *Afrocarpus falcatus* (Thunb.) C.N.Page | Toothache | Yes | No |
| *Afrocarpus falcatus* (Thunb.) C.N.Page | Wound | Yes | No |
| *Prunus africana* (Hook.f.) Kalkman | Glandular | Yes | No |
| *Prunus africana* (Hook.f.) Kalkman | Goiter | Yes | No |
| *Psidium guajava* L. | Malaria | Yes | No |
| *Psydrax schimperianus* (A.Rich.) Bridson | Muscle pain | Yes | No |
| *Psydrax schimperianus* (A.Rich.) Bridson | Muscular/joint pain | Yes | No |
| *Searsia pyroides* (Burch.) Moffett | Common cold | Yes | No |
| *Searsia pyroides* (Burch.) Moffett | Epilepsy | Yes | No |
| *Searsia pyroides* (Burch.) Moffett | Spiritual | Yes | No |
| *Ricinus communis* L. | Skin infection | Yes | No |
| *Ricinus communis* L. | Tonsillitis | Yes | No |
| *Ricinus communis* L. | Wound | Yes | No |
| *Rotheca myricoides* (Hochst.) Steane & Mabb. | Stomachache | Yes | No |
| *Rubia cordifolia* L. | Malaria | Yes | No |
| *Rumex nepalensis* Spreng. | Intestinal worms | Yes | No |
| *Rumex nepalensis* Spreng. | Wound | Yes | No |
| *Ruta chalepensis* L. | Anemia | Yes | No |
| *Ruta chalepensis* L. | Asthma | Yes | No |
| *Ruta chalepensis* L. | Bath of mother after giving a birth | Yes | No |
| *Ruta chalepensis* L. | Breast cancer | Yes | No |
| *Ruta chalepensis* L. | Epilepsy | Yes | No |
| *Ruta chalepensis* L. | Glandular | Yes | No |
| *Ruta chalepensis* L. | Headache | Yes | No |
| *Ruta chalepensis* L. | Menstruation cycle disorder | Yes | No |
| *Ruta chalepensis* L. | Swellings | Yes | No |
| *Saccharum officinarum* L. | Gastric diseases | Yes | No |
| *Sesbania sesban* (L.) Merr. | Rabies | Yes | No |
| *Sesbania sesban* (L.) Merr. | Snake poison | Yes | No |
| *Sida ovata* Forssk. | Bone cancer | Yes | No |
| *Sida schimperiana* Hochst. ex A.Rich. | Fever | Yes | No |
| *Sida schimperiana* Hochst. ex A.Rich. | Glandular | Yes | No |
| *Sida schimperiana* Hochst. ex A.Rich. | Jaundice | Yes | No |
| *Sida schimperiana* Hochst. ex A.Rich. | Toothache | Yes | No |
| *Sida schimperiana* Hochst. ex A.Rich. | Wound | Yes | No |
| *Solanecio gigas* (Vatke) C.Jeffrey | Amoeba | Yes | No |
| *Solanecio gigas* (Vatke) C.Jeffrey | Diarrhea | Yes | No |
| *Solanecio gigas* (Vatke) C.Jeffrey | Fever | Yes | No |
| *Solanecio gigas* (Vatke) C.Jeffrey | Gastric diseases | Yes | No |
| *Solanecio gigas* (Vatke) C.Jeffrey | Glandular | Yes | No |
| *Solanecio gigas* (Vatke) C.Jeffrey | Jaundice | Yes | No |
| *Solanecio gigas* (Vatke) C.Jeffrey | Malaria | Yes | No |
| *Solanecio gigas* (Vatke) C.Jeffrey | Nasal bleeding | Yes | No |
| *Solanecio gigas* (Vatke) C.Jeffrey | Swellings | Yes | No |
| *Solanum indicum* L. | Nasal bleeding | Yes | No |
| *Solanum indicum* L. | Skin infection | Yes | No |
| *Solanum indicum* L. | Snake poison | Yes | No |
| *Sorghum bicolor* (L.) Moench. | Febrile illness | Yes | No |
| *Sorghum bicolor* (L.) Moench. | Respiratory organ infection | Yes | No |
| *Stephania abyssinica* (Quart.-Dill. & A.Rich.) Walp. | Cancer | Yes | No |
| *Strychnos spinosa* Lam. | Fire accident | Yes | No |
| *Strychnos spinosa* Lam. | Spiritual | Yes | No |
| *Strychnos spinosa* Lam. | Toothache | Yes | No |
| *Syzygium guineense* (Willd.) DC. | Breast cancer | Yes | No |
| *Syzygium guineense* (Willd.) DC. | Cancer | Yes | No |
| *Syzygium guineense* (Willd.) DC. | Pain relief | Yes | No |
| *Syzygium guineense* (Willd.) DC. | Swellings | Yes | No |
| *Thymus schimperi* Ronniger | Blood pressure | Yes | No |
| *Thymus schimperi* Ronniger | Spiritual | Yes | No |
| *Trigonella foenum-graecum* L. | Abnormal menstruation cycle | Yes | No |
| *Trigonella foenum-graecum* L. | Blood pressure | Yes | No |
| *Trigonella foenum-graecum* L. | Cough | Yes | No |
| *Trigonella foenum-graecum* L. | Gastric diseases | Yes | No |
| *Trigonella foenum-graecum* L. | Loss of apetite | Yes | No |
| *Trigonella foenum-graecum* L. | Lung infection | Yes | No |
| *Trigonella foenum-graecum* L. | Menstruation cycle disorder | Yes | No |
| *Trigonella foenum-graecum* L. | Stomachache | Yes | No |
| *Trigonella foenum-graecum* L. | Tuberculosis | Yes | No |
| *Trigonella foenum-graecum* L. | Weight loss | Yes | No |
| *Urtica dioica* L. | Cancer | Yes | No |
| *Urtica simensis* Hochst. ex A.Rich. | Amoeba | Yes | No |
| *Urtica simensis* Hochst. ex A.Rich. | Intestinal worms | Yes | No |
| *Urtica simensis* Hochst. ex A.Rich. | Stomachache | Yes | No |
| *Gymnanthemum amygdalinum* (Delile) Sch.Bip. | Typhoid | Yes | No |
| *Gymnanthemum auriculiferum* (Hiern) Isawumi | Bath of mother after giving a birth | Yes | No |
| *Gymnanthemum myrianthum* (Hook.f.) H.Rob. | Headache | Yes | No |
| *Gymnanthemum myrianthum* (Hook.f.) H.Rob. | Respiratory organ infection | Yes | No |
| *Vicia faba* L. | Gastric diseases | Yes | No |
| *Xanthium strumarium.* L. | Nerve case | Yes | No |
| *Zingiber officinale* Roscoe | Stomachache | Yes | No |
| *Zingiber officinale* Roscoe | Tung infection | Yes | No |
| *Vachellia oerfota* (Forssk.) Kyal. & Boatwr. | General health | No | Yes |
| *Vachellia oerfota* (Forssk.) Kyal. & Boatwr. | Spiritual | No | Yes |
| *Vachellia tortilis (*Forssk.) Galasso & Banfi | Intestinal worms | No | Yes |
| *Vachellia tortilis (*Forssk.) Galasso & Banfi | Malaria | No | Yes |
| *Vachellia tortilis (*Forssk.) Galasso & Banfi | Spiritual | No | Yes |
| *Achyranthes aspera* L. | Diarrhea | No | Yes |
| *Achyranthes aspera* L. | Skin infection | No | Yes |
| *Achyranthes aspera* L. | Spiritual | No | Yes |
| *Achyranthes aspera* L. | Stomachache | No | Yes |
| *Acokanthera schimperi* (A.DC.) Benth. & Hook.f. ex Schweinf. | Skin infection | No | Yes |
| *Acokanthera schimperi* (A.DC.) Benth. & Hook.f. ex Schweinf. | Wound | No | Yes |
| *Albizia gummifera* (J.F.Gmel.) C.A.Sm. | Epilepsy | No | Yes |
| *Allium sativum* L. | Febrile illness | No | Yes |
| *Allium sativum* L. | Fever | No | Yes |
| *Allium sativum* L. | Tonsillitis | No | Yes |
| *Aloe macrocarpa* Tod. | Breast cancer | No | Yes |
| *Aloe macrocarpa* Tod. | Cancer | No | Yes |
| *Aloe macrocarpa* Tod. | Diarrhea | No | Yes |
| *Aloe macrocarpa* Tod. | Fever | No | Yes |
| *Aloe macrocarpa* Tod. | Intestinal worms | No | Yes |
| *Aloe macrocarpa* Tod. | Jaundice | No | Yes |
| *Aloe macrocarpa* Tod. | Lung infection | No | Yes |
| *Aloe macrocarpa* Tod. | Malaria | No | Yes |
| *Aloe macrocarpa* Tod. | Pain relief | No | Yes |
| *Aloe macrocarpa* Tod. | Stomachache | No | Yes |
| *Aloe macrocarpa* Tod. | Typhoid | No | Yes |
| *Aloe macrocarpa* Tod. | Urinary organ infection | No | Yes |
| *Aloe macrocarpa* Tod. | Wound | No | Yes |
| *Aloe pirottae* A.Berger | Gastric diseases | No | Yes |
| *Aloe pirottae* A.Berger | Jaundice | No | Yes |
| *Aloe pirottae* A.Berger | Kidney infection | No | Yes |
| *Aloe pirottae* A.Berger | Menstruation cycle disorder | No | Yes |
| *Aloe pirottae* A.Berger | Vaginal infection | No | Yes |
| *Aloe vera* (L.) Burm.f. | Stomachache | No | Yes |
| *Argemone mexicana* L. | Blood pressure | No | Yes |
| *Argemone mexicana* L. | Cancer | No | Yes |
| *Argemone mexicana* L. | Jaundice | No | Yes |
| *Argemone mexicana* L. | Wound | No | Yes |
| *Artemisia abyssinica* Sch.Bip. ex A.Rich. | Blood pressure | No | Yes |
| *Artemisia abyssinica* Sch.Bip. ex A.Rich. | Malaria | No | Yes |
| *Artemisia abyssinica* Sch.Bip. ex A.Rich. | Nasal bleeding | No | Yes |
| *Asparagus africanus* Lam*.* | Ear infection | No | Yes |
| *Asparagus africanus* Lam*.* | Rabies | No | Yes |
| *Balanites aegyptiaca* (L.) Delile | Headache | No | Yes |
| *Balanites aegyptiaca* (L.) Delile | Mental case | No | Yes |
| *Balanites aegyptiaca* (L.) Delile | Cancer | No | Yes |
| *Beta vulgaris* L. | Anemia | No | Yes |
| *Beta vulgaris* L. | Wound | No | Yes |
| *Brassica carinata* A.Braun | Constipation | No | Yes |
| *Brassica carinata* A.Braun | Fever | No | Yes |
| *Brassica carinata* A.Braun | Skin infection | No | Yes |
| *Brassica carinata* A.Braun | Toothache | No | Yes |
| *Calendula officinalis* L. | Amoeba | No | Yes |
| *Calpurnia aurea* (Aiton) Benth. | Circumcision wound | No | Yes |
| *Calpurnia aurea* (Aiton) Benth. | Fever | No | Yes |
| *Calpurnia aurea* (Aiton) Benth. | Skin infection | No | Yes |
| *Calpurnia aurea* (Aiton) Benth. | Stomachache | No | Yes |
| *Calpurnia aurea* (Aiton) Benth. | Toothache | No | Yes |
| *Calpurnia aurea* (Aiton) Benth. | Typhoid | No | Yes |
| *Capsella bursa-pastoris* Medik. | Asthma | No | Yes |
| *Capsella bursa-pastoris* Medik. | Cough | No | Yes |
| *Capsella bursa-pastoris* Medik. | Lung infection | No | Yes |
| *Carica papaya* L. | Bath of mother after giving a birth | No | Yes |
| *Carica papaya* L. | Blood pressure | No | Yes |
| *Carica papaya* L. | Intestinal worms | No | Yes |
| *Carissa spinarum* L. | Febrile illness | No | Yes |
| *Carissa spinarum* L. | Headache | No | Yes |
| *Carissa spinarum* L. | Spiritual | No | Yes |
| *Casimiroa edulis* La Llave | Gastric diseases | No | Yes |
| *Casuarina equisetifolia* L. | Rabies | No | Yes |
| *Catha edulis* (Vahl) Forssk. ex Endl. | Skin infection | No | Yes |
| *Citrus × aurantiifolia* (Christm.) Swingle | Blood pressure | No | Yes |
| *Citrus limon* (L.) Osbeck | Amoeba | No | Yes |
| *Citrus limon* (L.) Osbeck | Blood pressure | No | Yes |
| *Citrus limon* (L.) Osbeck | Common cold | No | Yes |
| *Citrus limon* (L.) Osbeck | Fever | No | Yes |
| *Citrus limon* (L.) Osbeck | Stomachache | No | Yes |
| *Clematis* hirsuta Perr. & Guill. | Cancer | No | Yes |
| *Clematis* hirsuta Perr. & Guill. | Wound | No | Yes |
| *Rotheca myricoides* (Hochst.) Steane & Mabb. | Rabies | No | Yes |
| *Rotheca myricoides* (Hochst.) Steane & Mabb. | Spiritual | No | Yes |
| *Rotheca myricoides* (Hochst.) Steane & Mabb. | Stomachache | No | Yes |
| *Clutia abyssinica* Jaub. & Spach | Bone cancer | No | Yes |
| *Clutia abyssinica* Jaub. & Spach | Snake poison | No | Yes |
| *Clutia abyssinica* Jaub. & Spach | Spiritual | No | Yes |
| *Clutia abyssinica* Jaub. & Spach | Swellings | No | Yes |
| *Clutia abyssinica* Jaub. & Spach | Wound | No | Yes |
| *Coffea arabica* L. | Cancer | No | Yes |
| *Coffea arabica* L. | Gastric diseases | No | Yes |
| *Coffea arabica* L. | Kidney infection | No | Yes |
| *Commelina benghalensis* L. | Swellings | No | Yes |
| *Cordia africana* Lam. | Blood pressure | No | Yes |
| *Cordia africana* Lam. | Diarrhea | No | Yes |
| *Cordia africana* Lam. | Spiritual | No | Yes |
| *Croton macrostachyus* Hochst. ex Delile | Abortion | No | Yes |
| *Croton macrostachyus* Hochst. ex Delile | Asthma | No | Yes |
| *Croton macrostachyus* Hochst. ex Delile | Circumcision wound | No | Yes |
| *Croton macrostachyus* Hochst. ex Delile | Giardia | No | Yes |
| *Croton macrostachyus* Hochst. ex Delile | Jaundice | No | Yes |
| *Croton macrostachyus* Hochst. ex Delile | Lung infection | No | Yes |
| *Croton macrostachyus* Hochst. ex Delile | Placental delay during birth | No | Yes |
| *Croton macrostachyus* Hochst. ex Delile | Tetanus | No | Yes |
| *Cyathula polycephala* Baker | Febrile illness | No | Yes |
| *Datura stramonium* L. test | Skin infection | No | Yes |
| *Datura stramonium* L. test | Toothache | No | Yes |
| *Daucus carota* L. | Jaundice | No | Yes |
| *Daucus carota* L. | Passive sexual interest | No | Yes |
| *Dodonaea viscosa subsp. angustifolia* (L.f.) J.G.West | Bone injury | No | Yes |
| *Dodonaea viscosa subsp. angustifolia* (L.f.) J.G.West | Circumcision wound | No | Yes |
| *Dodonaea viscosa subsp. angustifolia* (L.f.) J.G.West | Gastric diseases | No | Yes |
| *Dodonaea viscosa subsp. angustifolia* (L.f.) J.G.West | Headache | No | Yes |
| *Dodonaea viscosa subsp. angustifolia* (L.f.) J.G.West | Lung infection | No | Yes |
| *Dodonaea viscosa subsp. angustifolia* (L.f.) J.G.West | Stomachache | No | Yes |
| *Ehretia cymosa* Thonn. | Stomachache | No | Yes |
| *Ekebergia capensis* Sparrm. | Bone cancer | No | Yes |
| *Ekebergia capensis* Sparrm. | Cancer | No | Yes |
| *Ekebergia capensis* Sparrm. | Fever | No | Yes |
| *Ekebergia capensis* Sparrm. | Glandular | No | Yes |
| *Ekebergia capensis* Sparrm. | Placental delay during birth | No | Yes |
| *Ekebergia capensis* Sparrm. | Skin infection | No | Yes |
| *Ekebergia capensis* Sparrm. | Spiritual | No | Yes |
| *Ekebergia capensis* Sparrm. | Wound | No | Yes |
| *Eleusine coracana* (L.) Gaertn. | Bone injury | No | Yes |
| *Eleusine coracana* (L.) Gaertn. | Wound | No | Yes |
| *Eragrostis tef* (Zuccagni) Trotter | Bone injury | No | Yes |
| *Eragrostis tef* (Zuccagni) Trotter | Wound | No | Yes |
| *Erica arborea* L. | Malaria | No | Yes |
| *Erica arborea* L. | Spiritual | No | Yes |
| *Erica arborea* L. | Wound | No | Yes |
| *Erythrina abyssinica* Lam. | Diarrhea | No | Yes |
| *Erythrina abyssinica* Lam. | Goiter | No | Yes |
| *Erythrina abyssinica* Lam. | Intestinal worms | No | Yes |
| *Erythrina abyssinica* Lam. | Rabies | No | Yes |
| *Erythrina abyssinica* Lam. | Spiritual | No | Yes |
| *Corymbia citriodora* (Hook.) K.D.Hill & L.A.S.Johnson | Gonorrhea | No | Yes |
| *Eucalyptus globulus* Labill. | Headache | No | Yes |
| *Euclea divinorum* Hiern | Circumcision wound | No | Yes |
| *Euclea divinorum* Hiern | Intestinal worms | No | Yes |
| *Euclea divinorum* Hiern | Skin infection | No | Yes |
| *Euclea divinorum* Hiern | Weight loss | No | Yes |
| *Euphorbia ampliphylla* Pax | Cancer | No | Yes |
| *Euphorbia ampliphylla* Pax | Epilepsy | No | Yes |
| *Ficus sycomorus* L. | Glandular | No | Yes |
| *Ficus sycomorus* L. | Tonsillitis | No | Yes |
| *Grewia ferruginea* Hochst. ex A.Rich. | Respiratory organ infection | No | Yes |
| *Hagenia abyssinica* (Bruce) J.F.Gmel. | Amoeba | No | Yes |
| *Hagenia abyssinica* (Bruce) J.F.Gmel. | Diarrhea | No | Yes |
| *Hagenia abyssinica* (Bruce) J.F.Gmel. | Febrile illness | No | Yes |
| *Hagenia abyssinica* (Bruce) J.F.Gmel. | Gonorrhea | No | Yes |
| *Hagenia abyssinica* (Bruce) J.F.Gmel. | Tapeworm | No | Yes |
| *Helianthus annuus* L*.* | Febrile illness | No | Yes |
| *Helianthus annuus* L*.* | Tung infection | No | Yes |
| *Hordeum vulgare* L. | Bone injury | No | Yes |
| *Hordeum vulgare* L. | Wound | No | Yes |
| *Indigofera arrecta* Hochst. ex A.Rich. | General health | No | Yes |
| *Justicia schimperiana* (Hochst. ex Nees) T.Anderson | Ear infection | No | Yes |
| *Kalanchoe densiflora* Rolfe | Muscular/joint pain | No | Yes |
| *Kniphofia foliosa* Hochst. | Stomachache | No | Yes |
| *Lactuca inermis* Forssk. | Febrile illness | No | Yes |
| *Lagenaria siceraria* (Molina) Standl. | Fever | No | Yes |
| *Lagenaria siceraria* (Molina) Standl. | Joint pain | No | Yes |
| *Lagenaria siceraria* (Molina) Standl. | Lung infection | No | Yes |
| *Lantana camara* L. | Sneezing | No | Yes |
| *Lepidium sativum* L. | Common cold | No | Yes |
| *Lepidium sativum* L. | Dry skin treatment | No | Yes |
| *Lepidium sativum* L. | Febrile illness | No | Yes |
| *Lepidium sativum* L. | Malaria | No | Yes |
| *Lepidium sativum* L. | Vaginal infection | No | Yes |
| *Lippia javanica* (Burm.f.) Spreng. | Blood pressure | No | Yes |
| *Lippia javanica* (Burm.f.) Spreng. | Diarrhea | No | Yes |
| *Lippia javanica* (Burm.f.) Spreng. | Stomachache | No | Yes |
| *Maesa lanceolata* Forssk. | Muscular/joint pain | No | Yes |
| *Maesa lanceolata* Forssk. | Nerve case | No | Yes |
| *Gymnosporia senegalensis* (Lam.) Loes. | Jaundice | No | Yes |
| *Gymnosporia senegalensis* (Lam.) Loes. | Malaria | No | Yes |
| *Gymnosporia senegalensis* (Lam.) Loes. | Skin infection | No | Yes |
| *Melia azedarach* L. | Cancer | No | Yes |
| *Melia azedarach* L. | Fever | No | Yes |
| *Melia azedarach* L. | Glandular | No | Yes |
| *Melia azedarach* L. | Intestinal worms | No | Yes |
| *Melia azedarach* L. | Jaundice | No | Yes |
| *Melia azedarach* L. | Toothache | No | Yes |
| *Melia azedarach* L. | Typhoid | No | Yes |
| *Mentha spicata* L. | Blood pressure | No | Yes |
| *Millettia ferruginea* (Hochst.) Hochst. ex Baker | Blood pressure | No | Yes |
| *Millettia ferruginea* (Hochst.) Hochst. ex Baker | Gonorrhea | No | Yes |
| *Millettia ferruginea* (Hochst.) Hochst. ex Baker | Stomachache | No | Yes |
| *Mimusops kummel* Bruce ex A.DC. | Diarrhea | No | Yes |
| *Mimusops kummel* Bruce ex A.DC. | Lung infection | No | Yes |
| *Moringa stenopetala* (Baker f.) Cufod. | Cholesterol | No | Yes |
| *Moringa stenopetala* (Baker f.) Cufod. | Diarrhea | No | Yes |
| *Moringa stenopetala* (Baker f.) Cufod. | Gastric diseases | No | Yes |
| *Moringa stenopetala* (Baker f.) Cufod. | Intestinal worms | No | Yes |
| *Moringa stenopetala* (Baker f.) Cufod. | Lung infection | No | Yes |
| *Moringa stenopetala* (Baker f.) Cufod. | Malaria | No | Yes |
| *Moringa stenopetala* (Baker f.) Cufod. | Nerve case | No | Yes |
| *Moringa stenopetala* (Baker f.) Cufod. | Pain relief | No | Yes |
| *Moringa stenopetala* (Baker f.) Cufod. | Typhoid | No | Yes |
| *Myrica salicifolia* Hochst. ex A.Rich. | Spiritual | No | Yes |
| *Nicotiana tabacum* L. | Common cold | No | Yes |
| *Nigella sativa* L. | Amoeba | No | Yes |
| *Nigella sativa* L. | Common cold | No | Yes |
| *Nigella sativa* L. | Febrile illness | No | Yes |
| *Nigella sativa* L. | Malaria | No | Yes |
| *Nigella sativa* L. | Nerve case | No | Yes |
| *Nigella sativa* L. | Pain relief | No | Yes |
| *Nuxia congesta* R.Br. ex Fresen. | Cancer | No | Yes |
| *Ocimum jamesii* Sebald | Febrile illness | No | Yes |
| *Ocimum jamesii* Sebald | Worms | No | Yes |
| *Ocimum lamiifolium* Hochst. ex Benth. | Fever | No | Yes |
| *Ocimum lamiifolium* Hochst. ex Benth. | Stomachache | No | Yes |
| *Ocimum gratissimum* L. | Eye infection | No | Yes |
| *Ocimum gratissimum* L. | Vomiting | No | Yes |
| *Olea europaea subsp. cuspidata* (Wall. & G.Don) Cif. | Anemia | No | Yes |
| *Olea europaea subsp. cuspidata* (Wall. & G.Don) Cif. | Asthma | No | Yes |
| *Olea europaea subsp. cuspidata* (Wall. & G.Don) Cif. | Cough | No | Yes |
| *Olea europaea subsp. cuspidata* (Wall. & G.Don) Cif. | Intestinal worms | No | Yes |
| *Olea europaea subsp. cuspidata* (Wall. & G.Don) Cif. | Jaundice | No | Yes |
| *Olea europaea subsp. cuspidata* (Wall. & G.Don) Cif. | Kidney infection | No | Yes |
| *Olea europaea subsp. cuspidata* (Wall. & G.Don) Cif. | Malaria | No | Yes |
| *Olea europaea subsp. cuspidata* (Wall. & G.Don) Cif. | Pain relief | No | Yes |
| *Olea europaea subsp. cuspidata* (Wall. & G.Don) Cif. | Respiratory organ infection | No | Yes |
| *Olea europaea subsp. cuspidata* (Wall. & G.Don) Cif. | Spiritual | No | Yes |
| *Olea europaea subsp. cuspidata* (Wall. & G.Don) Cif. | Swellings | No | Yes |
| *Olea europaea subsp. cuspidata* (Wall. & G.Don) Cif. | Tuberculosis | No | Yes |
| *Olea europaea subsp. cuspidata* (Wall. & G.Don) Cif. | Wound | No | Yes |
| *Olinia rochetiana* A.Juss. | Cancer | No | Yes |
| *Olinia rochetiana* A.Juss. | Circumcision wound | No | Yes |
| *Olinia rochetiana* A.Juss. | Skin infection | No | Yes |
| *Olinia rochetiana* A.Juss. | Stomachache | No | Yes |
| *Olinia rochetiana* A.Juss. | Toothache | No | Yes |
| *Olinia rochetiana* A.Juss. | Tuberculosis | No | Yes |
| *Olinia rochetiana* A.Juss. | Wound | No | Yes |
| *Pavonia urens* Cav. | Spiritual | No | Yes |
| *Persea americana* Mill. | Amoeba | No | Yes |
| *Persea americana* Mill. | Blood pressure | No | Yes |
| *Persicaria senegalensis* (Meisn.) Soják | Jaundice | No | Yes |
| *Persicaria senegalensis* (Meisn.) Soják | Malaria | No | Yes |
| *Pittosporum abyssinicum* Delile | Intestinal worms | No | Yes |
| *Pittosporum viridiflorum* Sims | Fever | No | Yes |
| *Pittosporum viridiflorum* Sims | Rabies | No | Yes |
| *Pittosporum viridiflorum* Sims | Spiritual | No | Yes |
| *Plantago lanceolata* L. | Epilepsy | No | Yes |
| *Aningeria altissima* (A.Chev.) Aubrév. & Pellegr. | Swellings | No | Yes |
| *Prunus africana* (Hook.f.) Kalkman | Skin infection | No | Yes |
| *Psidium guajava* L. | Cancer | No | Yes |
| *Psidium guajava* L. | Intestinal worms | No | Yes |
| *Psidium guajava* L. | Typhoid | No | Yes |
| *Psydrax schimperianus* (A.Rich.) Bridson | Blood pressure | No | Yes |
| *Psydrax schimperianus* (A.Rich.) Bridson | Cancer | No | Yes |
| *Psydrax schimperianus* (A.Rich.) Bridson | Febrile illness | No | Yes |
| *Psydrax schimperianus* (A.Rich.) Bridson | Skin infection | No | Yes |
| *Psydrax schimperianus* (A.Rich.) Bridson | Wound | No | Yes |
| *Rhamnus prinoides* L'Hér. | Tonsillitis | No | Yes |
| *Searsia glutinosa* (Hochst. ex A.Rich.) Moffett | Glandular | No | Yes |
| *Searsia pyroides* (Burch.) Moffett | Autism | No | Yes |
| *Searsia pyroides* (Burch.) Moffett | Breast cancer | No | Yes |
| *Searsia pyroides* (Burch.) Moffett | Passive sexual interest | No | Yes |
| *Ricinus communis* L. | Lung infection | No | Yes |
| *Rubus apetalus* Poir. | Toothache | No | Yes |
| *Rubus steudneri* Schweinf. | Amoeba | No | Yes |
| *Rubus steudneri* Schweinf. | Diarrhea | No | Yes |
| *Rubus steudneri* Schweinf. | Febrile illness | No | Yes |
| *Rubus steudneri* Schweinf. | Headache | No | Yes |
| *Rubus steudneri* Schweinf. | Nasal bleeding | No | Yes |
| *Rubus steudneri* Schweinf. | Skin infection | No | Yes |
| *Rubus steudneri* Schweinf. | Stomachache | No | Yes |
| *Rubus steudneri* Schweinf. | Urinary organ infection | No | Yes |
| *Rumex abyssinicus* Jacq. | Cancer | No | Yes |
| *Ruta chalepensis* L. | Dry skin treatment | No | Yes |
| *Ruta chalepensis* L. | Giardia | No | Yes |
| *Ruta chalepensis* L. | Nerve case | No | Yes |
| *Ruta chalepensis* L. | Placental delay during birth | No | Yes |
| *Ruta chalepensis* L. | Tuberculosis | No | Yes |
| *Salvia nilotica* Juss. ex Jacq. | Heart case | No | Yes |
| *Schinus molle* L. | Jaundice | No | Yes |
| *Schinus molle* L. | Nasal bleeding | No | Yes |
| *Schinus molle* L. | Tonsillitis | No | Yes |
| *Schrebera alata* (Hochst.) Welw. | Cancer | No | Yes |
| *Schrebera alata* (Hochst.) Welw. | Swellings | No | Yes |
| *Senna auriculata* (L.) Roxb. | Constipation | No | Yes |
| *Senna auriculata* (L.) Roxb. | Skin infection | No | Yes |
| *Sida schimperiana* Hochst. ex A.Rich. | Gonorrhea | No | Yes |
| *Sida schimperiana* Hochst. ex A.Rich. | Lung infection | No | Yes |
| *Sida schimperiana* Hochst. ex A.Rich. | Swellings | No | Yes |
| *Solanum incanum* L. | Diarrhea | No | Yes |
| *Solanum incanum* L. | Nasal bleeding | No | Yes |
| *Solanum incanum* L. | Snake poison | No | Yes |
| *Solanum marginatum* L.f. | Acid injury | No | Yes |
| *Solanum marginatum* L.f. | Autism | No | Yes |
| *Solanum marginatum* L.f. | Febrile illness | No | Yes |
| *Solanum marginatum* L.f. | Nasal bleeding | No | Yes |
| *Solanum marginatum* L.f. | Snake poison | No | Yes |
| *Solanum marginatum* L.f. | Spiritual | No | Yes |
| *Stephania abyssinica* (Quart.-Dill. & A.Rich.) Walp. | Gonorrhea | No | Yes |
| *Stephania abyssinica* (Quart.-Dill. & A.Rich.) Walp. | Jaundice | No | Yes |
| *Stephania abyssinica* (Quart.-Dill. & A.Rich.) Walp. | Lung infection | No | Yes |
| *Syzygium guineense* (Willd.) DC. | Circumcision wound | No | Yes |
| *Syzygium guineense* (Willd.) DC. | Lung infection | No | Yes |
| *Syzygium guineense* (Willd.) DC. | Skin infection | No | Yes |
| *Syzygium guineense* (Willd.) DC. | Weight loss | No | Yes |
| *Taverniera abyssinica* A.Rich. | Fever | No | Yes |
| *Vepris nobilis* (Delile) Mziray | Blood pressure | No | Yes |
| *Vepris nobilis* (Delile) Mziray | Dry skin treatment | No | Yes |
| *Vepris nobilis* (Delile) Mziray | Ear infection | No | Yes |
| *Vepris nobilis* (Delile) Mziray | Eye infection | No | Yes |
| *Vepris nobilis* (Delile) Mziray | Skin infection | No | Yes |
| *Terminalia brownii* Fresen. | Common cold | No | Yes |
| *Terminalia brownii* Fresen. | Headache | No | Yes |
| *Trichilia dregeana* Sond. | Jaundice | No | Yes |
| *Urtica dioica* L. | Febrile illness | No | Yes |
| *Urtica simensis* Hochst. ex A.Rich. | Febrile illness | No | Yes |
| *Urtica simensis* Hochst. ex A.Rich. | Gastric diseases | No | Yes |
| *Gymnanthemum amygdalinum* (Delile) Sch.Bip. | Blood pressure | No | Yes |
| *Gymnanthemum amygdalinum* (Delile) Sch.Bip. | Febrile illness | No | Yes |
| *Gymnanthemum amygdalinum* (Delile) Sch.Bip. | Gastric diseases | No | Yes |
| *Gymnanthemum amygdalinum* (Delile) Sch.Bip. | Gonorrhea | No | Yes |
| *Gymnanthemum amygdalinum* (Delile) Sch.Bip. | Head skin infection | No | Yes |
| *Gymnanthemum amygdalinum* (Delile) Sch.Bip. | Jaundice | No | Yes |
| *Gymnanthemum amygdalinum* (Delile) Sch.Bip. | Lung infection | No | Yes |
| *Gymnanthemum amygdalinum* (Delile) Sch.Bip. | Vomiting | No | Yes |
| *Withania somnifera* (L.) Dunal | Asthma | No | Yes |
| *Withania somnifera* (L.) Dunal | Cough | No | Yes |
| *Withania somnifera* (L.) Dunal | Febrile illness | No | Yes |
| *Withania somnifera* (L.) Dunal | Skin infection | No | Yes |
| *Ximenia americana* L. | Intestinal worms | No | Yes |
| *Ximenia americana* L. | Stomachache | No | Yes |
| *Ximenia americana* L. | Swellings | No | Yes |
| *Ximenia americana* L. | Wound | No | Yes |
| *Zea mays* L. | Sneezing | No | Yes |
| *Zehneria scabra* (L.f.) Sond. | Cancer | No | Yes |
| *Zehneria scabra* (L.f.) Sond. | Gastric diseases | No | Yes |
| *Zehneria scabra* (L.f.) Sond. | Spiritual | No | Yes |
| *Zehneria scabra* (L.f.) Sond. | Swellings | No | Yes |
| *Zingiber officinale* Roscoe | Amoeba | No | Yes |
| *Zingiber officinale* Roscoe | Blood pressure | No | Yes |
| *Zingiber officinale* Roscoe | Constipation | No | Yes |
| *Zingiber officinale* Roscoe | Febrile illness | No | Yes |
| *Zingiber officinale* Roscoe | Fever | No | Yes |
| *Zingiber officinale* Roscoe | Gastric diseases | No | Yes |
| *Zingiber officinale* Roscoe | Passive sexual interest | No | Yes |
| *Zingiber officinale* Roscoe | Typhoid | No | Yes |
| *Ziziphus spina-christi* (L.) Willd. | Eye infection | No | Yes |
| *Ziziphus spina-christi* (L.) Willd. | Giardia | No | Yes |
| *Ziziphus spina-christi* (L.) Willd. | Gonorrhea | No | Yes |
| *Ziziphus spina-christi* (L.) Willd. | Intestinal worms | No | Yes |
| *Ziziphus spina-christi* (L.) Willd. | Rabies | No | Yes |
| *Ziziphus spina-christi* (L.) Willd. | Skin infection | No | Yes |
| *Ziziphus spina-christi* (L.) Willd. | Spiritual | No | Yes |
| *Ziziphus spina-christi* (L.) Willd. | Wound | No | Yes |
