# Supplementary material for: Ethnobotanical study on medicinal plant knowledge among three ethnic groups in peri-urban areas of south-central Ethiopia
Source: J Ethnobiol Ethnomed. 2023 Nov 23;19:55. doi: 10.1186/s13002-023-00629-w (PMC10668360; doi:10.1186/s13002-023-00629-w)
Supplement: Supplementary file 3 — Additional file 3. Rahman similarity index between Gedeo and Sidama ethnic groups. [file 13002_2023_629_MOESM3_ESM.docx]

Supplementary Table 3: Rahman similarity index between Gedeo and Sidama ethnic groups. ‘Yes’ indicates shared medicinal plants to treat the same ailments between the two ethnic groups. Whereas ‘Yes or No’ indicates a disparity between ethnic groups.

|  |  | **Ethnic groups** | |
| --- | --- | --- | --- |
| **Species** | **Ailment** | **Gedeo** | **Sidama** |
| *Acokanthera schimperi* (A.DC.) Benth. & Hook.f. ex Schweinf. | Spiritual | Yes | Yes |
| *Ajuga integrifolia* Buch.-Ham. ex D.Don | Stomachache | Yes | Yes |
| *Albizia gummifera* (J.F.Gmel.) C.A.Sm. | Amoeba | Yes | Yes |
| *Albizia gummifera* (J.F.Gmel.) C.A.Sm. | Cancer | Yes | Yes |
| *Albizia gummifera* (J.F.Gmel.) C.A.Sm. | Dizziness | Yes | Yes |
| *Albizia gummifera* (J.F.Gmel.) C.A.Sm. | Lung infection | Yes | Yes |
| *Albizia gummifera* (J.F.Gmel.) C.A.Sm. | Stomachache | Yes | Yes |
| *Albizia gummifera* (J.F.Gmel.) C.A.Sm. | Toothache | Yes | Yes |
| *Allium sativum* L. | Common cold | Yes | Yes |
| *Allium sativum* L. | Typhoid | Yes | Yes |
| *Artemisia abyssinica* Sch.Bip. ex A.Rich. | Spiritual | Yes | Yes |
| *Calpurnia aurea* (Aiton) Benth. | Intestinal worms | Yes | Yes |
| *Calpurnia aurea* (Aiton) Benth. | Jaundice | Yes | Yes |
| *Calpurnia aurea* (Aiton) Benth. | Spiritual | Yes | Yes |
| *Carica papaya* L. | Malaria | Yes | Yes |
| *Catha edulis* (Vahl) Forssk. ex Endl. | Depression | Yes | Yes |
| *Catha edulis* (Vahl) Forssk. ex Endl. | Gonorrhea | Yes | Yes |
| *Cinnamomum verum* J.Presl | Asthma | Yes | Yes |
| *Cinnamomum verum* J.Presl | Common cold | Yes | Yes |
| *Clutia abyssinica* Jaub. & Spach | Cancer | Yes | Yes |
| *Coffea arabica* L. | Wound | Yes | Yes |
| *Croton macrostachyus* Hochst. ex Delile | Amoeba | Yes | Yes |
| *Croton macrostachyus* Hochst. ex Delile | Cancer | Yes | Yes |
| *Croton macrostachyus* Hochst. ex Delile | Diarrhea | Yes | Yes |
| *Croton macrostachyus* Hochst. ex Delile | Dizziness | Yes | Yes |
| *Croton macrostachyus* Hochst. ex Delile | Eye infection | Yes | Yes |
| *Croton macrostachyus* Hochst. ex Delile | Febrile illness | Yes | Yes |
| *Croton macrostachyus* Hochst. ex Delile | Glandular | Yes | Yes |
| *Croton macrostachyus* Hochst. ex Delile | Gonorrhea | Yes | Yes |
| *Croton macrostachyus* Hochst. ex Delile | Lightning | Yes | Yes |
| *Croton macrostachyus* Hochst. ex Delile | Spiritual | Yes | Yes |
| *Croton macrostachyus* Hochst. ex Delile | Wound | Yes | Yes |
| *Cucurbita pepo* L. | Intestinal worms | Yes | Yes |
| *Datura stramonium* L. test | Head skin infection | Yes | Yes |
| *Ekebergia capensis* Sparrm. | Amoeba | Yes | Yes |
| *Ekebergia capensis* Sparrm. | Stomachache | Yes | Yes |
| *Ensete ventricosum* (Welw.) Cheesman | Lightning | Yes | Yes |
| *Eucalyptus globulus* Labill. | Asthma | Yes | Yes |
| *Eucalyptus globulus* Labill. | Bath of mother after giving a birth | Yes | Yes |
| *Eucalyptus globulus* Labill. | Common cold | Yes | Yes |
| *Galinsoga quadriradiata* Ruiz & Pav. | Tonsillitis | Yes | Yes |
| *Impatiens ethiopica* Grey-Wilson | Gonorrhea | Yes | Yes |
| *Lactuca inermis* Forssk. | Weight loss | Yes | Yes |
| *Lagenaria siceraria* (Molina) Standl. | Jaundice | Yes | Yes |
| *Melia azedarach* L. | Blood pressure | Yes | Yes |
| *Melia azedarach* L. | Depression | Yes | Yes |
| *Melia azedarach* L. | Diarrhea | Yes | Yes |
| *Melia azedarach* L. | Malaria | Yes | Yes |
| *Melia azedarach* L. | Stomachache | Yes | Yes |
| *Millettia ferruginea* (Hochst.) Hochst. ex Baker | Amoeba | Yes | Yes |
| *Millettia ferruginea* (Hochst.) Hochst. ex Baker | Skin infection | Yes | Yes |
| *Moringa stenopetala* (Baker f.) Cufod. | Blood pressure | Yes | Yes |
| *Moringa stenopetala* (Baker f.) Cufod. | Glandular | Yes | Yes |
| *Nicotiana tabacum* L. | Headache | Yes | Yes |
| *Nigella sativa* L. | Respiratory organ infection | Yes | Yes |
| *Ocimum lamiifolium* Hochst. ex Benth. | Febrile illness | Yes | Yes |
| *Olea europaea subsp. cuspidata* (Wall. & G.Don) Cif. | Breast cancer | Yes | Yes |
| *Olea europaea subsp. cuspidata* (Wall. & G.Don) Cif. | Skin infection | Yes | Yes |
| *Olinia rochetiana* A.Juss. | Glandular | Yes | Yes |
| *Phytolacca dodecandra* L'Hér. | Abortion | Yes | Yes |
| *Phytolacca dodecandra* L'Hér. | Giardia | Yes | Yes |
| *Phytolacca dodecandra* L'Hér. | Intestinal worms | Yes | Yes |
| *Phytolacca dodecandra* L'Hér. | Stomachache | Yes | Yes |
| *Afrocarpus falcatus* (Thunb.) C.N.Page | Gonorrhea | Yes | Yes |
| *Psidium guajava* L. | Blood pressure | Yes | Yes |
| *Psidium guajava* L. | Malaria | Yes | Yes |
| *Ricinus communis* L. | Swellings | Yes | Yes |
| *Ricinus communis* L. | Tonsillitis | Yes | Yes |
| *Ricinus communis* L. | Wound | Yes | Yes |
| *Ruta chalepensis* L. | Febrile illness | Yes | Yes |
| *Ruta chalepensis* L. | Gonorrhea | Yes | Yes |
| *Ruta chalepensis* L. | Typhoid | Yes | Yes |
| *Saccharum officinarum* L. | Gastric diseases | Yes | Yes |
| *Thymus schimperi* Ronniger | Blood pressure | Yes | Yes |
| *Trigonella foenum-graecum* L. | Blood pressure | Yes | Yes |
| *Gymnanthemum amygdalinum* (Delile) Sch.Bip. | Amoeba | Yes | Yes |
| *Gymnanthemum amygdalinum* (Delile) Sch.Bip. | Diarrhea | Yes | Yes |
| *Gymnanthemum amygdalinum* (Delile) Sch.Bip. | Malaria | Yes | Yes |
| *Gymnanthemum amygdalinum* (Delile) Sch.Bip. | Skin infection | Yes | Yes |
| *Gymnanthemum amygdalinum* (Delile) Sch.Bip. | Stomachache | Yes | Yes |
| *Vicia faba* L. | Gastric diseases | Yes | Yes |
| *Withania somnifera* (L.) Dunal | Spiritual | Yes | Yes |
| *Zingiber officinale* Roscoe | Common cold | Yes | Yes |
| *Zingiber officinale* Roscoe | Tonsillitis | Yes | Yes |
| *Zingiber officinale* Roscoe | Tung infection | Yes | Yes |
| *Zingiber officinale* Roscoe | Wound | Yes | Yes |
| *Achyranthes aspera* L. | Ear infection | Yes | No |
| *Achyranthes aspera* L. | Jaundice | Yes | No |
| *Achyranthes aspera* L. | Lung infection | Yes | No |
| *Achyranthes aspera* L. | Nerve case | Yes | No |
| *Achyranthes aspera* L. | Respiratory organ infection | Yes | No |
| *Albizia gummifera* (J.F.Gmel.) C.A.Sm. | Cough | Yes | No |
| *Albizia gummifera* (J.F.Gmel.) C.A.Sm. | Febrile illness | Yes | No |
| *Albizia gummifera* (J.F.Gmel.) C.A.Sm. | Fever | Yes | No |
| *Albizia gummifera* (J.F.Gmel.) C.A.Sm. | Fire accident | Yes | No |
| *Albizia gummifera* (J.F.Gmel.) C.A.Sm. | Glandular | Yes | No |
| *Albizia gummifera* (J.F.Gmel.) C.A.Sm. | Gonorrhea | Yes | No |
| *Albizia gummifera* (J.F.Gmel.) C.A.Sm. | Intestinal worms | Yes | No |
| *Albizia gummifera* (J.F.Gmel.) C.A.Sm. | Malaria | Yes | No |
| *Albizia gummifera* (J.F.Gmel.) C.A.Sm. | Menstruation cycle disorder | Yes | No |
| *Albizia gummifera* (J.F.Gmel.) C.A.Sm. | Skin infection | Yes | No |
| *Albizia gummifera* (J.F.Gmel.) C.A.Sm. | Spiritual | Yes | No |
| *Albizia gummifera* (J.F.Gmel.) C.A.Sm. | Swellings | Yes | No |
| *Albizia gummifera* (J.F.Gmel.) C.A.Sm. | Tuberculosis | Yes | No |
| *Albizia gummifera* (J.F.Gmel.) C.A.Sm. | Typhoid | Yes | No |
| *Allium cepa* L. | Nasal bleeding | Yes | No |
| *Allium cepa* L. | Passive sexual interest | Yes | No |
| *Allium cepa* L. | Weight loss | Yes | No |
| *Allium sativum* L. | Asthma | Yes | No |
| *Allium sativum* L. | Blood pressure | Yes | No |
| *Allium sativum* L. | Skin infection | Yes | No |
| *Allium sativum* L. | Stomachache | Yes | No |
| *Allium sativum* L. | Tung infection | Yes | No |
| *Aloe macrocarpa* Tod. | Gonorrhea | Yes | No |
| *Aloe pirottae* A.Berger | Ear infection | Yes | No |
| *Aloe pirottae* A.Berger | Passive sexual interest | Yes | No |
| *Ananas comosus* (L.) Merr. | Skin infection | Yes | No |
| *Artemisia abyssinica* Sch.Bip. ex A.Rich. | Bath of mother after giving a birth | Yes | No |
| *Artemisia abyssinica* Sch.Bip. ex A.Rich. | Chicken pox | Yes | No |
| *Artemisia abyssinica* Sch.Bip. ex A.Rich. | Febrile illness | Yes | No |
| *Artemisia abyssinica* Sch.Bip. ex A.Rich. | Headache | Yes | No |
| *Artemisia abyssinica* Sch.Bip. ex A.Rich. | Skin infection | Yes | No |
| *Oldeania alpina* (K.Schum.) Stapleton | Wound | Yes | No |
| *Arundo donax* L. | Swellings | Yes | No |
| *Asparagus africanus* Lam*.* | Breast cancer | Yes | No |
| *Asparagus africanus* Lam*.* | Cancer | Yes | No |
| *Asparagus africanus* Lam*.* | Epilepsy | Yes | No |
| *Asparagus africanus* Lam*.* | Jaundice | Yes | No |
| *Asparagus africanus* Lam*.* | Lung infection | Yes | No |
| *Asparagus africanus* Lam*.* | Skin infection | Yes | No |
| *Asparagus africanus* Lam*.* | Swellings | Yes | No |
| *Bersama abyssinica* Fresen. | Intestinal worms | Yes | No |
| *Bersama abyssinica* Fresen. | Lung infection | Yes | No |
| *Bersama abyssinica* Fresen. | Skin infection | Yes | No |
| *Bersama abyssinica* Fresen. | Stomachache | Yes | No |
| *Bidens macroptera* (Sch.Bip. ex Chiov.) Mesfin. | Abnormal menstruation cycle | Yes | No |
| *Bidens macroptera* (Sch.Bip. ex Chiov.) Mesfin. | Amoeba | Yes | No |
| *Bidens macroptera* (Sch.Bip. ex Chiov.) Mesfin. | Diarrhea | Yes | No |
| *Brassica carinata* A.Braun | Cough | Yes | No |
| *Brassica carinata* A.Braun | Lung infection | Yes | No |
| *Brucea antidysenterica* J.F.Mill. | Diarrhea | Yes | No |
| *Brucea antidysenterica* J.F.Mill. | Stomachache | Yes | No |
| *Calpurnia aurea* (Aiton) Benth. | Amoeba | Yes | No |
| *Calpurnia aurea* (Aiton) Benth. | Bone cancer | Yes | No |
| *Calpurnia aurea* (Aiton) Benth. | Breast cancer | Yes | No |
| *Calpurnia aurea* (Aiton) Benth. | Cancer | Yes | No |
| *Calpurnia aurea* (Aiton) Benth. | Febrile illness | Yes | No |
| *Calpurnia aurea* (Aiton) Benth. | Glandular | Yes | No |
| *Calpurnia aurea* (Aiton) Benth. | Headache | Yes | No |
| *Calpurnia aurea* (Aiton) Benth. | Respiratory organ infection | Yes | No |
| *Calpurnia aurea* (Aiton) Benth. | Swellings | Yes | No |
| *Calpurnia aurea* (Aiton) Benth. | Wound | Yes | No |
| *Capsicum annuum* L. | Anemia | Yes | No |
| *Capsicum annuum* L. | Common cold | Yes | No |
| *Capsicum annuum* L. | Tonsillitis | Yes | No |
| *Capsicum frutescens* L. | Amoeba | Yes | No |
| *Capsicum frutescens* L. | Intestinal worms | Yes | No |
| *Carica papaya* L. | Cancer | Yes | No |
| *Carica papaya* L. | Skin infection | Yes | No |
| *Carica papaya* L. | Typhoid | Yes | No |
| *Catha edulis* (Vahl) Forssk. ex Endl. | Diarrhea | Yes | No |
| *Celtis africana* Burm.f. | Asthma | Yes | No |
| *Celtis africana* Burm.f. | Diarrhea | Yes | No |
| *Celtis africana* Burm.f. | Giardia | Yes | No |
| *Celtis africana* Burm.f. | Glandular | Yes | No |
| *Celtis africana* Burm.f. | Headache | Yes | No |
| *Celtis africana* Burm.f. | Intestinal worms | Yes | No |
| *Celtis africana* Burm.f. | Jaundice | Yes | No |
| *Celtis africana* Burm.f. | Lung infection | Yes | No |
| *Celtis africana* Burm.f. | Skin infection | Yes | No |
| *Celtis africana* Burm.f. | Stomachache | Yes | No |
| *Celtis africana* Burm.f. | Wound | Yes | No |
| *Citrus × aurantiifolia* (Christm.) Swingle | Giardia | Yes | No |
| *Clausena anisata* (Willd.) Hook.f. ex Benth. | Swellings | Yes | No |
| *Clematis* hirsuta Perr. & Guill. | Breast cancer | Yes | No |
| *Clematis* hirsuta Perr. & Guill. | Ear infection | Yes | No |
| *Clutia abyssinica* Jaub. & Spach | Breast cancer | Yes | No |
| *Clutia abyssinica* Jaub. & Spach | Toothache | Yes | No |
| *Clutia lanceolata* Forssk*.* | Ear infection | Yes | No |
| *Coffea arabica* L. | Breast cancer | Yes | No |
| *Coffea arabica* L. | Depression | Yes | No |
| *Coffea arabica* L. | Gastric cancer | Yes | No |
| *Coffea arabica* L. | Jaundice | Yes | No |
| *Coffea arabica* L. | Sneezing | Yes | No |
| *Coffea arabica* L. | Swellings | Yes | No |
| *Coffea arabica* L. | Toothache | Yes | No |
| *Colocasia esculenta* (L.) Schott | Cancer | Yes | No |
| *Colocasia esculenta* (L.) Schott | Toothache | Yes | No |
| *Commelina benghalensis* L. | Amoeba | Yes | No |
| *Commelina benghalensis* L. | Skin infection | Yes | No |
| *Cordia africana* Lam. | Bone cancer | Yes | No |
| *Cordia africana* Lam. | Passive sexual interest | Yes | No |
| *Croton macrostachyus* Hochst. ex Delile | Allergy | Yes | No |
| *Croton macrostachyus* Hochst. ex Delile | Bone cancer | Yes | No |
| *Croton macrostachyus* Hochst. ex Delile | Breast cancer | Yes | No |
| *Croton macrostachyus* Hochst. ex Delile | Ear infection | Yes | No |
| *Croton macrostachyus* Hochst. ex Delile | Intestinal worms | Yes | No |
| *Croton macrostachyus* Hochst. ex Delile | Malaria | Yes | No |
| *Croton macrostachyus* Hochst. ex Delile | Menstruation cycle disorder | Yes | No |
| *Croton macrostachyus* Hochst. ex Delile | Skin infection | Yes | No |
| *Croton macrostachyus* Hochst. ex Delile | Stomachache | Yes | No |
| *Croton macrostachyus* Hochst. ex Delile | Typhoid | Yes | No |
| *Cucurbita pepo* L. | Amoeba | Yes | No |
| *Cymbopogon citratus* (DC.) Stapf | Abortion | Yes | No |
| *Cymbopogon citratus* (DC.) Stapf | Bath of mother after giving a birth | Yes | No |
| *Cymbopogon citratus* (DC.) Stapf | Blood pressure | Yes | No |
| *Cymbopogon citratus* (DC.) Stapf | Cancer | Yes | No |
| *Cymbopogon citratus* (DC.) Stapf | Cholesterol | Yes | No |
| *Cymbopogon citratus* (DC.) Stapf | Gonorrhea | Yes | No |
| *Cymbopogon citratus* (DC.) Stapf | Kidney infection | Yes | No |
| *Cymbopogon citratus* (DC.) Stapf | Stomachache | Yes | No |
| *Cymbopogon citratus* (DC.) Stapf | Vomiting | Yes | No |
| *Datura stramonium* L. test | Rabies | Yes | No |
| *Dalbergia lactea* Vatke | Amoeba | Yes | No |
| *Dalbergia lactea* Vatke | Gonorrhea | Yes | No |
| *Delonix elata* (L.) Gamble | Stomachache | Yes | No |
| *Drynaria volkensii* Heiron. | Cancer | Yes | No |
| *Drynaria volkensii* Heiron. | Ear infection | Yes | No |
| *Drynaria volkensii* Heiron. | Nasal bleeding | Yes | No |
| *Drynaria volkensii* Heiron. | Swellings | Yes | No |
| *Ehretia cymosa* Thonn. | Swellings | Yes | No |
| *Ekebergia capensis* Sparrm. | Diarrhea | Yes | No |
| *Ekebergia capensis* Sparrm. | Febrile illness | Yes | No |
| *Ekebergia capensis* Sparrm. | Swellings | Yes | No |
| *Embelia schimperi* Vatke | Glandular | Yes | No |
| *Embelia schimperi* Vatke | Gonorrhea | Yes | No |
| *Embelia schimperi* Vatke | Jaundice | Yes | No |
| *Ensete ventricosum* (Welw.) Cheesman | Amoeba | Yes | No |
| *Ensete ventricosum* (Welw.) Cheesman | Gastric diseases | Yes | No |
| *Ensete ventricosum* (Welw.) Cheesman | Swellings | Yes | No |
| *Erythrina abyssinica* Lam. | Cough | Yes | No |
| *Erythrina abyssinica* Lam. | Eye infection | Yes | No |
| *Erythrina abyssinica* Lam. | Fever | Yes | No |
| *Erythrina abyssinica* Lam. | Liver infection | Yes | No |
| *Erythrina abyssinica* Lam. | Lung infection | Yes | No |
| *Erythrina abyssinica* Lam. | Malaria | Yes | No |
| *Erythrina abyssinica* Lam. | Skin infection | Yes | No |
| *Erythrina abyssinica* Lam. | Tuberculosis | Yes | No |
| *Eucalyptus globulus* Labill. | Amoeba | Yes | No |
| *Eucalyptus globulus* Labill. | Nasal bleeding | Yes | No |
| *Euphorbia ampliphylla* Pax | Spiritual | Yes | No |
| *Euphorbia pulcherrima* Willd. ex Klotzsch | Fever | Yes | No |
| *Euphorbia tirucalli* L. | Cancer | Yes | No |
| *Fagaropsis angolensis* (Engl.) H.M.Gardner | Stomachache | Yes | No |
| *Fagaropsis angolensis* (Engl.) H.M.Gardner | Swellings | Yes | No |
| *Fagaropsis angolensis* (Engl.) H.M.Gardner | Wound | Yes | No |
| *Flacourtia indica* (Burm.f.) Merr. | Respiratory organ infection | Yes | No |
| *Flacourtia indica* (Burm.f.) Merr. | Snake poison | Yes | No |
| *Galinsoga quadriradiata* Ruiz & Pav. | Cancer | Yes | No |
| *Galinsoga quadriradiata* Ruiz & Pav. | Swellings | Yes | No |
| *Galinsoga quadriradiata* Ruiz & Pav. | Toothache | Yes | No |
| *Grewia ferruginea* Hochst. ex A.Rich. | Amoeba | Yes | No |
| *Grewia ferruginea* Hochst. ex A.Rich. | Cancer | Yes | No |
| *Grewia ferruginea* Hochst. ex A.Rich. | Epilepsy | Yes | No |
| *Grewia ferruginea* Hochst. ex A.Rich. | Febrile illness | Yes | No |
| *Grewia ferruginea* Hochst. ex A.Rich. | Headache | Yes | No |
| *Grewia ferruginea* Hochst. ex A.Rich. | Jaundice | Yes | No |
| *Grewia ferruginea* Hochst. ex A.Rich. | Swellings | Yes | No |
| *Grewia ferruginea* Hochst. ex A.Rich. | Wound | Yes | No |
| *Hagenia abyssinica* (Bruce) J.F.Gmel. | Intestinal worms | Yes | No |
| *Hibiscus macranthus* Hochst. ex A. Rich. | Fire accident | Yes | No |
| *Hordeum vulgare* L. | Lightning | Yes | No |
| *Hyparrhenia rufa* (Nees) Stapf | Cough | Yes | No |
| *Hyparrhenia rufa* (Nees) Stapf | Lung infection | Yes | No |
| *Hyparrhenia rufa* (Nees) Stapf | Swellings | Yes | No |
| *Juniperus procera* Hochst. ex Endl. | Respiratory organ infection | Yes | No |
| *Justicia schimperiana* (Hochst. ex Nees) T.Anderson | Epilepsy | Yes | No |
| *Justicia schimperiana* (Hochst. ex Nees) T.Anderson | Glandular | Yes | No |
| *Justicia schimperiana* (Hochst. ex Nees) T.Anderson | Goiter | Yes | No |
| *Justicia schimperiana* (Hochst. ex Nees) T.Anderson | Jaundice | Yes | No |
| *Justicia schimperiana* (Hochst. ex Nees) T.Anderson | Malaria | Yes | No |
| *Kalanchoe petitiana* A.Rich. | Bone injury | Yes | No |
| *Kanahia laniflora* (Forssk.) R.Br. | Jaundice | Yes | No |
| *Lactuca inermis* Forssk. | Anemia | Yes | No |
| *Lactuca inermis* Forssk. | Stomachache | Yes | No |
| *Lagenaria siceraria* (Molina) Standl. | Amoeba | Yes | No |
| *Lagenaria siceraria* (Molina) Standl. | Goiter | Yes | No |
| *Lagenaria siceraria* (Molina) Standl. | Pain relief | Yes | No |
| *Lepidium sativum* L. | Gastric diseases | Yes | No |
| *Leucas tomentosa* Gürke | Febrile illness | Yes | No |
| *Linum usitatissimum* L. | Cough | Yes | No |
| *Linum usitatissimum* L. | Lung infection | Yes | No |
| *Linum usitatissimum* L. | Tuberculosis | Yes | No |
| *Maesa lanceolata* Forssk. | Amoeba | Yes | No |
| *Maesa lanceolata* Forssk. | Cough | Yes | No |
| *Maesa lanceolata* Forssk. | Ear infection | Yes | No |
| *Maesa lanceolata* Forssk. | Gastric diseases | Yes | No |
| *Maesa lanceolata* Forssk. | Gonorrhea | Yes | No |
| *Maesa lanceolata* Forssk. | Jaundice | Yes | No |
| *Maesa lanceolata* Forssk. | Skin infection | Yes | No |
| *Melia azedarach* L. | Breast cancer | Yes | No |
| *Millettia ferruginea* (Hochst.) Hochst. ex Baker | Cancer | Yes | No |
| *Millettia ferruginea* (Hochst.) Hochst. ex Baker | Ear infection | Yes | No |
| *Millettia ferruginea* (Hochst.) Hochst. ex Baker | Goiter | Yes | No |
| *Millettia ferruginea* (Hochst.) Hochst. ex Baker | Jaundice | Yes | No |
| *Millettia ferruginea* (Hochst.) Hochst. ex Baker | Lung infection | Yes | No |
| *Millettia ferruginea* (Hochst.) Hochst. ex Baker | Malaria | Yes | No |
| *Millettia ferruginea* (Hochst.) Hochst. ex Baker | Pain relief | Yes | No |
| *Millettia ferruginea* (Hochst.) Hochst. ex Baker | Toothache | Yes | No |
| *Moringa stenopetala* (Baker f.) Cufod. | Cancer | Yes | No |
| *Moringa stenopetala* (Baker f.) Cufod. | Jaundice | Yes | No |
| *Moringa stenopetala* (Baker f.) Cufod. | Kidney infection | Yes | No |
| *Nigella sativa* L. | Asthma | Yes | No |
| *Nigella sativa* L. | Bone injury | Yes | No |
| *Nigella sativa* L. | Cancer | Yes | No |
| *Nigella sativa* L. | Fever | Yes | No |
| *Nigella sativa* L. | Nasal bleeding | Yes | No |
| *Nigella sativa* L. | Stomachache | Yes | No |
| *Nuxia congesta* R.Br. ex Fresen. | Breast cancer | Yes | No |
| *Nuxia congesta* R.Br. ex Fresen. | Skin infection | Yes | No |
| *Nuxia congesta* R.Br. ex Fresen. | Wound | Yes | No |
| *Ocimum lamiifolium* Hochst. ex Benth. | Amoeba | Yes | No |
| *Ocimum lamiifolium* Hochst. ex Benth. | Diarrhea | Yes | No |
| *Ocimum lamiifolium* Hochst. ex Benth. | Gonorrhea | Yes | No |
| *Ocimum lamiifolium* Hochst. ex Benth. | Muscular/joint pain | Yes | No |
| *Ocimum lamiifolium* Hochst. ex Benth. | Typhoid | Yes | No |
| *Ocimum gratissimum* L. | Amoeba | Yes | No |
| *Ocimum gratissimum* L. | Febrile illness | Yes | No |
| *Ocimum gratissimum* L. | Fever | Yes | No |
| *Ocimum gratissimum* L. | Kidney infection | Yes | No |
| *Ocimum gratissimum* L. | Malaria | Yes | No |
| *Ocimum gratissimum* L. | Stomachache | Yes | No |
| *Olea europaea subsp. cuspidata* (Wall. & G.Don) Cif. | Blood pressure | Yes | No |
| *Olea europaea subsp. cuspidata* (Wall. & G.Don) Cif. | Cancer | Yes | No |
| *Olea europaea subsp. cuspidata* (Wall. & G.Don) Cif. | Toothache | Yes | No |
| *Olea europaea subsp. cuspidata* (Wall. & G.Don) Cif. | Vaginal infection | Yes | No |
| *Phytolacca dodecandra* L'Hér. | Skin infection | Yes | No |
| *Coleus igniarius* Schweinf. | Intestinal worms | Yes | No |
| *Coleus igniarius* Schweinf. | Stomachache | Yes | No |
| *Afrocarpus falcatus* (Thunb.) C.N.Page | Amoeba | Yes | No |
| *Afrocarpus falcatus* (Thunb.) C.N.Page | Breast cancer | Yes | No |
| *Afrocarpus falcatus* (Thunb.) C.N.Page | Cancer | Yes | No |
| *Afrocarpus falcatus* (Thunb.) C.N.Page | Glandular | Yes | No |
| *Afrocarpus falcatus* (Thunb.) C.N.Page | Jaundice | Yes | No |
| *Afrocarpus falcatus* (Thunb.) C.N.Page | Malaria | Yes | No |
| *Afrocarpus falcatus* (Thunb.) C.N.Page | Toothache | Yes | No |
| *Afrocarpus falcatus* (Thunb.) C.N.Page | Typhoid | Yes | No |
| *Afrocarpus falcatus* (Thunb.) C.N.Page | Wound | Yes | No |
| *Prunus africana* (Hook.f.) Kalkman | Glandular | Yes | No |
| *Prunus africana* (Hook.f.) Kalkman | Goiter | Yes | No |
| *Psydrax schimperianus* (A.Rich.) Bridson | Muscle pain | Yes | No |
| *Psydrax schimperianus* (A.Rich.) Bridson | Muscular/joint pain | Yes | No |
| *Searsia pyroides* (Burch.) Moffett | Common cold | Yes | No |
| *Searsia pyroides* (Burch.) Moffett | Epilepsy | Yes | No |
| *Searsia pyroides* (Burch.) Moffett | Spiritual | Yes | No |
| *Ricinus communis* L. | Skin infection | Yes | No |
| *Solanum nigrum* L. | Stomachache | Yes | No |
| *Rubia cordifolia* L. | Malaria | Yes | No |
| *Rumex nepalensis* Spreng. | Intestinal worms | Yes | No |
| *Rumex nepalensis* Spreng. | Wound | Yes | No |
| *Ruta chalepensis* L. | Anemia | Yes | No |
| *Ruta chalepensis* L. | Asthma | Yes | No |
| *Ruta chalepensis* L. | Bath of mother after giving a birth | Yes | No |
| *Ruta chalepensis* L. | Breast cancer | Yes | No |
| *Ruta chalepensis* L. | Epilepsy | Yes | No |
| *Ruta chalepensis* L. | Glandular | Yes | No |
| *Ruta chalepensis* L. | Headache | Yes | No |
| *Ruta chalepensis* L. | Jaundice | Yes | No |
| *Ruta chalepensis* L. | Malaria | Yes | No |
| *Ruta chalepensis* L. | Menstruation cycle disorder | Yes | No |
| *Ruta chalepensis* L. | Nasal bleeding | Yes | No |
| *Ruta chalepensis* L. | Stomachache | Yes | No |
| *Ruta chalepensis* L. | Swellings | Yes | No |
| *Ruta chalepensis* L. | Vomiting | Yes | No |
| *Sesbania sesban* (L.) Merr. | Rabies | Yes | No |
| *Sesbania sesban* (L.) Merr. | Snake poison | Yes | No |
| *Sida ovata* Forssk. | Bone cancer | Yes | No |
| *Sida schimperiana* Hochst. ex A.Rich. | Fever | Yes | No |
| *Sida schimperiana* Hochst. ex A.Rich. | Glandular | Yes | No |
| *Sida schimperiana* Hochst. ex A.Rich. | Headache | Yes | No |
| *Sida schimperiana* Hochst. ex A.Rich. | Jaundice | Yes | No |
| *Sida schimperiana* Hochst. ex A.Rich. | Toothache | Yes | No |
| *Sida schimperiana* Hochst. ex A.Rich. | Wound | Yes | No |
| *Solanecio gigas* (Vatke) C.Jeffrey | Amoeba | Yes | No |
| *Solanecio gigas* (Vatke) C.Jeffrey | Diarrhea | Yes | No |
| *Solanecio gigas* (Vatke) C.Jeffrey | Fever | Yes | No |
| *Solanecio gigas* (Vatke) C.Jeffrey | Gastric diseases | Yes | No |
| *Solanecio gigas* (Vatke) C.Jeffrey | Glandular | Yes | No |
| *Solanecio gigas* (Vatke) C.Jeffrey | Jaundice | Yes | No |
| *Solanecio gigas* (Vatke) C.Jeffrey | Lung infection | Yes | No |
| *Solanecio gigas* (Vatke) C.Jeffrey | Malaria | Yes | No |
| *Solanecio gigas* (Vatke) C.Jeffrey | Nasal bleeding | Yes | No |
| *Solanecio gigas* (Vatke) C.Jeffrey | Swellings | Yes | No |
| *Solanum indicum* L. | Nasal bleeding | Yes | No |
| *Solanum indicum* L. | Skin infection | Yes | No |
| *Solanum indicum* L. | Snake poison | Yes | No |
| *Sorghum bicolor* (L.) Moench. | Febrile illness | Yes | No |
| *Sorghum bicolor* (L.) Moench. | Respiratory organ infection | Yes | No |
| *Stephania abyssinica* (Quart.-Dill. & A.Rich.) Walp. | Cancer | Yes | No |
| *Strychnos spinosa* Lam. | Fire accident | Yes | No |
| *Strychnos spinosa* Lam. | Spiritual | Yes | No |
| *Strychnos spinosa* Lam. | Toothache | Yes | No |
| *Syzygium guineense* (Willd.) DC. | Breast cancer | Yes | No |
| *Syzygium guineense* (Willd.) DC. | Cancer | Yes | No |
| *Syzygium guineense* (Willd.) DC. | Glandular | Yes | No |
| *Syzygium guineense* (Willd.) DC. | Pain relief | Yes | No |
| *Syzygium guineense* (Willd.) DC. | Swellings | Yes | No |
| *Thymus schimperi* Ronniger | Spiritual | Yes | No |
| *Trigonella foenum-graecum* L. | Abnormal menstruation cycle | Yes | No |
| *Trigonella foenum-graecum* L. | Cough | Yes | No |
| *Trigonella foenum-graecum* L. | Gastric diseases | Yes | No |
| *Trigonella foenum-graecum* L. | Loss of apetite | Yes | No |
| *Trigonella foenum-graecum* L. | Lung infection | Yes | No |
| *Trigonella foenum-graecum* L. | Menstruation cycle disorder | Yes | No |
| *Trigonella foenum-graecum* L. | Stomachache | Yes | No |
| *Trigonella foenum-graecum* L. | Tuberculosis | Yes | No |
| *Trigonella foenum-graecum* L. | Weight loss | Yes | No |
| *Urtica dioica* L. | Cancer | Yes | No |
| *Urtica simensis* Hochst. ex A.Rich. | Amoeba | Yes | No |
| *Urtica simensis* Hochst. ex A.Rich. | Intestinal worms | Yes | No |
| *Urtica simensis* Hochst. ex A.Rich. | Stomachache | Yes | No |
| *Gymnanthemum amygdalinum* (Delile) Sch.Bip. | Intestinal worms | Yes | No |
| *Gymnanthemum amygdalinum* (Delile) Sch.Bip. | Rabies | Yes | No |
| *Gymnanthemum amygdalinum* (Delile) Sch.Bip. | Typhoid | Yes | No |
| *Gymnanthemum auriculiferum* (Hiern) Isawumi | Bath of mother after giving a birth | Yes | No |
| *Gymnanthemum auriculiferum* (Hiern) Isawumi | Snake poison | Yes | No |
| *Gymnanthemum myrianthum* (Hook.f.) H.Rob. | Headache | Yes | No |
| *Gymnanthemum myrianthum* (Hook.f.) H.Rob. | Respiratory organ infection | Yes | No |
| *Xanthium strumarium.* L. | Nerve case | Yes | No |
| *Zingiber officinale* Roscoe | Stomachache | Yes | No |
| *Achyranthes aspera* L. | Cancer | No | Yes |
| *Achyranthes aspera* L. | Gonorrhea | No | Yes |
| *Achyranthes aspera* L. | Headache | No | Yes |
| *Achyranthes aspera* L. | Joint pain | No | Yes |
| *Achyranthes aspera* L. | Muscle pain | No | Yes |
| *Achyranthes aspera* L. | Stomachache | No | Yes |
| *Aframomum corrorima* (A.Braun) P.C.M.Jansen | Skin infection | No | Yes |
| *Aframomum corrorima* (A.Braun) P.C.M.Jansen | Tonsillitis | No | Yes |
| *Ajuga integrifolia* Buch.-Ham. ex D.Don | Anemia | No | Yes |
| *Ajuga integrifolia* Buch.-Ham. ex D.Don | Malaria | No | Yes |
| *Ajuga integrifolia* Buch.-Ham. ex D.Don | Pain relief | No | Yes |
| *Ajuga integrifolia* Buch.-Ham. ex D.Don | Weight loss | No | Yes |
| *Albizia gummifera* (J.F.Gmel.) C.A.Sm. | Goiter | No | Yes |
| *Albizia gummifera* (J.F.Gmel.) C.A.Sm. | Jaundice | No | Yes |
| *Allium sativum* L. | Chicken pox | No | Yes |
| *Allium sativum* L. | Febrile illness | No | Yes |
| *Allium sativum* L. | Fever | No | Yes |
| *Allium sativum* L. | Gonorrhea | No | Yes |
| *Allium sativum* L. | Headache | No | Yes |
| *Allium sativum* L. | Malaria | No | Yes |
| *Allium sativum* L. | Tonsillitis | No | Yes |
| *Aloe macrocarpa* Tod. | Malaria | No | Yes |
| *Aloe vera* (L.) Burm.f. | Amoeba | No | Yes |
| *Aloe vera* (L.) Burm.f. | Blood pressure | No | Yes |
| *Aloe vera* (L.) Burm.f. | Malaria | No | Yes |
| *Aloe vera* (L.) Burm.f. | Stomachache | No | Yes |
| *Antiaris toxicaria* (J.F.Gmel.) Lesch. | Rabies | No | Yes |
| *Artemisia absinthium* L. | Diabetes | No | Yes |
| *Artemisia absinthium* L. | Spiritual | No | Yes |
| *Balanites aegyptiaca* (L.) Delile | Amoeba | No | Yes |
| *Balanites aegyptiaca* (L.) Delile | Diarrhea | No | Yes |
| *Balanites aegyptiaca* (L.) Delile | Stomachache | No | Yes |
| *Bersama abyssinica* Fresen. | Amoeba | No | Yes |
| *Bersama abyssinica* Fresen. | Jaundice | No | Yes |
| *Bersama abyssinica* Fresen. | Spiritual | No | Yes |
| *Brucea antidysenterica* J.F.Mill. | Gonorrhea | No | Yes |
| *Calpurnia aurea* (Aiton) Benth. | Lung infection | No | Yes |
| *Calpurnia aurea* (Aiton) Benth. | Typhoid | No | Yes |
| *Capsicum annuum* L. | Intestinal worms | No | Yes |
| *Carica papaya* L. | Fever | No | Yes |
| *Carica papaya* L. | Gastric diseases | No | Yes |
| *Carica papaya* L. | Intestinal worms | No | Yes |
| *Carissa spinarum* L. | Diarrhea | No | Yes |
| *Carissa spinarum* L. | Gonorrhea | No | Yes |
| *Catha edulis* (Vahl) Forssk. ex Endl. | Amoeba | No | Yes |
| *Catha edulis* (Vahl) Forssk. ex Endl. | Spiritual | No | Yes |
| *Cinnamomum verum* J.Presl | Fever | No | Yes |
| *Citrus × aurantiifolia* (Christm.) Swingle | Amoeba | No | Yes |
| *Citrus × aurantiifolia* (Christm.) Swingle | Anemia | No | Yes |
| *Clematis* hirsuta Perr. & Guill. | Jaundice | No | Yes |
| *Clutia abyssinica* Jaub. & Spach | Diarrhea | No | Yes |
| *Clutia abyssinica* Jaub. & Spach | Spiritual | No | Yes |
| *Clutia abyssinica* Jaub. & Spach | Swellings | No | Yes |
| *Coffea arabica* L. | Gastric diseases | No | Yes |
| *Coffea arabica* L. | Malaria | No | Yes |
| *Commelina africana* L. | Skin infection | No | Yes |
| *Coriandrum sativum* L. | Overall health | No | Yes |
| *Cordia africana* Lam. | Nerve case | No | Yes |
| *Croton macrostachyus* Hochst. ex Delile | Giardia | No | Yes |
| *Croton macrostachyus* Hochst. ex Delile | Jaundice | No | Yes |
| *Croton macrostachyus* Hochst. ex Delile | Lung infection | No | Yes |
| *Croton macrostachyus* Hochst. ex Delile | Tetanus | No | Yes |
| *Cucumis dipsaceus* Ehrenb. ex Spach | Jaundice | No | Yes |
| *Cucumis prophetarum* L. | Amoeba | No | Yes |
| *Cucumis prophetarum* L. | Balanced diet | No | Yes |
| *Cucumis prophetarum* L. | Cancer | No | Yes |
| *Cucumis prophetarum* L. | Diarrhea | No | Yes |
| *Cucumis prophetarum* L. | Glandular | No | Yes |
| *Cucumis prophetarum* L. | Jaundice | No | Yes |
| *Cucumis prophetarum* L. | Lung infection | No | Yes |
| *Cucumis prophetarum* L. | Respiratory organ infection | No | Yes |
| *Cucumis prophetarum* L. | Rheumatic | No | Yes |
| *Cucurbita pepo* L. | Tapeworm | No | Yes |
| *Cynodon dactylon* (L.) Pers. | Swellings | No | Yes |
| *Cynoglossum coeruleum* Hochst. ex A.DC. | Lung infection | No | Yes |
| *Cynoglossum coeruleum* Hochst. ex A.DC. | Skin infection | No | Yes |
| *Datura stramonium* L. test | Skin infection | No | Yes |
| *Dodonaea viscosa subsp. angustifolia* (L.f.) J.G.West | Lung infection | No | Yes |
| *Dovyalis caffra* (Hook.f. & Harv.) Warb. | Snake poison | No | Yes |
| *Echinops kebericho* Mesfin | Common cold | No | Yes |
| *Echinops kebericho* Mesfin | Febrile illness | No | Yes |
| *Echinops kebericho* Mesfin | Fever | No | Yes |
| *Echinops kebericho* Mesfin | Headache | No | Yes |
| *Ehretia cymosa* Thonn. | Cancer | No | Yes |
| *Ehretia cymosa* Thonn. | Lung infection | No | Yes |
| *Ehretia cymosa* Thonn. | Nasal bleeding | No | Yes |
| *Ehretia cymosa* Thonn. | Skin infection | No | Yes |
| *Ehretia cymosa* Thonn. | Wound | No | Yes |
| *Ekebergia capensis* Sparrm. | Fever | No | Yes |
| *Ekebergia capensis* Sparrm. | Goiter | No | Yes |
| *Ekebergia capensis* Sparrm. | Gonorrhea | No | Yes |
| *Ekebergia capensis* Sparrm. | Jaundice | No | Yes |
| *Ekebergia capensis* Sparrm. | Tuberculosis | No | Yes |
| *Ekebergia capensis* Sparrm. | Typhoid | No | Yes |
| *Erythrina abyssinica* Lam. | Toothache | No | Yes |
| *Eucalyptus globulus* Labill. | Dry skin treatment | No | Yes |
| *Eucalyptus globulus* Labill. | Fever | No | Yes |
| *Eucalyptus globulus* Labill. | Headache | No | Yes |
| *Eucalyptus globulus* Labill. | Mental case | No | Yes |
| *Eucalyptus globulus* Labill. | Nerve case | No | Yes |
| *Eucalyptus globulus* Labill. | Pain relief | No | Yes |
| *Eucalyptus globulus* Labill. | Skin infection | No | Yes |
| *Eucalyptus globulus* Labill. | Spiritual | No | Yes |
| *Euclea divinorum* Hiern | Intestinal worms | No | Yes |
| *Euclea racemosa subsp. schimperi* (A.DC.) F.White | Stomachache | No | Yes |
| *Galinsoga quadriradiata* Ruiz & Pav. | Goiter | No | Yes |
| *Justicia schimperiana* (Hochst. ex Nees) T.Anderson | Amoeba | No | Yes |
| *Justicia schimperiana* (Hochst. ex Nees) T.Anderson | Gonorrhea | No | Yes |
| *Justicia schimperiana* (Hochst. ex Nees) T.Anderson | Rabies | No | Yes |
| *Justicia schimperiana* (Hochst. ex Nees) T.Anderson | Sneezing | No | Yes |
| *Justicia schimperiana* (Hochst. ex Nees) T.Anderson | Stomachache | No | Yes |
| *Kalanchoe petitiana* A.Rich. | Broken bone | No | Yes |
| *Kalanchoe petitiana* A.Rich. | Diarrhea | No | Yes |
| *Kalanchoe petitiana* A.Rich. | Glandular | No | Yes |
| *Kalanchoe petitiana* A.Rich. | Muscular/joint pain | No | Yes |
| *Kalanchoe petitiana* A.Rich. | Pain relief | No | Yes |
| *Lactuca inermis* Forssk. | Balanced diet | No | Yes |
| *Lagenaria siceraria* (Molina) Standl. | Glandular | No | Yes |
| *Lagenaria siceraria* (Molina) Standl. | Lung infection | No | Yes |
| *Vicia lens* (L.) Coss. & Germ. | Chickenpox | No | Yes |
| *Vicia lens* (L.) Coss. & Germ. | Spider poison | No | Yes |
| *Vicia lens* (L.) Coss. & Germ. | Wound | No | Yes |
| *Linum usitatissimum* L. | Blood pressure | No | Yes |
| *Linum usitatissimum* L. | Diabetes | No | Yes |
| *Linum usitatissimum* L. | Gastric diseases | No | Yes |
| *Linum usitatissimum* L. | Kidney infection | No | Yes |
| *Linum usitatissimum* L. | Weight loss | No | Yes |
| *Lippia javanica* (Burm.f.) Spreng. | Blood pressure | No | Yes |
| *Melia azedarach* L. | Diabetes | No | Yes |
| *Melia azedarach* L. | Gastric diseases | No | Yes |
| *Melia azedarach* L. | Nasal bleeding | No | Yes |
| *Melia azedarach* L. | Pain relief | No | Yes |
| *Mentha spicata* L. | Blood pressure | No | Yes |
| *Millettia ferruginea* (Hochst.) Hochst. ex Baker | Gonorrhea | No | Yes |
| *Millettia ferruginea* (Hochst.) Hochst. ex Baker | Typhoid | No | Yes |
| *Momordica boivinii* Baill. | Amoeba | No | Yes |
| *Momordica boivinii* Baill. | Jaundice | No | Yes |
| *Momordica boivinii* Baill. | Lung infection | No | Yes |
| *Momordica boivinii* Baill. | Spiritual | No | Yes |
| *Momordica boivinii* Baill. | Stomachache | No | Yes |
| *Momordica boivinii* Baill. | Toothache | No | Yes |
| *Nicotiana tabacum* L. | Depression | No | Yes |
| *Nicotiana tabacum* L. | Wound | No | Yes |
| *Nigella sativa* L. | Common cold | No | Yes |
| *Nigella sativa* L. | Febrile illness | No | Yes |
| *Nigella sativa* L. | Skin infection | No | Yes |
| *Ocimum jamesii* Sebald | Febrile illness | No | Yes |
| *Ocimum lamiifolium* Hochst. ex Benth. | Headache | No | Yes |
| *Ocimum lamiifolium* Hochst. ex Benth. | Malaria | No | Yes |
| *Olea europaea subsp. cuspidata* (Wall. & G.Don) Cif. | Wound | No | Yes |
| *Olinia rochetiana* A.Juss. | Stomachache | No | Yes |
| *Persea americana* Mill. | Passive sexual interest | No | Yes |
| *Phytolacca dodecandra* L'Hér. | Amoeba | No | Yes |
| *Phytolacca dodecandra* L'Hér. | Gonorrhea | No | Yes |
| *Phytolacca dodecandra* L'Hér. | Swellings | No | Yes |
| *Pittosporum abyssinicum* Delile | Tuberculosis | No | Yes |
| *Coleus igniarius* Schweinf. | Amoeba | No | Yes |
| *Coleus igniarius* Schweinf. | Evil eye | No | Yes |
| *Coleus igniarius* Schweinf. | Febrile illness | No | Yes |
| *Coleus igniarius* Schweinf. | Skin infection | No | Yes |
| *Coleus igniarius* Schweinf. | Spiritual | No | Yes |
| *Coleus igniarius* Schweinf. | Wound | No | Yes |
| *Premna schimperi* Engl. | Febrile illness | No | Yes |
| *Premna schimperi* Engl. | Lung infection | No | Yes |
| *Psidium guajava* L. | Diabetes | No | Yes |
| *Psidium guajava* L. | Stomachache | No | Yes |
| *Rhamnus prinoides* L'Hér. | Gonorrhea | No | Yes |
| *Rhamnus prinoides* L'Hér. | Skin infection | No | Yes |
| *Rhamnus prinoides* L'Hér. | Stomachache | No | Yes |
| *Rhamnus prinoides* L'Hér. | Tonsillitis | No | Yes |
| *Searsia pyroides* (Burch.) Moffett | Glandular | No | Yes |
| *Searsia pyroides* (Burch.) Moffett | Lung infection | No | Yes |
| *Searsia pyroides* (Burch.) Moffett | Spiritual | No | Yes |
| *Ricinus communis* L. | Jaundice | No | Yes |
| *Ricinus communis* L. | Lung infection | No | Yes |
| *Rotheca myricoides* (Hochst.) Steane & Mabb. | Amoeba | No | Yes |
| *Rotheca myricoides* (Hochst.) Steane & Mabb. | Cancer | No | Yes |
| *Rotheca myricoides* (Hochst.) Steane & Mabb. | Diarrhea | No | Yes |
| *Rotheca myricoides* (Hochst.) Steane & Mabb. | Glandular | No | Yes |
| *Rotheca myricoides* (Hochst.) Steane & Mabb. | Jaundice | No | Yes |
| *Rotheca myricoides* (Hochst.) Steane & Mabb. | Lung infection | No | Yes |
| *Rotheca myricoides* (Hochst.) Steane & Mabb. | Skin infection | No | Yes |
| *Rumex abyssinicus* Jacq. | Amoeba | No | Yes |
| *Rumex abyssinicus* Jacq. | Gastric diseases | No | Yes |
| *Rumex abyssinicus* Jacq. | Skin infection | No | Yes |
| *Rumex nepalensis* Spreng. | Stomachache | No | Yes |
| *Ruta chalepensis* L. | Diarrhea | No | Yes |
| *Ruta chalepensis* L. | Goiter | No | Yes |
| *Ruta chalepensis* L. | Skin infection | No | Yes |
| *Ruta chalepensis* L. | Spiritual | No | Yes |
| *Ruta chalepensis* L. | Tuberculosis | No | Yes |
| *Searsia natalensis* (Bernh. ex Krauss) F.A.Barkley | Snake poison | No | Yes |
| *Solanum incanum* L. | Nasal bleeding | No | Yes |
| *Solanum incanum* L. | Snake poison | No | Yes |
| *Solanum incanum* L. | Spiritual | No | Yes |
| *Stephania abyssinica* (Quart.-Dill. & A.Rich.) Walp. | Glandular | No | Yes |
| *Stephania abyssinica* (Quart.-Dill. & A.Rich.) Walp. | Jaundice | No | Yes |
| *Syzygium guineense* (Willd.) DC. | Amoeba | No | Yes |
| *Syzygium guineense* (Willd.) DC. | Diarrhea | No | Yes |
| *Syzygium guineense* (Willd.) DC. | Muscle pain | No | Yes |
| *Syzygium guineense* (Willd.) DC. | Spiritual | No | Yes |
| *Taverniera abyssinica* A.Rich. | Febrile illness | No | Yes |
| *Taverniera abyssinica* A.Rich. | Fever | No | Yes |
| *Taverniera abyssinica* A.Rich. | Headache | No | Yes |
| *Thymus schimperi* Ronniger | Cholesterol | No | Yes |
| *Trigonella foenum-graecum* L. | Cholesterol | No | Yes |
| *Trigonella foenum-graecum* L. | Kidney infection | No | Yes |
| *Triticum turgidum subsp. dicoccum* (Schrank ex Schübl.) Thell. | Wound | No | Yes |
| *Urtica dioica* L. | Amoeba | No | Yes |
| *Urtica dioica* L. | Gonorrhea | No | Yes |
| *Urtica dioica* L. | Spiritual | No | Yes |
| *Urtica simensis* Hochst. ex A.Rich. | Fire accident | No | Yes |
| *Urtica simensis* Hochst. ex A.Rich. | Spiritual | No | Yes |
| *Gymnanthemum amygdalinum* (Delile) Sch.Bip. | Head skin infection | No | Yes |
| *Gymnanthemum auriculiferum* (Hiern) Isawumi | Spiritual | No | Yes |
| *Withania somnifera* (L.) Dunal | Asthma | No | Yes |
| *Withania somnifera* (L.) Dunal | Cough | No | Yes |
| *Zehneria scabra* (L.f.) Sond. | Cancer | No | Yes |
| *Zingiber officinale* Roscoe | Asthma | No | Yes |
| *Zingiber officinale* Roscoe | Blood pressure | No | Yes |
| *Zingiber officinale* Roscoe | Cough | No | Yes |
| *Zingiber officinale* Roscoe | Fever | No | Yes |
| *Zingiber officinale* Roscoe | Goiter | No | Yes |
| *Zingiber officinale* Roscoe | Headache | No | Yes |
| *Zingiber officinale* Roscoe | Malaria | No | Yes |
| *Zingiber officinale* Roscoe | Passive sexual interest | No | Yes |
| *Zingiber officinale* Roscoe | Typhoid | No | Yes |
