# Supplementary material for: Ethnobotanical study on medicinal plant knowledge among three ethnic groups in peri-urban areas of south-central Ethiopia
Source: J Ethnobiol Ethnomed. 2023 Nov 23;19:55. doi: 10.1186/s13002-023-00629-w (PMC10668360; doi:10.1186/s13002-023-00629-w)
Supplement: Supplementary file 4 — Additional file 4. Rahman similarity index between Oromo and Sidama ethnic groups. [file 13002_2023_629_MOESM4_ESM.docx]

Supplementary Table 4: Rahman similarity index between Oromo and Sidama ethnic groups. ‘Yes’ indicates shared medicinal plants to treat the same ailments between the two ethnic groups. Whereas ‘Yes or No’ indicates a disparity between ethnic groups.

|  | | **Ethnic groups** | |
| --- | --- | --- | --- |
| **Species** | **Ailment** | **Oromo** | **Sidama** |
| *Achyranthes aspera* L. | Stomachache | Yes | Yes |
| *Acokanthera schimperi* (A.DC.) Benth. & Hook.f. ex Schweinf. | Spiritual | Yes | Yes |
| *Albizia gummifera* (J.F.Gmel.) C.A.Sm. | Dizziness | Yes | Yes |
| *Albizia gummifera* (J.F.Gmel.) C.A.Sm. | Stomachache | Yes | Yes |
| *Allium sativum* L. | Common cold | Yes | Yes |
| *Allium sativum* L. | Febrile illness | Yes | Yes |
| *Allium sativum* L. | Fever | Yes | Yes |
| *Allium sativum* L. | Tonsillitis | Yes | Yes |
| *Allium sativum* L. | Typhoid | Yes | Yes |
| *Aloe macrocarpa* Tod. | Malaria | Yes | Yes |
| *Aloe vera* (L.) Burm.f. | Stomachache | Yes | Yes |
| *Artemisia abyssinica* Sch.Bip. ex A.Rich. | Spiritual | Yes | Yes |
| *Calpurnia aurea* (Aiton) Benth. | Typhoid | Yes | Yes |
| *Carica papaya* L. | Intestinal worms | Yes | Yes |
| *Carica papaya* L. | Malaria | Yes | Yes |
| *Catha edulis* (Vahl) Forssk. ex Endl. | Gonorrhea | Yes | Yes |
| *Clutia abyssinica* Jaub. & Spach | Cancer | Yes | Yes |
| *Clutia abyssinica* Jaub. & Spach | Spiritual | Yes | Yes |
| *Clutia abyssinica* Jaub. & Spach | Swellings | Yes | Yes |
| *Coffea arabica* L. | Gastric diseases | Yes | Yes |
| *Coffea arabica* L. | Wound | Yes | Yes |
| *Croton macrostachyus* Hochst. ex Delile | Amoeba | Yes | Yes |
| *Croton macrostachyus* Hochst. ex Delile | Cancer | Yes | Yes |
| *Croton macrostachyus* Hochst. ex Delile | Diarrhea | Yes | Yes |
| *Croton macrostachyus* Hochst. ex Delile | Dizziness | Yes | Yes |
| *Croton macrostachyus* Hochst. ex Delile | Eye infection | Yes | Yes |
| *Croton macrostachyus* Hochst. ex Delile | Febrile illness | Yes | Yes |
| *Croton macrostachyus* Hochst. ex Delile | Giardia | Yes | Yes |
| *Croton macrostachyus* Hochst. ex Delile | Gonorrhea | Yes | Yes |
| *Croton macrostachyus* Hochst. ex Delile | Jaundice | Yes | Yes |
| *Croton macrostachyus* Hochst. ex Delile | Lung infection | Yes | Yes |
| *Croton macrostachyus* Hochst. ex Delile | Tetanus | Yes | Yes |
| *Croton macrostachyus* Hochst. ex Delile | Wound | Yes | Yes |
| *Datura stramonium* L. test | Skin infection | Yes | Yes |
| *Dodonaea viscosa subsp. angustifolia* (L.f.) J.G.West | Lung infection | Yes | Yes |
| *Ekebergia capensis* Sparrm. | Fever | Yes | Yes |
| *Ekebergia capensis* Sparrm. | Stomachache | Yes | Yes |
| *Eucalyptus globulus* Labill. | Asthma | Yes | Yes |
| *Eucalyptus globulus* Labill. | Bath of mother after giving a birth | Yes | Yes |
| *Eucalyptus globulus* Labill. | Common cold | Yes | Yes |
| *Eucalyptus globulus* Labill. | Headache | Yes | Yes |
| *Euclea divinorum* Hiern | Intestinal worms | Yes | Yes |
| *Lactuca inermis* Forssk. | Weight loss | Yes | Yes |
| *Lagenaria siceraria* (Molina) Standl. | Lung infection | Yes | Yes |
| *Melia azedarach* L. | Blood pressure | Yes | Yes |
| *Melia azedarach* L. | Depression | Yes | Yes |
| *Melia azedarach* L. | Diarrhea | Yes | Yes |
| *Melia azedarach* L. | Stomachache | Yes | Yes |
| *Mentha spicata* L. | Blood pressure | Yes | Yes |
| *Millettia ferruginea* (Hochst.) Hochst. ex Baker | Gonorrhea | Yes | Yes |
| *Moringa stenopetala* (Baker f.) Cufod. | Blood pressure | Yes | Yes |
| *Moringa stenopetala* (Baker f.) Cufod. | Glandular | Yes | Yes |
| *Nigella sativa* L. | Common cold | Yes | Yes |
| *Nigella sativa* L. | Febrile illness | Yes | Yes |
| *Ocimum jamesii* Sebald | Febrile illness | Yes | Yes |
| *Ocimum lamiifolium* Hochst. ex Benth. | Febrile illness | Yes | Yes |
| *Olea europaea subsp. cuspidata* (Wall. & G.Don) Cif. | Breast cancer | Yes | Yes |
| *Olea europaea subsp. cuspidata* (Wall. & G.Don) Cif. | Wound | Yes | Yes |
| *Olinia rochetiana* A.Juss. | Glandular | Yes | Yes |
| *Olinia rochetiana* A.Juss. | Stomachache | Yes | Yes |
| *Phytolacca dodecandra* L'Hér. | Abortion | Yes | Yes |
| *Phytolacca dodecandra* L'Hér. | Intestinal worms | Yes | Yes |
| *Phytolacca dodecandra* L'Hér. | Stomachache | Yes | Yes |
| *Psidium guajava* L. | Blood pressure | Yes | Yes |
| *Rhamnus prinoides* L'Hér. | Tonsillitis | Yes | Yes |
| *Searsia glutinosa* (Hochst. ex A.Rich.) Moffett | Glandular | Yes | Yes |
| *Ricinus communis* L. | Lung infection | Yes | Yes |
| *Ricinus communis* L. | Swellings | Yes | Yes |
| *Ruta chalepensis* L. | Febrile illness | Yes | Yes |
| *Ruta chalepensis* L. | Gonorrhea | Yes | Yes |
| *Ruta chalepensis* L. | Tuberculosis | Yes | Yes |
| *Ruta chalepensis* L. | Typhoid | Yes | Yes |
| *Solanum incanum* L. | Nasal bleeding | Yes | Yes |
| *Solanum incanum* L. | Snake poison | Yes | Yes |
| *Stephania abyssinica* (Quart.-Dill. & A.Rich.) Walp. | Jaundice | Yes | Yes |
| *Taverniera abyssinica* A.Rich. | Fever | Yes | Yes |
| *Gymnanthemum amygdalinum* (Delile) Sch.Bip. | Amoeba | Yes | Yes |
| *Gymnanthemum amygdalinum* (Delile) Sch.Bip. | Diarrhea | Yes | Yes |
| *Gymnanthemum amygdalinum* (Delile) Sch.Bip. | Head skin infection | Yes | Yes |
| *Gymnanthemum amygdalinum* (Delile) Sch.Bip. | Malaria | Yes | Yes |
| *Gymnanthemum amygdalinum* (Delile) Sch.Bip. | Skin infection | Yes | Yes |
| *Gymnanthemum amygdalinum* (Delile) Sch.Bip. | Stomachache | Yes | Yes |
| *Withania somnifera* (L.) Dunal | Asthma | Yes | Yes |
| *Withania somnifera* (L.) Dunal | Cough | Yes | Yes |
| *Withania somnifera* (L.) Dunal | Spiritual | Yes | Yes |
| *Zehneria scabra* (L.f.) Sond. | Cancer | Yes | Yes |
| *Zingiber officinale* Roscoe | Blood pressure | Yes | Yes |
| *Zingiber officinale* Roscoe | Common cold | Yes | Yes |
| *Zingiber officinale* Roscoe | Fever | Yes | Yes |
| *Zingiber officinale* Roscoe | Passive sexual interest | Yes | Yes |
| *Zingiber officinale* Roscoe | Tonsillitis | Yes | Yes |
| *Zingiber officinale* Roscoe | Typhoid | Yes | Yes |
| *Zingiber officinale* Roscoe | Wound | Yes | Yes |
| *Vachellia oerfota* (Forssk.) Kyal. & Boatwr. | General health | Yes | No |
| *Vachellia oerfota* (Forssk.) Kyal. & Boatwr. | Spiritual | Yes | No |
| *Vachellia seyal* (Delile) P.J.H.Hurter | Intestinal worms | Yes | No |
| *Vachellia tortilis (*Forssk.) Galasso & Banfi | Malaria | Yes | No |
| *Vachellia tortilis (*Forssk.) Galasso & Banfi | Spiritual | Yes | No |
| *Achyranthes aspera* L. | Diarrhea | Yes | No |
| *Achyranthes aspera* L. | Respiratory organ infection | Yes | No |
| *Achyranthes aspera* L. | Skin infection | Yes | No |
| *Achyranthes aspera* L. | Spiritual | Yes | No |
| *Acokanthera schimperi* (A.DC.) Benth. & Hook.f. ex Schweinf. | Skin infection | Yes | No |
| *Acokanthera schimperi* (A.DC.) Benth. & Hook.f. ex Schweinf. | Wound | Yes | No |
| *Albizia gummifera* (J.F.Gmel.) C.A.Sm. | Epilepsy | Yes | No |
| *Albizia gummifera* (J.F.Gmel.) C.A.Sm. | Febrile illness | Yes | No |
| *Albizia gummifera* (J.F.Gmel.) C.A.Sm. | Fire accident | Yes | No |
| *Albizia gummifera* (J.F.Gmel.) C.A.Sm. | Malaria | Yes | No |
| *Albizia gummifera* (J.F.Gmel.) C.A.Sm. | Skin infection | Yes | No |
| *Allium sativum* L. | Blood pressure | Yes | No |
| *Aloe macrocarpa* Tod. | Breast cancer | Yes | No |
| *Aloe macrocarpa* Tod. | Cancer | Yes | No |
| *Aloe macrocarpa* Tod. | Diarrhea | Yes | No |
| *Aloe macrocarpa* Tod. | Fever | Yes | No |
| *Aloe macrocarpa* Tod. | Gonorrhea | Yes | No |
| *Aloe macrocarpa* Tod. | Intestinal worms | Yes | No |
| *Aloe macrocarpa* Tod. | Jaundice | Yes | No |
| *Aloe macrocarpa* Tod. | Lung infection | Yes | No |
| *Aloe macrocarpa* Tod. | Pain relief | Yes | No |
| *Aloe macrocarpa* Tod. | Stomachache | Yes | No |
| *Aloe macrocarpa* Tod. | Typhoid | Yes | No |
| *Aloe macrocarpa* Tod. | Urinary organ infection | Yes | No |
| *Aloe macrocarpa* Tod. | Wound | Yes | No |
| *Aloe pirottae* A.Berger | Gastric diseases | Yes | No |
| *Aloe pirottae* A.Berger | Jaundice | Yes | No |
| *Aloe pirottae* A.Berger | Kidney infection | Yes | No |
| *Aloe pirottae* A.Berger | Menstruation cycle disorder | Yes | No |
| *Aloe pirottae* A.Berger | Passive sexual interest | Yes | No |
| *Aloe pirottae* A.Berger | Vaginal infection | Yes | No |
| *Argemone mexicana* L. | Blood pressure | Yes | No |
| *Argemone mexicana* L. | Cancer | Yes | No |
| *Argemone mexicana* L. | Jaundice | Yes | No |
| *Argemone mexicana* L. | Wound | Yes | No |
| *Artemisia abyssinica* Sch.Bip. ex A.Rich. | Blood pressure | Yes | No |
| *Artemisia abyssinica* Sch.Bip. ex A.Rich. | Malaria | Yes | No |
| *Artemisia abyssinica* Sch.Bip. ex A.Rich. | Nasal bleeding | Yes | No |
| *Asparagus africanus* Lam*.* | Breast cancer | Yes | No |
| *Asparagus africanus* Lam*.* | Ear infection | Yes | No |
| *Asparagus africanus* Lam*.* | Jaundice | Yes | No |
| *Asparagus africanus* Lam*.* | Rabies | Yes | No |
| *Asparagus africanus* Lam*.* | Skin infection | Yes | No |
| *Balanites aegyptiaca* (L.) Delile | Headache | Yes | No |
| *Balanites aegyptiaca* (L.) Delile | Mental case | Yes | No |
| *Bersama abyssinica* Fresen. | Cancer | Yes | No |
| *Bersama abyssinica* Fresen. | Intestinal worms | Yes | No |
| *Beta vulgaris* L. | Anemia | Yes | No |
| *Beta vulgaris* L. | Wound | Yes | No |
| *Brassica carinata* A.Braun | Constipation | Yes | No |
| *Brassica carinata* A.Braun | Fever | Yes | No |
| *Brassica carinata* A.Braun | Skin infection | Yes | No |
| *Brassica carinata* A.Braun | Toothache | Yes | No |
| *Brucea antidysenterica* J.F.Mill. | Diarrhea | Yes | No |
| *Calendula officinalis* L. | Amoeba | Yes | No |
| *Calpurnia aurea* (Aiton) Benth. | Amoeba | Yes | No |
| *Calpurnia aurea* (Aiton) Benth. | Circumcision wound | Yes | No |
| *Calpurnia aurea* (Aiton) Benth. | Fever | Yes | No |
| *Calpurnia aurea* (Aiton) Benth. | Headache | Yes | No |
| *Calpurnia aurea* (Aiton) Benth. | Skin infection | Yes | No |
| *Calpurnia aurea* (Aiton) Benth. | Stomachache | Yes | No |
| *Calpurnia aurea* (Aiton) Benth. | Toothache | Yes | No |
| *Calpurnia aurea* (Aiton) Benth. | Wound | Yes | No |
| *Capsella bursa-pastoris* Medik. | Asthma | Yes | No |
| *Capsella bursa-pastoris* Medik. | Cough | Yes | No |
| *Capsella bursa-pastoris* Medik. | Lung infection | Yes | No |
| *Carica papaya* L. | Bath of mother after giving a birth | Yes | No |
| *Carica papaya* L. | Blood pressure | Yes | No |
| *Carica papaya* L. | Cancer | Yes | No |
| *Carica papaya* L. | Typhoid | Yes | No |
| *Carissa spinarum* L. | Febrile illness | Yes | No |
| *Carissa spinarum* L. | Headache | Yes | No |
| *Carissa spinarum* L. | Spiritual | Yes | No |
| *Casimiroa edulis* La Llave | Gastric diseases | Yes | No |
| *Casuarina equisetifolia* L. | Rabies | Yes | No |
| *Catha edulis* (Vahl) Forssk. ex Endl. | Diarrhea | Yes | No |
| *Catha edulis* (Vahl) Forssk. ex Endl. | Skin infection | Yes | No |
| *Citrus × aurantiifolia* (Christm.) Swingle | Blood pressure | Yes | No |
| *Citrus × aurantiifolia* (Christm.) Swingle | Giardia | Yes | No |
| *Citrus limon* (L.) Osbeck | Amoeba | Yes | No |
| *Citrus limon* (L.) Osbeck | Blood pressure | Yes | No |
| *Citrus limon* (L.) Osbeck | Common cold | Yes | No |
| *Citrus limon* (L.) Osbeck | Fever | Yes | No |
| *Citrus limon* (L.) Osbeck | Stomachache | Yes | No |
| *Clematis* hirsuta Perr. & Guill. | Cancer | Yes | No |
| *Clematis* hirsuta Perr. & Guill. | Wound | Yes | No |
| *Rotheca myricoides* (Hochst.) Steane & Mabb. | Rabies | Yes | No |
| *Rotheca myricoides* (Hochst.) Steane & Mabb. | Spiritual | Yes | No |
| *Rotheca myricoides* (Hochst.) Steane & Mabb. | Stomachache | Yes | No |
| *Clutia abyssinica* Jaub. & Spach | Bone cancer | Yes | No |
| *Clutia abyssinica* Jaub. & Spach | Snake poison | Yes | No |
| *Clutia abyssinica* Jaub. & Spach | Wound | Yes | No |
| *Coffea arabica* L. | Cancer | Yes | No |
| *Coffea arabica* L. | Kidney infection | Yes | No |
| *Coffea arabica* L. | Sneezing | Yes | No |
| *Commelina benghalensis* L. | Swellings | Yes | No |
| *Cordia africana* Lam. | Blood pressure | Yes | No |
| *Cordia africana* Lam. | Diarrhea | Yes | No |
| *Cordia africana* Lam. | Passive sexual interest | Yes | No |
| *Cordia africana* Lam. | Spiritual | Yes | No |
| *Croton macrostachyus* Hochst. ex Delile | Abortion | Yes | No |
| *Croton macrostachyus* Hochst. ex Delile | Asthma | Yes | No |
| *Croton macrostachyus* Hochst. ex Delile | Breast cancer | Yes | No |
| *Croton macrostachyus* Hochst. ex Delile | Circumcision wound | Yes | No |
| *Croton macrostachyus* Hochst. ex Delile | Intestinal worms | Yes | No |
| *Croton macrostachyus* Hochst. ex Delile | Malaria | Yes | No |
| *Croton macrostachyus* Hochst. ex Delile | Placental delay during birth | Yes | No |
| *Croton macrostachyus* Hochst. ex Delile | Skin infection | Yes | No |
| *Croton macrostachyus* Hochst. ex Delile | Stomachache | Yes | No |
| *Croton macrostachyus* Hochst. ex Delile | Typhoid | Yes | No |
| *Cyathula polycephala* Baker | Febrile illness | Yes | No |
| *Datura stramonium* L. test | Rabies | Yes | No |
| *Datura stramonium* L. test | Toothache | Yes | No |
| *Daucus carota* L. | Jaundice | Yes | No |
| *Daucus carota* L. | Passive sexual interest | Yes | No |
| *Delonix elata* (L.) Gamble | Stomachache | Yes | No |
| *Dodonaea viscosa subsp. angustifolia* (L.f.) J.G.West | Bone injury | Yes | No |
| *Dodonaea viscosa subsp. angustifolia* (L.f.) J.G.West | Circumcision wound | Yes | No |
| *Dodonaea viscosa subsp. angustifolia* (L.f.) J.G.West | Gastric diseases | Yes | No |
| *Dodonaea viscosa subsp. angustifolia* (L.f.) J.G.West | Headache | Yes | No |
| *Dodonaea viscosa subsp. angustifolia* (L.f.) J.G.West | Stomachache | Yes | No |
| *Ehretia cymosa* Thonn. | Stomachache | Yes | No |
| *Ekebergia capensis* Sparrm. | Bone cancer | Yes | No |
| *Ekebergia capensis* Sparrm. | Cancer | Yes | No |
| *Ekebergia capensis* Sparrm. | Glandular | Yes | No |
| *Ekebergia capensis* Sparrm. | Placental delay during birth | Yes | No |
| *Ekebergia capensis* Sparrm. | Skin infection | Yes | No |
| *Ekebergia capensis* Sparrm. | Spiritual | Yes | No |
| *Ekebergia capensis* Sparrm. | Swellings | Yes | No |
| *Ekebergia capensis* Sparrm. | Wound | Yes | No |
| *Eleusine coracana* (L.) Gaertn. | Bone injury | Yes | No |
| *Eleusine coracana* (L.) Gaertn. | Wound | Yes | No |
| *Eragrostis tef* (Zuccagni) Trotter | Bone injury | Yes | No |
| *Eragrostis tef* (Zuccagni) Trotter | Wound | Yes | No |
| *Erica arborea* L. | Malaria | Yes | No |
| *Erica arborea* L. | Spiritual | Yes | No |
| *Erica arborea* L. | Wound | Yes | No |
| *Erythrina abyssinica* Lam. | Diarrhea | Yes | No |
| *Erythrina abyssinica* Lam. | Goiter | Yes | No |
| *Erythrina abyssinica* Lam. | Intestinal worms | Yes | No |
| *Erythrina abyssinica* Lam. | Lung infection | Yes | No |
| *Erythrina abyssinica* Lam. | Rabies | Yes | No |
| *Erythrina abyssinica* Lam. | Spiritual | Yes | No |
| *Corymbia citriodora* (Hook.) K.D.Hill & L.A.S.Johnson | Gonorrhea | Yes | No |
| *Eucalyptus globulus* Labill. | Nasal bleeding | Yes | No |
| *Euclea divinorum* Hiern | Circumcision wound | Yes | No |
| *Euclea divinorum* Hiern | Skin infection | Yes | No |
| *Euclea divinorum* Hiern | Weight loss | Yes | No |
| *Euphorbia ampliphylla* Pax | Cancer | Yes | No |
| *Euphorbia ampliphylla* Pax | Epilepsy | Yes | No |
| *Ficus sycomorus* L. | Glandular | Yes | No |
| *Ficus sycomorus* L. | Tonsillitis | Yes | No |
| *Grewia ferruginea* Hochst. ex A.Rich. | Respiratory organ infection | Yes | No |
| *Hagenia abyssinica* (Bruce) J.F.Gmel. | Amoeba | Yes | No |
| *Hagenia abyssinica* (Bruce) J.F.Gmel. | Diarrhea | Yes | No |
| *Hagenia abyssinica* (Bruce) J.F.Gmel. | Febrile illness | Yes | No |
| *Hagenia abyssinica* (Bruce) J.F.Gmel. | Gonorrhea | Yes | No |
| *Hagenia abyssinica* (Bruce) J.F.Gmel. | Tapeworm | Yes | No |
| *Helianthus annuus* L*.* | Febrile illness | Yes | No |
| *Helianthus annuus* L*.* | Tung infection | Yes | No |
| *Hordeum vulgare* L. | Bone injury | Yes | No |
| *Hordeum vulgare* L. | Wound | Yes | No |
| *Indigofera arrecta* Hochst. ex A.Rich. | General health | Yes | No |
| *Justicia schimperiana* (Hochst. ex Nees) T.Anderson | Ear infection | Yes | No |
| *Justicia schimperiana* (Hochst. ex Nees) T.Anderson | Glandular | Yes | No |
| *Justicia schimperiana* (Hochst. ex Nees) T.Anderson | Goiter | Yes | No |
| *Justicia schimperiana* (Hochst. ex Nees) T.Anderson | Jaundice | Yes | No |
| *Kalanchoe densiflora* Rolfe | Muscular/joint pain | Yes | No |
| *Kalanchoe petitiana* A.Rich. | Bone injury | Yes | No |
| *Kniphofia foliosa* Hochst. | Stomachache | Yes | No |
| *Lactuca inermis* Forssk. | Anemia | Yes | No |
| *Lactuca inermis* Forssk. | Febrile illness | Yes | No |
| *Lactuca inermis* Forssk. | Stomachache | Yes | No |
| *Lagenaria siceraria* (Molina) Standl. | Fever | Yes | No |
| *Lagenaria siceraria* (Molina) Standl. | Joint pain | Yes | No |
| *Lantana camara* L. | Sneezing | Yes | No |
| *Lepidium sativum* L. | Common cold | Yes | No |
| *Lepidium sativum* L. | Dry skin treatment | Yes | No |
| *Lepidium sativum* L. | Febrile illness | Yes | No |
| *Lepidium sativum* L. | Malaria | Yes | No |
| *Lepidium sativum* L. | Vaginal infection | Yes | No |
| *Lippia javanica* (Burm.f.) Spreng. | Blood pressure | Yes | No |
| *Lippia javanica* (Burm.f.) Spreng. | Diarrhea | Yes | No |
| *Lippia javanica* (Burm.f.) Spreng. | Stomachache | Yes | No |
| *Maesa lanceolata* Forssk. | Jaundice | Yes | No |
| *Maesa lanceolata* Forssk. | Muscular/joint pain | Yes | No |
| *Maesa lanceolata* Forssk. | Nerve case | Yes | No |
| *Maesa lanceolata* Forssk. | Skin infection | Yes | No |
| *Gymnosporia senegalensis* (Lam.) Loes. | Jaundice | Yes | No |
| *Gymnosporia senegalensis* (Lam.) Loes. | Malaria | Yes | No |
| *Gymnosporia senegalensis* (Lam.) Loes. | Skin infection | Yes | No |
| *Melia azedarach* L. | Cancer | Yes | No |
| *Melia azedarach* L. | Fever | Yes | No |
| *Melia azedarach* L. | Glandular | Yes | No |
| *Melia azedarach* L. | Intestinal worms | Yes | No |
| *Melia azedarach* L. | Jaundice | Yes | No |
| *Melia azedarach* L. | Toothache | Yes | No |
| *Melia azedarach* L. | Typhoid | Yes | No |
| *Millettia ferruginea* (Hochst.) Hochst. ex Baker | Blood pressure | Yes | No |
| *Millettia ferruginea* (Hochst.) Hochst. ex Baker | Stomachache | Yes | No |
| *Mimusops kummel* Bruce ex A.DC. | Diarrhea | Yes | No |
| *Mimusops kummel* Bruce ex A.DC. | Lung infection | Yes | No |
| *Moringa stenopetala* (Baker f.) Cufod. | Cancer | Yes | No |
| *Moringa stenopetala* (Baker f.) Cufod. | Cholesterol | Yes | No |
| *Moringa stenopetala* (Baker f.) Cufod. | Diarrhea | Yes | No |
| *Moringa stenopetala* (Baker f.) Cufod. | Gastric diseases | Yes | No |
| *Moringa stenopetala* (Baker f.) Cufod. | Intestinal worms | Yes | No |
| *Moringa stenopetala* (Baker f.) Cufod. | Jaundice | Yes | No |
| *Moringa stenopetala* (Baker f.) Cufod. | Kidney infection | Yes | No |
| *Moringa stenopetala* (Baker f.) Cufod. | Lung infection | Yes | No |
| *Moringa stenopetala* (Baker f.) Cufod. | Malaria | Yes | No |
| *Moringa stenopetala* (Baker f.) Cufod. | Nerve case | Yes | No |
| *Moringa stenopetala* (Baker f.) Cufod. | Pain relief | Yes | No |
| *Moringa stenopetala* (Baker f.) Cufod. | Typhoid | Yes | No |
| *Myrica salicifolia* Hochst. ex A.Rich. | Spiritual | Yes | No |
| *Nicotiana tabacum* L. | Common cold | Yes | No |
| *Nigella sativa* L. | Amoeba | Yes | No |
| *Nigella sativa* L. | Asthma | Yes | No |
| *Nigella sativa* L. | Fever | Yes | No |
| *Nigella sativa* L. | Malaria | Yes | No |
| *Nigella sativa* L. | Nasal bleeding | Yes | No |
| *Nigella sativa* L. | Nerve case | Yes | No |
| *Nigella sativa* L. | Pain relief | Yes | No |
| *Nigella sativa* L. | Stomachache | Yes | No |
| *Nuxia congesta* R.Br. ex Fresen. | Cancer | Yes | No |
| *Ocimum jamesii* Sebald | Worms | Yes | No |
| *Ocimum lamiifolium* Hochst. ex Benth. | Fever | Yes | No |
| *Ocimum lamiifolium* Hochst. ex Benth. | Stomachache | Yes | No |
| *Ocimum gratissimum* L. | Eye infection | Yes | No |
| *Ocimum gratissimum* L. | Febrile illness | Yes | No |
| *Ocimum gratissimum* L. | Fever | Yes | No |
| *Ocimum gratissimum* L. | Malaria | Yes | No |
| *Ocimum gratissimum* L. | Stomachache | Yes | No |
| *Ocimum gratissimum* L. | Vomiting | Yes | No |
| *Olea europaea subsp. cuspidata* (Wall. & G.Don) Cif. | Anemia | Yes | No |
| *Olea europaea subsp. cuspidata* (Wall. & G.Don) Cif. | Asthma | Yes | No |
| *Olea europaea subsp. cuspidata* (Wall. & G.Don) Cif. | Blood pressure | Yes | No |
| *Olea europaea subsp. cuspidata* (Wall. & G.Don) Cif. | Cancer | Yes | No |
| *Olea europaea subsp. cuspidata* (Wall. & G.Don) Cif. | Cough | Yes | No |
| *Olea europaea subsp. cuspidata* (Wall. & G.Don) Cif. | Intestinal worms | Yes | No |
| *Olea europaea subsp. cuspidata* (Wall. & G.Don) Cif. | Jaundice | Yes | No |
| *Olea europaea subsp. cuspidata* (Wall. & G.Don) Cif. | Kidney infection | Yes | No |
| *Olea europaea subsp. cuspidata* (Wall. & G.Don) Cif. | Malaria | Yes | No |
| *Olea europaea subsp. cuspidata* (Wall. & G.Don) Cif. | Pain relief | Yes | No |
| *Olea europaea subsp. cuspidata* (Wall. & G.Don) Cif. | Respiratory organ infection | Yes | No |
| *Olea europaea subsp. cuspidata* (Wall. & G.Don) Cif. | Spiritual | Yes | No |
| *Olea europaea subsp. cuspidata* (Wall. & G.Don) Cif. | Swellings | Yes | No |
| *Olea europaea subsp. cuspidata* (Wall. & G.Don) Cif. | Toothache | Yes | No |
| *Olea europaea subsp. cuspidata* (Wall. & G.Don) Cif. | Tuberculosis | Yes | No |
| *Olea europaea subsp. cuspidata* (Wall. & G.Don) Cif. | Vaginal infection | Yes | No |
| *Olinia rochetiana* A.Juss. | Cancer | Yes | No |
| *Olinia rochetiana* A.Juss. | Circumcision wound | Yes | No |
| *Olinia rochetiana* A.Juss. | Skin infection | Yes | No |
| *Olinia rochetiana* A.Juss. | Toothache | Yes | No |
| *Olinia rochetiana* A.Juss. | Tuberculosis | Yes | No |
| *Olinia rochetiana* A.Juss. | Wound | Yes | No |
| *Pavonia urens* Cav. | Spiritual | Yes | No |
| *Persea americana* Mill. | Amoeba | Yes | No |
| *Persea americana* Mill. | Blood pressure | Yes | No |
| *Persicaria senegalensis* (Meisn.) Soják | Jaundice | Yes | No |
| *Persicaria senegalensis* (Meisn.) Soják | Malaria | Yes | No |
| *Pittosporum abyssinicum* Delile | Intestinal worms | Yes | No |
| *Pittosporum abyssinicum* Delile | Fever | Yes | No |
| *Pittosporum abyssinicum* Delile | Rabies | Yes | No |
| *Pittosporum abyssinicum* Delile | Spiritual | Yes | No |
| *Plantago lanceolata* L. | Epilepsy | Yes | No |
| *Afrocarpus falcatus* (Thunb.) C.N.Page | Typhoid | Yes | No |
| *Aningeria altissima* (A.Chev.) Aubrév. & Pellegr. | Swellings | Yes | No |
| *Prunus africana* (Hook.f.) Kalkman | Skin infection | Yes | No |
| *Psidium guajava* L. | Cancer | Yes | No |
| *Psidium guajava* L. | Intestinal worms | Yes | No |
| *Psidium guajava* L. | Typhoid | Yes | No |
| *Psydrax schimperianus* (A.Rich.) Bridson | Blood pressure | Yes | No |
| *Psydrax schimperianus* (A.Rich.) Bridson | Cancer | Yes | No |
| *Psydrax schimperianus* (A.Rich.) Bridson | Febrile illness | Yes | No |
| *Psydrax schimperianus* (A.Rich.) Bridson | Skin infection | Yes | No |
| *Psydrax schimperianus* (A.Rich.) Bridson | Wound | Yes | No |
| *Searsia pyroides* (Burch.) Moffett | Autism | Yes | No |
| *Searsia pyroides* (Burch.) Moffett | Breast cancer | Yes | No |
| *Searsia pyroides* (Burch.) Moffett | Passive sexual interest | Yes | No |
| *Rubus apetalus* Poir. | Toothache | Yes | No |
| *Rubus steudneri* Schweinf. | Amoeba | Yes | No |
| *Rubus steudneri* Schweinf. | Diarrhea | Yes | No |
| *Rubus steudneri* Schweinf. | Febrile illness | Yes | No |
| *Rubus steudneri* Schweinf. | Headache | Yes | No |
| *Rubus steudneri* Schweinf. | Nasal bleeding | Yes | No |
| *Rubus steudneri* Schweinf. | Skin infection | Yes | No |
| *Rubus steudneri* Schweinf. | Stomachache | Yes | No |
| *Rubus steudneri* Schweinf. | Urinary organ infection | Yes | No |
| *Rumex abyssinicus* Jacq. | Cancer | Yes | No |
| *Ruta chalepensis* L. | Dry skin treatment | Yes | No |
| *Ruta chalepensis* L. | Giardia | Yes | No |
| *Ruta chalepensis* L. | Jaundice | Yes | No |
| *Ruta chalepensis* L. | Malaria | Yes | No |
| *Ruta chalepensis* L. | Nasal bleeding | Yes | No |
| *Ruta chalepensis* L. | Nerve case | Yes | No |
| *Ruta chalepensis* L. | Placental delay during birth | Yes | No |
| *Ruta chalepensis* L. | Stomachache | Yes | No |
| *Ruta chalepensis* L. | Vomiting | Yes | No |
| *Salvia nilotica* Juss. ex Jacq. | Heart case | Yes | No |
| *Schinus molle* L. | Jaundice | Yes | No |
| *Schinus molle* L. | Nasal bleeding | Yes | No |
| *Schinus molle* L. | Tonsillitis | Yes | No |
| *Schrebera alata* (Hochst.) Welw. | Cancer | Yes | No |
| *Schrebera alata* (Hochst.) Welw. | Swellings | Yes | No |
| *Senna auriculata* (L.) Roxb. | Constipation | Yes | No |
| *Senna auriculata* (L.) Roxb. | Skin infection | Yes | No |
| *Sida schimperiana* Hochst. ex A.Rich. | Gonorrhea | Yes | No |
| *Sida schimperiana* Hochst. ex A.Rich. | Headache | Yes | No |
| *Sida schimperiana* Hochst. ex A.Rich. | Lung infection | Yes | No |
| *Sida schimperiana* Hochst. ex A.Rich. | Swellings | Yes | No |
| *Solanecio gigas* (Vatke) C.Jeffrey | Lung infection | Yes | No |
| *Solanum incanum* L. | Diarrhea | Yes | No |
| *Solanum marginatum* L.f. | Acid injury | Yes | No |
| *Solanum marginatum* L.f. | Autism | Yes | No |
| *Solanum marginatum* L.f. | Febrile illness | Yes | No |
| *Solanum marginatum* L.f. | Nasal bleeding | Yes | No |
| *Solanum marginatum* L.f. | Snake poison | Yes | No |
| *Solanum marginatum* L.f. | Spiritual | Yes | No |
| *Stephania abyssinica* (Quart.-Dill. & A.Rich.) Walp. | Gonorrhea | Yes | No |
| *Stephania abyssinica* (Quart.-Dill. & A.Rich.) Walp. | Lung infection | Yes | No |
| *Syzygium guineense* (Willd.) DC. | Circumcision wound | Yes | No |
| *Syzygium guineense* (Willd.) DC. | Glandular | Yes | No |
| *Syzygium guineense* (Willd.) DC. | Lung infection | Yes | No |
| *Syzygium guineense* (Willd.) DC. | Skin infection | Yes | No |
| *Syzygium guineense* (Willd.) DC. | Weight loss | Yes | No |
| *Vepris nobilis* (Delile) Mziray | Blood pressure | Yes | No |
| *Vepris nobilis* (Delile) Mziray | Dry skin treatment | Yes | No |
| *Vepris nobilis* (Delile) Mziray | Ear infection | Yes | No |
| *Vepris nobilis* (Delile) Mziray | Eye infection | Yes | No |
| *Vepris nobilis* (Delile) Mziray | Skin infection | Yes | No |
| *Terminalia brownii* Fresen. | Common cold | Yes | No |
| *Terminalia brownii* Fresen. | Headache | Yes | No |
| *Trichilia dregeana* Sond. | Jaundice | Yes | No |
| *Urtica dioica* L. | Febrile illness | Yes | No |
| *Urtica simensis* Hochst. ex A.Rich. | Febrile illness | Yes | No |
| *Urtica simensis* Hochst. ex A.Rich. | Gastric diseases | Yes | No |
| *Gymnanthemum amygdalinum* (Delile) Sch.Bip. | Blood pressure | Yes | No |
| *Gymnanthemum amygdalinum* (Delile) Sch.Bip. | Febrile illness | Yes | No |
| *Gymnanthemum amygdalinum* (Delile) Sch.Bip. | Gastric diseases | Yes | No |
| *Gymnanthemum amygdalinum* (Delile) Sch.Bip. | Gonorrhea | Yes | No |
| *Gymnanthemum amygdalinum* (Delile) Sch.Bip. | Intestinal worms | Yes | No |
| *Gymnanthemum amygdalinum* (Delile) Sch.Bip. | Jaundice | Yes | No |
| *Gymnanthemum amygdalinum* (Delile) Sch.Bip. | Lung infection | Yes | No |
| *Gymnanthemum amygdalinum* (Delile) Sch.Bip. | Rabies | Yes | No |
| *Gymnanthemum amygdalinum* (Delile) Sch.Bip. | Vomiting | Yes | No |
| *Gymnanthemum auriculiferum* (Hiern) Isawumi | Snake poison | Yes | No |
| *Withania somnifera* (L.) Dunal | Febrile illness | Yes | No |
| *Withania somnifera* (L.) Dunal | Skin infection | Yes | No |
| *Ximenia americana* L. | Intestinal worms | Yes | No |
| *Ximenia americana* L. | Stomachache | Yes | No |
| *Ximenia americana* L. | Swellings | Yes | No |
| *Ximenia americana* L. | Wound | Yes | No |
| *Zea mays* L. | Sneezing | Yes | No |
| *Zehneria scabra* (L.f.) Sond. | Gastric diseases | Yes | No |
| *Zehneria scabra* (L.f.) Sond. | Spiritual | Yes | No |
| *Zehneria scabra* (L.f.) Sond. | Swellings | Yes | No |
| *Zingiber officinale* Roscoe | Amoeba | Yes | No |
| *Zingiber officinale* Roscoe | Constipation | Yes | No |
| *Zingiber officinale* Roscoe | Febrile illness | Yes | No |
| *Zingiber officinale* Roscoe | Gastric diseases | Yes | No |
| *Ziziphus spina-christi* (L.) Willd. | Eye infection | Yes | No |
| *Ziziphus spina-christi* (L.) Willd. | Giardia | Yes | No |
| *Ziziphus spina-christi* (L.) Willd. | Gonorrhea | Yes | No |
| *Ziziphus spina-christi* (L.) Willd. | Intestinal worms | Yes | No |
| *Ziziphus spina-christi* (L.) Willd. | Rabies | Yes | No |
| *Ziziphus spina-christi* (L.) Willd. | Skin infection | Yes | No |
| *Ziziphus spina-christi* (L.) Willd. | Spiritual | Yes | No |
| *Ziziphus spina-christi* (L.) Willd. | Wound | Yes | No |
| *Achyranthes aspera* L. | Cancer | No | Yes |
| *Achyranthes aspera* L. | Gonorrhea | No | Yes |
| *Achyranthes aspera* L. | Headache | No | Yes |
| *Achyranthes aspera* L. | Joint pain | No | Yes |
| *Achyranthes aspera* L. | Muscle pain | No | Yes |
| *Aframomum corrorima* (A.Braun) P.C.M.Jansen | Skin infection | No | Yes |
| *Aframomum corrorima* (A.Braun) P.C.M.Jansen | Tonsillitis | No | Yes |
| *Ajuga integrifolia* Buch.-Ham. ex D.Don | Anemia | No | Yes |
| *Ajuga integrifolia* Buch.-Ham. ex D.Don | Malaria | No | Yes |
| *Ajuga integrifolia* Buch.-Ham. ex D.Don | Pain relief | No | Yes |
| *Ajuga integrifolia* Buch.-Ham. ex D.Don | Stomachache | No | Yes |
| *Ajuga integrifolia* Buch.-Ham. ex D.Don | Weight loss | No | Yes |
| *Albizia gummifera* (J.F.Gmel.) C.A.Sm. | Amoeba | No | Yes |
| *Albizia gummifera* (J.F.Gmel.) C.A.Sm. | Cancer | No | Yes |
| *Albizia gummifera* (J.F.Gmel.) C.A.Sm. | Goiter | No | Yes |
| *Albizia gummifera* (J.F.Gmel.) C.A.Sm. | Jaundice | No | Yes |
| *Albizia gummifera* (J.F.Gmel.) C.A.Sm. | Lung infection | No | Yes |
| *Albizia gummifera* (J.F.Gmel.) C.A.Sm. | Toothache | No | Yes |
| *Allium sativum* L. | Chicken pox | No | Yes |
| *Allium sativum* L. | Gonorrhea | No | Yes |
| *Allium sativum* L. | Headache | No | Yes |
| *Allium sativum* L. | Malaria | No | Yes |
| *Aloe vera* (L.) Burm.f. | Amoeba | No | Yes |
| *Aloe vera* (L.) Burm.f. | Blood pressure | No | Yes |
| *Aloe vera* (L.) Burm.f. | Malaria | No | Yes |
| *Antiaris toxicaria* (J.F.Gmel.) Lesch. | Rabies | No | Yes |
| *Artemisia absinthium* L. | Diabetes | No | Yes |
| *Artemisia absinthium* L. | Spiritual | No | Yes |
| *Balanites aegyptiaca* (L.) Delile | Amoeba | No | Yes |
| *Balanites aegyptiaca* (L.) Delile | Diarrhea | No | Yes |
| *Balanites aegyptiaca* (L.) Delile | Stomachache | No | Yes |
| *Bersama abyssinica* Fresen. | Amoeba | No | Yes |
| *Bersama abyssinica* Fresen. | Jaundice | No | Yes |
| *Bersama abyssinica* Fresen. | Spiritual | No | Yes |
| *Brucea antidysenterica* J.F.Mill. | Gonorrhea | No | Yes |
| *Calpurnia aurea* (Aiton) Benth. | Intestinal worms | No | Yes |
| *Calpurnia aurea* (Aiton) Benth. | Jaundice | No | Yes |
| *Calpurnia aurea* (Aiton) Benth. | Lung infection | No | Yes |
| *Calpurnia aurea* (Aiton) Benth. | Spiritual | No | Yes |
| *Capsicum annuum* L. | Intestinal worms | No | Yes |
| *Carica papaya* L. | Fever | No | Yes |
| *Carica papaya* L. | Gastric diseases | No | Yes |
| *Carissa spinarum* L. | Diarrhea | No | Yes |
| *Carissa spinarum* L. | Gonorrhea | No | Yes |
| *Catha edulis* (Vahl) Forssk. ex Endl. | Amoeba | No | Yes |
| *Catha edulis* (Vahl) Forssk. ex Endl. | Depression | No | Yes |
| *Catha edulis* (Vahl) Forssk. ex Endl. | Spiritual | No | Yes |
| *Cinnamomum verum* J.Presl | Asthma | No | Yes |
| *Cinnamomum verum* J.Presl | Common cold | No | Yes |
| *Cinnamomum verum* J.Presl | Fever | No | Yes |
| *Citrus × aurantiifolia* (Christm.) Swingle | Amoeba | No | Yes |
| *Citrus × aurantiifolia* (Christm.) Swingle | Anemia | No | Yes |
| *Clematis* hirsuta Perr. & Guill. | Jaundice | No | Yes |
| *Clutia abyssinica* Jaub. & Spach | Diarrhea | No | Yes |
| *Coffea arabica* L. | Malaria | No | Yes |
| *Commelina africana* L. | Skin infection | No | Yes |
| *Coriandrum sativum* L. | Overall health | No | Yes |
| *Cordia africana* Lam. | Nerve case | No | Yes |
| *Croton macrostachyus* Hochst. ex Delile | Glandular | No | Yes |
| *Croton macrostachyus* Hochst. ex Delile | Lightning | No | Yes |
| *Croton macrostachyus* Hochst. ex Delile | Spiritual | No | Yes |
| *Cucumis dipsaceus* Ehrenb. ex Spach | Jaundice | No | Yes |
| *Cucumis prophetarum* L. | Amoeba | No | Yes |
| *Cucumis prophetarum* L. | Balanced diet | No | Yes |
| *Cucumis prophetarum* L. | Cancer | No | Yes |
| *Cucumis prophetarum* L. | Diarrhea | No | Yes |
| *Cucumis prophetarum* L. | Glandular | No | Yes |
| *Cucumis prophetarum* L. | Jaundice | No | Yes |
| *Cucumis prophetarum* L. | Lung infection | No | Yes |
| *Cucumis prophetarum* L. | Respiratory organ infection | No | Yes |
| *Cucumis prophetarum* L. | Rheumatic | No | Yes |
| *Cucurbita pepo* L. | Intestinal worms | No | Yes |
| *Cucurbita pepo* L. | Tapeworm | No | Yes |
| *Cynodon dactylon* (L.) Pers. | Swellings | No | Yes |
| *Cynoglossum coeruleum* Hochst. ex A.DC. | Lung infection | No | Yes |
| *Cynoglossum coeruleum* Hochst. ex A.DC. | Skin infection | No | Yes |
| *Datura stramonium* L. test | Head skin infection | No | Yes |
| *Dovyalis caffra* (Hook.f. & Harv.) Warb. | Snake poison | No | Yes |
| *Echinops kebericho* Mesfin | Common cold | No | Yes |
| *Echinops kebericho* Mesfin | Febrile illness | No | Yes |
| *Echinops kebericho* Mesfin | Fever | No | Yes |
| *Echinops kebericho* Mesfin | Headache | No | Yes |
| *Ehretia cymosa* Thonn. | Cancer | No | Yes |
| *Ehretia cymosa* Thonn. | Lung infection | No | Yes |
| *Ehretia cymosa* Thonn. | Nasal bleeding | No | Yes |
| *Ehretia cymosa* Thonn. | Skin infection | No | Yes |
| *Ehretia cymosa* Thonn. | Wound | No | Yes |
| *Ekebergia capensis* Sparrm. | Amoeba | No | Yes |
| *Ekebergia capensis* Sparrm. | Goiter | No | Yes |
| *Ekebergia capensis* Sparrm. | Gonorrhea | No | Yes |
| *Ekebergia capensis* Sparrm. | Jaundice | No | Yes |
| *Ekebergia capensis* Sparrm. | Tuberculosis | No | Yes |
| *Ekebergia capensis* Sparrm. | Typhoid | No | Yes |
| *Ensete ventricosum* (Welw.) Cheesman | Lightning | No | Yes |
| *Erythrina abyssinica* Lam. | Toothache | No | Yes |
| *Eucalyptus globulus* Labill. | Dry skin treatment | No | Yes |
| *Eucalyptus globulus* Labill. | Fever | No | Yes |
| *Eucalyptus globulus* Labill. | Mental case | No | Yes |
| *Eucalyptus globulus* Labill. | Nerve case | No | Yes |
| *Eucalyptus globulus* Labill. | Pain relief | No | Yes |
| *Eucalyptus globulus* Labill. | Skin infection | No | Yes |
| *Eucalyptus globulus* Labill. | Spiritual | No | Yes |
| *Euclea racemosa subsp. schimperi* (A.DC.) F.White | Stomachache | No | Yes |
| *Galinsoga quadriradiata* Ruiz & Pav. | Goiter | No | Yes |
| *Galinsoga quadriradiata* Ruiz & Pav. | Tonsillitis | No | Yes |
| *Impatiens ethiopica* Grey-Wilson | Gonorrhea | No | Yes |
| *Justicia schimperiana* (Hochst. ex Nees) T.Anderson | Amoeba | No | Yes |
| *Justicia schimperiana* (Hochst. ex Nees) T.Anderson | Gonorrhea | No | Yes |
| *Justicia schimperiana* (Hochst. ex Nees) T.Anderson | Rabies | No | Yes |
| *Justicia schimperiana* (Hochst. ex Nees) T.Anderson | Sneezing | No | Yes |
| *Justicia schimperiana* (Hochst. ex Nees) T.Anderson | Stomachache | No | Yes |
| *Kalanchoe petitiana* A.Rich. | Broken bone | No | Yes |
| *Kalanchoe petitiana* A.Rich. | Diarrhea | No | Yes |
| *Kalanchoe petitiana* A.Rich. | Glandular | No | Yes |
| *Kalanchoe petitiana* A.Rich. | Muscular/joint pain | No | Yes |
| *Kalanchoe petitiana* A.Rich. | Pain relief | No | Yes |
| *Lactuca inermis* Forssk. | Balanced diet | No | Yes |
| *Lagenaria siceraria* (Molina) Standl. | Glandular | No | Yes |
| *Lagenaria siceraria* (Molina) Standl. | Jaundice | No | Yes |
| *Vicia lens* (L.) Coss. & Germ. | Chicken pox | No | Yes |
| *Vicia lens* (L.) Coss. & Germ. | Spider poison | No | Yes |
| *Vicia lens* (L.) Coss. & Germ. | Wound | No | Yes |
| *Linum usitatissimum* L. | Blood pressure | No | Yes |
| *Linum usitatissimum* L. | Diabetes | No | Yes |
| *Linum usitatissimum* L. | Gastric diseases | No | Yes |
| *Linum usitatissimum* L. | Kidney infection | No | Yes |
| *Linum usitatissimum* L. | Weight loss | No | Yes |
| *Lippia abyssinica* (Otto & A.Dietr.) Cufod. | Blood pressure | No | Yes |
| *Melia azedarach* L. | Diabetes | No | Yes |
| *Melia azedarach* L. | Gastric diseases | No | Yes |
| *Melia azedarach* L. | Malaria | No | Yes |
| *Melia azedarach* L. | Nasal bleeding | No | Yes |
| *Melia azedarach* L. | Pain relief | No | Yes |
| *Millettia ferruginea* (Hochst.) Hochst. ex Baker | Amoeba | No | Yes |
| *Millettia ferruginea* (Hochst.) Hochst. ex Baker | Skin infection | No | Yes |
| *Millettia ferruginea* (Hochst.) Hochst. ex Baker | Typhoid | No | Yes |
| *Momordica boivinii* Baill. | Amoeba | No | Yes |
| *Momordica boivinii* Baill. | Jaundice | No | Yes |
| *Momordica boivinii* Baill. | Lung infection | No | Yes |
| *Momordica boivinii* Baill. | Spiritual | No | Yes |
| *Momordica boivinii* Baill. | Stomachache | No | Yes |
| *Momordica boivinii* Baill. | Toothache | No | Yes |
| *Nicotiana tabacum* L. | Depression | No | Yes |
| *Nicotiana tabacum* L. | Headache | No | Yes |
| *Nicotiana tabacum* L. | Wound | No | Yes |
| *Nigella sativa* L. | Respiratory organ infection | No | Yes |
| *Nigella sativa* L. | Skin infection | No | Yes |
| *Ocimum lamiifolium* Hochst. ex Benth. | Headache | No | Yes |
| *Ocimum lamiifolium* Hochst. ex Benth. | Malaria | No | Yes |
| *Olea europaea subsp. cuspidata* (Wall. & G.Don) Cif. | Skin infection | No | Yes |
| *Persea americana* Mill. | Passive sexual interest | No | Yes |
| *Phytolacca dodecandra* L'Hér. | Amoeba | No | Yes |
| *Phytolacca dodecandra* L'Hér. | Giardia | No | Yes |
| *Phytolacca dodecandra* L'Hér. | Gonorrhea | No | Yes |
| *Phytolacca dodecandra* L'Hér. | Swellings | No | Yes |
| *Pittosporum abyssinicum* Delile | Tuberculosis | No | Yes |
| *Coleus igniarius* Schweinf. | Amoeba | No | Yes |
| *Coleus igniarius* Schweinf. | Evil eye | No | Yes |
| *Coleus igniarius* Schweinf. | Febrile illness | No | Yes |
| *Coleus igniarius* Schweinf. | Skin infection | No | Yes |
| *Coleus igniarius* Schweinf. | Spiritual | No | Yes |
| *Coleus igniarius* Schweinf. | Wound | No | Yes |
| *Afrocarpus falcatus* (Thunb.) C.N.Page | Gonorrhea | No | Yes |
| *Premna schimperi* Engl. | Febrile illness | No | Yes |
| *Premna schimperi* Engl. | Lung infection | No | Yes |
| *Psidium guajava* L. | Diabetes | No | Yes |
| *Psidium guajava* L. | Malaria | No | Yes |
| *Psidium guajava* L. | Stomachache | No | Yes |
| *Rhamnus prinoides* L'Hér. | Gonorrhea | No | Yes |
| *Rhamnus prinoides* L'Hér. | Skin infection | No | Yes |
| *Rhamnus prinoides* L'Hér. | Stomachache | No | Yes |
| *Searsia glutinosa* (Hochst. ex A.Rich.) Moffett | Lung infection | No | Yes |
| *Searsia glutinosa* (Hochst. ex A.Rich.) Moffett | Spiritual | No | Yes |
| *Ricinus communis* L. | Jaundice | No | Yes |
| *Ricinus communis* L. | Tonsillitis | No | Yes |
| *Ricinus communis* L. | Wound | No | Yes |
| *Rotheca myricoides* (Hochst.) Steane & Mabb. | Amoeba | No | Yes |
| *Rotheca myricoides* (Hochst.) Steane & Mabb. | Cancer | No | Yes |
| *Rotheca myricoides* (Hochst.) Steane & Mabb. | Diarrhea | No | Yes |
| *Rotheca myricoides* (Hochst.) Steane & Mabb. | Glandular | No | Yes |
| *Rotheca myricoides* (Hochst.) Steane & Mabb. | Jaundice | No | Yes |
| *Rotheca myricoides* (Hochst.) Steane & Mabb. | Lung infection | No | Yes |
| *Rotheca myricoides* (Hochst.) Steane & Mabb. | Skin infection | No | Yes |
| *Rumex abyssinicus* Jacq. | Amoeba | No | Yes |
| *Rumex abyssinicus* Jacq. | Gastric diseases | No | Yes |
| *Rumex abyssinicus* Jacq. | Skin infection | No | Yes |
| *Rumex nepalensis* Spreng. | Stomachache | No | Yes |
| *Ruta chalepensis* L. | Diarrhea | No | Yes |
| *Ruta chalepensis* L. | Goiter | No | Yes |
| *Ruta chalepensis* L. | Skin infection | No | Yes |
| *Ruta chalepensis* L. | Spiritual | No | Yes |
| *Saccharum officinarum* L. | Gastric diseases | No | Yes |
| *Searsia natalensis* (Bernh. ex Krauss) F.A.Barkley | Snake poison | No | Yes |
| *Solanum incanum* L. | Spiritual | No | Yes |
| *Stephania abyssinica* (Quart.-Dill. & A.Rich.) Walp. | Glandular | No | Yes |
| *Syzygium guineense* (Willd.) DC. | Amoeba | No | Yes |
| *Syzygium guineense* (Willd.) DC. | Diarrhea | No | Yes |
| *Syzygium guineense* (Willd.) DC. | Muscle pain | No | Yes |
| *Syzygium guineense* (Willd.) DC. | Spiritual | No | Yes |
| *Taverniera abyssinica* A.Rich. | Febrile illness | No | Yes |
| *Taverniera abyssinica* A.Rich. | Headache | No | Yes |
| *Thymus schimperi* Ronniger | Blood pressure | No | Yes |
| *Thymus schimperi* Ronniger | Cholesterol | No | Yes |
| *Trigonella foenum-graecum* L. | Blood pressure | No | Yes |
| *Trigonella foenum-graecum* L. | Cholesterol | No | Yes |
| *Trigonella foenum-graecum* L. | Kidney infection | No | Yes |
| *Triticum turgidum subsp. dicoccum* (Schrank ex Schübl.) Thell. | Wound | No | Yes |
| *Urtica dioica* L. | Amoeba | No | Yes |
| *Urtica dioica* L. | Gonorrhea | No | Yes |
| *Urtica dioica* L. | Spiritual | No | Yes |
| *Urtica simensis* Hochst. ex A.Rich. | Fire accident | No | Yes |
| *Urtica simensis* Hochst. ex A.Rich. | Spiritual | No | Yes |
| *Gymnanthemum auriculiferum* (Hiern) Isawumi | Spiritual | No | Yes |
| *Vicia faba* L. | Gastric diseases | No | Yes |
| *Zingiber officinale* Roscoe | Asthma | No | Yes |
| *Zingiber officinale* Roscoe | Cough | No | Yes |
| *Zingiber officinale* Roscoe | Goiter | No | Yes |
| *Zingiber officinale* Roscoe | Headache | No | Yes |
| *Zingiber officinale* Roscoe | Malaria | No | Yes |
| *Zingiber officinale* Roscoe | Tung infection | No | Yes |
