# Supplementary material for: Ethnobotanical study on medicinal plant knowledge among three ethnic groups in peri-urban areas of south-central Ethiopia
Source: J Ethnobiol Ethnomed. 2023 Nov 23;19:55. doi: 10.1186/s13002-023-00629-w (PMC10668360; doi:10.1186/s13002-023-00629-w)
Supplement: Supplementary file 5 — Additional file 5. Evaluation of useful plants of the three studied ethnic groups, using four quantitative indices. [file 13002_2023_629_MOESM5_ESM.docx]

Supplementary Table 5. Evaluation of useful plants of the three studied ethnic groups in Southcentral Ethiopia, Using Four Quantitative Indices (Cultural importance, Relative frequency of citation, Cultural value, and Relative importance). List of the first 78 medicinal plants, each claimed by three or more informants as a remedy against a particular type of ailment, Following the CI Index and Plant Ranking based on Each Index and ethnic group. NB: CI=Cultural importance, RFC=Relative Frequency of citation, CV=Cultural value, and RI=Relative importance. Broken lines indicate the absence of a citation for the indicated species in the study area.

| SPECIES | GEDEO | | | | | | | | | | | OROMO | | | | | | | | | | | | SIDAMA | | | | | | | | | | | |
| --- | --- | --- | --- | --- | --- | --- | --- | --- | --- | --- | --- | --- | --- | --- | --- | --- | --- | --- | --- | --- | --- | --- | --- | --- | --- | --- | --- | --- | --- | --- | --- | --- | --- | --- | --- |
|  | BASIC VALUES | | | INDICES | | | | RANKS | | | | BASIC VALUES | | | | INDICES | | | | RANKS | | | | BASIC VALUES | | | INDICES | | | | | RANKS | | | |
|  | FC | UR | NU | CI | RFC | CV | RI | CI | RFC | RI | CV | FC | UR | NU | CI | | RFC | RI | CV | CI | RFC | RI | CV | FC | UR | NU | CI | RFC | RI | CV | CI | | RFC | RI | CV |
| *Vachellia oerfota (Forssk.) Kyal. & Boatwr.* | -- | -- | -- | -- | -- | -- | -- | -- | -- | -- | -- | 8 | 8 | 2 | 0.127 | | 0.127 | 0.236 | 0 | 23 | 15 | 40 | 27 | -- | -- | -- | -- | -- | -- | -- | -- | | -- | -- | -- |
| *Achyranthes aspera* L. | 4 | 6 | 5 | 0.095 | 0.063 | 0.19 | 0 | 31 | 30 | 32 | 31 | 7 | 7 | 5 | 0.111 | | 0.111 | 0.28 | 0.001 | 26 | 17 | 25 | 18 | 4 | 7 | 6 | 0.111 | 0.063 | 0.318 | 0.001 | 23 | | 34 | 18 | 23 |
| *Acokanthera schimperi* (A.DC.) Schweinf. | -- | -- | -- | -- | -- | -- | -- | -- | -- | -- | -- | -- | -- | -- | -- | | -- | -- | -- | -- | -- | -- | -- | 10 | 12 | 5 | 0.19 | 0.159 | 0.461 | 0.003 | 15 | | 9 | 14 | 13 |
| *Albizia gummifera* (J.F.Gmel.) C.A.Sm. | 20 | 47 | 20 | 0.746 | 0.317 | 0.833 | 0.073 | 2 | 2 | 2 | 2 | 6 | 10 | 7 | 0.159 | | 0.095 | 0.302 | 0.002 | 18 | 20 | 15 | 17 | 7 | 13 | 8 | 0.206 | 0.111 | 0.473 | 0.003 | 12 | | 18 | 12 | 12 |
| *Allium sativum* L. | 4 | 11 | 7 | 0.175 | 0.063 | 0.238 | 0.001 | 15 | 27 | 20 | 20 | 5 | 12 | 6 | 0.19 | | 0.079 | 0.255 | 0.001 | 14 | 26 | 22 | 25 | 8 | 23 | 9 | 0.365 | 0.127 | 0.535 | 0.007 | 4 | | 13 | 8 | 9 |
| *Aloe macrocarpa* Tod. | -- | -- | -- | -- | -- | -- | -- | -- | -- | -- | -- | 16 | 38 | 14 | 0.603 | | 0.254 | 0.699 | 0.033 | 2 | 3 | 3 | 3 | -- | -- | -- | -- | -- | -- | -- | -- | | -- | -- | -- |
| *Aloe vera* (L.) Burm.f. | -- | -- | -- | -- | -- | -- | -- | -- | -- | -- | -- | -- | -- | -- | -- | | -- | -- | -- | -- | -- | -- | -- | 5 | 6 | 4 | 0.095 | 0.079 | 0.28 | 0.001 | 29 | | 28 | 25 | 28 |
| *Argemone mexicana* L. | -- | -- | -- | -- | -- | -- | -- | -- | -- | -- | -- | 5 | 5 | 4 | 0.079 | | 0.079 | 0.21 | 0 | 40 | 34 | 35 | 36 | -- | -- | -- | -- | -- | -- | -- | -- | | -- | -- | -- |
| *Artemisia abyssinica* Sch.Bip. ex A.Rich. | 6 | 11 | 6 | 0.175 | 0.095 | 0.25 | 0.002 | 16 | 16 | 18 | 19 | -- | -- | -- | -- | | -- | -- | -- | -- | -- | -- | -- | -- | -- | -- | -- | -- | -- | -- | -- | | -- | -- | -- |
| *Asparagus africanus* Lam*.* | 10 | 13 | 7 | 0.206 | 0.159 | 0.345 | 0.004 | 11 | 9 | 9 | 10 | 6 | 6 | 5 | 0.095 | | 0.095 | 0.256 | 0.001 | 32 | 22 | 27 | 24 | -- | -- | -- | -- | -- | -- | -- | -- | | -- | -- | -- |
| *Balanites aegyptiaca* (L.) Delile | -- | -- | -- | -- | -- | -- | -- | -- | -- | -- | -- | -- | -- | -- | -- | | -- | -- | -- | -- | -- | -- | -- | 4 | 7 | 3 | 0.111 | 0.063 | 0.218 | 0 | 24 | | 35 | 33 | 37 |
| *Bersama abyssinica* Fresen. | 4 | 5 | 4 | 0.079 | 0.063 | 0.167 | 0 | 38 | 34 | 37 | 37 | -- | -- | -- | -- | | -- | -- | -- | -- | -- | -- | -- | -- | -- | -- | -- | -- | -- | -- | -- | | -- | -- | -- |
| *Calpurnia aurea* (Aiton) Benth. | 19 | 31 | 13 | 0.492 | 0.302 | 0.649 | 0.03 | 3 | 3 | 3 | 4 | 11 | 14 | 9 | 0.222 | | 0.175 | 0.466 | 0.005 | 10 | 8 | 9 | 9 | 4 | 5 | 5 | 0.079 | 0.063 | 0.284 | 0 | 36 | | 37 | 28 | 27 |
| *Carica papaya* L. | -- | -- | -- | -- | -- | -- | -- | -- | -- | -- | -- | 4 | 7 | 6 | 0.111 | | 0.063 | 0.232 | 0.001 | 27 | 37 | 24 | 33 | 7 | 10 | 4 | 0.159 | 0.111 | 0.339 | 0.001 | 16 | | 19 | 22 | 19 |
| *Carissa spinarum* L. | -- | -- | -- | -- | -- | -- | -- | -- | -- | -- | -- | 6 | 6 | 3 | 0.095 | | 0.095 | 0.211 | 0 | 33 | 23 | 37 | 34 | -- | -- | -- | -- | -- | -- | -- | -- | | -- | -- | -- |
| *Catha edulis* (Vahl) Forssk. ex Endl. | -- | -- | -- | -- | -- | -- | -- | -- | -- | -- | -- | -- | -- | -- | -- | | -- | -- | -- | -- | -- | -- | -- | 7 | 9 | 4 | 0.143 | 0.111 | 0.339 | 0.001 | 20 | | 20 | 23 | 20 |
| *Celtis africana* Burm.f. | 10 | 18 | 11 | 0.286 | 0.159 | 0.44 | 0.008 | 7 | 8 | 7 | 7 | -- | -- | -- | -- | | -- | -- | -- | -- | -- | -- | -- | -- | -- | -- | -- | -- | -- | -- | -- | | -- | -- | -- |
| *Cinnamomum verum* J.Presl | -- | -- | -- | -- | -- | -- | -- | -- | -- | -- | -- | -- | -- | -- | -- | | -- | -- | -- | -- | -- | -- | -- | 3 | 5 | 3 | 0.079 | 0.048 | 0.188 | 0 | 37 | | 39 | 34 | 38 |
| *Citrus limon* (L.) Osbeck | -- | -- | -- | -- | -- | -- | -- | -- | -- | -- | -- | 6 | 11 | 5 | 0.175 | | 0.095 | 0.256 | 0.001 | 16 | 19 | 26 | 23 | -- | -- | -- | -- | -- | -- | -- | -- | | -- | -- | -- |
| *Clutia abyssinica* Jaub. & Spach | -- | -- | -- | -- | -- | -- | -- | -- | -- | -- | -- | 6 | 7 | 6 | 0.111 | | 0.095 | 0.279 | 0.001 | 28 | 21 | 20 | 19 | 6 | 6 | 4 | 0.095 | 0.095 | 0.31 | 0.001 | 30 | | 24 | 24 | 25 |
| *Coffea arabica* L. | 9 | 12 | 8 | 0.19 | 0.143 | 0.351 | 0.003 | 12 | 10 | 12 | 9 | 5 | 9 | 5 | 0.143 | | 0.079 | 0.233 | 0.001 | 20 | 27 | 28 | 28 | -- | -- | -- | -- | -- | -- | -- | -- | | -- | -- | -- |
| *Croton macrostachyus* Hochst. ex Delile | 28 | 80 | 21 | 1.27 | 0.444 | 1 | 0.182 | 1 | 1 | 1 | 1 | 21 | 48 | 22 | 0.762 | | 0.333 | 1 | 0.086 | 1 | 1 | 1 | 1 | 17 | 36 | 15 | 0.571 | 0.27 | 1 | 0.039 | 2 | | 1 | 1 | 1 |
| *Cucumis prophetarum* L. | -- | -- | -- | -- | -- | -- | -- | -- | -- | -- | -- | -- | -- | -- | -- | | -- | -- | -- | -- | -- | -- | -- | 14 | 20 | 9 | 0.317 | 0.222 | 0.712 | 0.011 | 6 | | 4 | 3 | 3 |
| *Cymbopogon citratus* (DC.) Stapf | 5 | 15 | 9 | 0.238 | 0.079 | 0.304 | 0.003 | 9 | 21 | 11 | 13 | -- | -- | -- | -- | | -- | -- | -- | -- | -- | -- | -- | -- | -- | -- | -- | -- | -- | -- | -- | | -- | -- | -- |
| *Datura stramonium* L. test  . | -- | -- | -- | -- | -- | -- | -- | -- | -- | -- | -- | 6 | 6 | 3 | 0.095 | | 0.095 | 0.211 | 0 | 34 | 24 | 38 | 35 | -- | -- | -- | -- | -- | -- | -- | -- | | -- | -- | -- |
| *Dodonaea viscosa subsp. angustifolia* (L.f.) J.G.West | -- | -- | -- | -- | -- | -- | -- | -- | -- | -- | -- | 8 | 9 | 6 | 0.143 | | 0.127 | 0.327 | 0.002 | 21 | 14 | 16 | 15 | -- | -- | -- | -- | -- | -- | -- | -- | | -- | -- | -- |
| *Drynaria volkensii* Heiron. | 4 | 6 | 4 | 0.095 | 0.063 | 0.167 | 0 | 32 | 31 | 36 | 36 | -- | -- | -- | -- | | -- | -- | -- | -- | -- | -- | -- | -- | -- | -- | -- | -- | -- | -- | -- | | -- | -- | -- |
| *Echinops kebericho* Mesfin | -- | -- | -- | -- | -- | -- | -- | -- | -- | -- | -- | -- | -- | -- | -- | | -- | -- | -- | -- | -- | -- | -- | 4 | 10 | 4 | 0.159 | 0.063 | 0.251 | 0.001 | 17 | | 32 | 26 | 31 |
| *Ehretia cymosa* Thonn. | -- | -- | -- | -- | -- | -- | -- | -- | -- | -- | -- | -- | -- | -- | -- | | -- | -- | -- | -- | -- | -- | -- | 5 | 5 | 5 | 0.079 | 0.079 | 0.314 | 0.001 | 38 | | 30 | 20 | 24 |
| *Ekebergia capensis* Sparrm. | 5 | 9 | 5 | 0.143 | 0.079 | 0.208 | 0.001 | 19 | 22 | 24 | 25 | 6 | 14 | 10 | 0.222 | | 0.095 | 0.37 | 0.003 | 11 | 18 | 12 | 12 | 15 | 20 | 8 | 0.317 | 0.238 | 0.708 | 0.01 | 7 | | 2 | 4 | 4 |
| *Ensete ventricosum* (Welw.) Cheesman | 6 | 7 | 4 | 0.111 | 0.095 | 0.202 | 0.001 | 27 | 19 | 28 | 27 | -- | -- | -- | -- | | -- | -- | -- | -- | -- | -- | -- | -- | -- | -- | -- | -- | -- | -- | -- | | -- | -- | -- |
| *Erythrina abyssinica* Lam. | 6 | 10 | 8 | 0.159 | 0.095 | 0.298 | 0.002 | 18 | 17 | 14 | 15 | 6 | 6 | 6 | 0.095 | | 0.095 | 0.279 | 0.001 | 35 | 25 | 21 | 20 | -- | -- | -- | -- | -- | -- | -- | -- | | -- | -- | -- |
| *Eucalyptus globulus* Labill. | 5 | 7 | 5 | 0.111 | 0.079 | 0.208 | 0.001 | 28 | 25 | 25 | 26 | 9 | 16 | 5 | 0.254 | | 0.143 | 0.328 | 0.003 | 7 | 10 | 14 | 13 | 9 | 20 | 11 | 0.317 | 0.143 | 0.631 | 0.008 | 8 | | 11 | 6 | 6 |
| *Galinsoga quadriradiata* Ruiz & Pav. | 7 | 9 | 4 | 0.143 | 0.111 | 0.22 | 0.001 | 20 | 13 | 27 | 23 | -- | -- | -- | -- | | -- | -- | -- | -- | -- | -- | -- | -- | -- | -- | -- | -- | -- | -- | -- | | -- | -- | -- |
| *Grewia ferruginea* Hochst. ex A.Rich. | 5 | 9 | 8 | 0.143 | 0.079 | 0.28 | 0.001 | 21 | 23 | 19 | 17 | -- | -- | -- | -- | | -- | -- | -- | -- | -- | -- | -- | -- | -- | -- | -- | -- | -- | -- | -- | | -- | -- | -- |
| *Hagenia abyssinica* (Bruce) J.F.Gmel. | -- | -- | -- | -- | -- | -- | -- | -- | -- | -- | -- | 5 | 7 | 5 | 0.111 | | 0.079 | 0.233 | 0.001 | 29 | 30 | 30 | 30 | -- | -- | -- | -- | -- | -- | -- | -- | | -- | -- | -- |
| *Justicia schimperiana* (Hochst. ex Nees) T.Anderson | 6 | 8 | 5 | 0.127 | 0.095 | 0.226 | 0.001 | 26 | 18 | 23 | 21 | 9 | 11 | 4 | 0.175 | | 0.143 | 0.305 | 0.002 | 17 | 11 | 17 | 16 | 8 | 9 | 5 | 0.143 | 0.127 | 0.402 | 0.002 | 21 | | 17 | 15 | 17 |
| *Kalanchoe petitiana* A.Rich. | -- | -- | -- | -- | -- | -- | -- | -- | -- | -- | -- | -- | -- | -- | -- | | -- | -- | -- | -- | -- | -- | -- | 6 | 7 | 5 | 0.111 | 0.095 | 0.343 | 0.001 | 25 | | 23 | 19 | 18 |
| *Lactuca inermis* Forssk. | 4 | 9 | 3 | 0.143 | 0.063 | 0.143 | 0 | 22 | 28 | 39 | 39 | -- | -- | -- | -- | | -- | -- | -- | -- | -- | -- | -- | -- | -- | -- | -- | -- | -- | -- | -- | | -- | -- | -- |
| *Lagenaria siceraria* (Molina) Standl. | 3 | 5 | 4 | 0.079 | 0.048 | 0.149 | 0 | 39 | 39 | 38 | 38 | -- | -- | -- | -- | | -- | -- | -- | -- | -- | -- | -- | 5 | 6 | 3 | 0.095 | 0.079 | 0.247 | 0 | 31 | | 29 | 32 | 33 |
| *Linum usitatissimum* L. | -- | -- | -- | -- | -- | -- | -- | -- | -- | -- | -- | -- | -- | -- | -- | | -- | -- | -- | -- | -- | -- | -- | 4 | 8 | 5 | 0.127 | 0.063 | 0.284 | 0.001 | 22 | | 33 | 21 | 26 |
| *Maesa lanceolata* Forssk. | 7 | 9 | 7 | 0.143 | 0.111 | 0.292 | 0.002 | 23 | 14 | 15 | 16 | -- | -- | -- | -- | | -- | -- | -- | -- | -- | -- | -- | -- | -- | -- | -- | -- | -- | -- | -- | | -- | -- | -- |
| *Melia azedarach* L. | 3 | 6 | 6 | 0.095 | 0.048 | 0.196 | 0 | 33 | 37 | 30 | 28 | 11 | 16 | 11 | 0.254 | | 0.175 | 0.512 | 0.008 | 8 | 7 | 7 | 6 | 8 | 15 | 9 | 0.238 | 0.127 | 0.535 | 0.005 | 10 | | 14 | 9 | 10 |
| *Millettia ferruginea* (Hochst.) Hochst. ex Baker | 6 | 12 | 10 | 0.19 | 0.095 | 0.345 | 0.003 | 13 | 15 | 10 | 11 | -- | -- | -- | -- | | -- | -- | -- | -- | -- | -- | -- | -- | -- | -- | -- | -- | -- | -- | -- | | -- | -- | -- |
| *Momordica boivinii* Baill. | -- | -- | -- | -- | -- | -- | -- | -- | -- | -- | -- | -- | -- | -- | -- | | -- | -- | -- | -- | -- | -- | -- | 7 | 7 | 6 | 0.111 | 0.111 | 0.406 | 0.001 | 26 | | 21 | 17 | 16 |
| *Moringa stenopetala* (Baker f.) Cufod. | 4 | 9 | 5 | 0.143 | 0.063 | 0.19 | 0.001 | 24 | 29 | 26 | 30 | 13 | 25 | 14 | 0.397 | | 0.206 | 0.628 | 0.018 | 4 | 4 | 5 | 5 | 3 | 5 | 2 | 0.079 | 0.048 | 0.155 | 0 | 39 | | 40 | 37 | 40 |
| *Nigella sativa* L. | 3 | 7 | 7 | 0.111 | 0.048 | 0.22 | 0.001 | 29 | 35 | 21 | 22 | 7 | 12 | 10 | 0.19 | | 0.111 | 0.394 | 0.003 | 15 | 16 | 11 | 11 | -- | -- | -- | -- | -- | -- | -- | -- | | -- | -- | -- |
| *Ocimum jamesii* Sebald | -- | -- | -- | -- | -- | -- | -- | -- | -- | -- | -- | -- | -- | -- | -- | | -- | -- | -- | -- | -- | -- | -- | 5 | 5 | 1 | 0.079 | 0.079 | 0.18 | 0 | 40 | | 31 | 40 | 39 |
| *Ocimum lamiifolium* Hochst. ex Benth. | 3 | 6 | 6 | 0.095 | 0.048 | 0.196 | 0 | 34 | 38 | 31 | 29 | -- | -- | -- | -- | | -- | -- | -- | -- | -- | -- | -- | -- | -- | -- | -- | -- | -- | -- | -- | | -- | -- | -- |
| *Ocimum gratissimum* L. | 7 | 15 | 6 | 0.238 | 0.111 | 0.268 | 0.002 | 10 | 12 | 17 | 18 | 8 | 14 | 6 | 0.222 | | 0.127 | 0.327 | 0.003 | 12 | 13 | 13 | 14 | -- | -- | -- | -- | -- | -- | -- | -- | | -- | -- | -- |
| *Olea europaea subsp. cuspidata* (Wall. & G.Don) Cif. | 9 | 11 | 6 | 0.175 | 0.143 | 0.304 | 0.002 | 17 | 11 | 16 | 14 | 11 | 25 | 18 | 0.397 | | 0.175 | 0.671 | 0.019 | 5 | 6 | 4 | 4 | -- | -- | -- | -- | -- | -- | -- | -- | | -- | -- | -- |
| *Olinia rochetiana* A.Juss. | -- | -- | -- | -- | -- | -- | -- | -- | -- | -- | -- | 12 | 13 | 8 | 0.206 | | 0.19 | 0.468 | 0.005 | 13 | 5 | 10 | 8 | -- | -- | -- | -- | -- | -- | -- | -- | | -- | -- | -- |
| *Phytolacca dodecandra* L'Hér. | 3 | 5 | 5 | 0.079 | 0.048 | 0.173 | 0 | 40 | 40 | 35 | 35 | -- | -- | -- | -- | | -- | -- | -- | -- | -- | -- | -- | 9 | 14 | 7 | 0.222 | 0.143 | 0.498 | 0.004 | 11 | | 12 | 11 | 11 |
| *Coleus igniarius* Schweinf. | -- | -- | -- | -- | -- | -- | -- | -- | -- | -- | -- | -- | -- | -- | -- | | -- | -- | -- | -- | -- | -- | -- | 8 | 13 | 6 | 0.206 | 0.127 | 0.435 | 0.003 | 13 | | 15 | 13 | 15 |
| *Afrocarpus falcatus* (Thunb.) C.N.Page | 16 | 26 | 10 | 0.413 | 0.254 | 0.524 | 0.016 | 5 | 4 | 5 | 5 | -- | -- | -- | -- | | -- | -- | -- | -- | -- | -- | -- | -- | -- | -- | -- | -- | -- | -- | -- | | -- | -- | -- |
| *Psidium guajava* L. | -- | -- | -- | -- | -- | -- | -- | -- | -- | -- | -- | 3 | 6 | 4 | 0.095 | | 0.048 | 0.162 | 0 | 36 | 40 | 36 | 40 | -- | -- | -- | -- | -- | -- | -- | -- | | -- | -- | -- |
| *Psydrax schimperianus* (A.Rich.) Bridson | -- | -- | -- | -- | -- | -- | -- | -- | -- | -- | -- | 5 | 7 | 5 | 0.111 | | 0.079 | 0.233 | 0.001 | 30 | 31 | 31 | 31 | -- | -- | -- | -- | -- | -- | -- | -- | | -- | -- | -- |
| *Searsia glutinosa* (Hochst. ex A.Rich.) Moffett | -- | -- | -- | -- | -- | -- | -- | -- | -- | -- | -- | -- | -- | -- | -- | | -- | -- | -- | -- | -- | -- | -- | 12 | 13 | 3 | 0.206 | 0.19 | 0.453 | 0.002 | 14 | | 7 | 16 | 14 |
| *Ricinus communis* L. | 5 | 9 | 4 | 0.143 | 0.079 | 0.185 | 0.001 | 25 | 24 | 29 | 33 | -- | -- | -- | -- | | -- | -- | -- | -- | -- | -- | -- | 3 | 7 | 5 | 0.111 | 0.048 | 0.255 | 0 | 27 | | 38 | 29 | 30 |
| *Rotheca myricoides* (Hochst.) Steane & Mabb. | -- | -- | -- | -- | -- | -- | -- | -- | -- | -- | -- | -- | -- | -- | -- | | -- | -- | -- | -- | -- | -- | -- | 11 | 16 | 7 | 0.254 | 0.175 | 0.557 | 0.005 | 9 | | 8 | 10 | 8 |
| *Rubus steudneri* Schweinf. | -- | -- | -- | -- | -- | -- | -- | -- | -- | -- | -- | 4 | 8 | 8 | 0.127 | | 0.063 | 0.277 | 0.001 | 24 | 36 | 19 | 22 | -- | -- | -- | -- | -- | -- | -- | -- | | -- | -- | -- |
| *Ruta chalepensis* L. | 15 | 28 | 17 | 0.444 | 0.238 | 0.673 | 0.028 | 4 | 5 | 4 | 3 | 9 | 19 | 13 | 0.302 | | 0.143 | 0.51 | 0.009 | 6 | 9 | 6 | 7 | 14 | 21 | 8 | 0.333 | 0.222 | 0.678 | 0.01 | 5 | | 3 | 5 | 5 |
| *Schinus molle* L. | -- | -- | -- | -- | -- | -- | -- | -- | -- | -- | -- | 4 | 6 | 3 | 0.095 | | 0.063 | 0.163 | 0 | 37 | 38 | 39 | 39 | -- | -- | -- | -- | -- | -- | -- | -- | | -- | -- | -- |
| *Searsia natalensis* (Bernh. ex Krauss) F.A.Barkley | -- | -- | -- | -- | -- | -- | -- | -- | -- | -- | -- | -- | -- | -- | -- | | -- | -- | -- | -- | -- | -- | -- | 7 | 7 | 1 | 0.111 | 0.111 | 0.239 | 0 | 28 | | 22 | 39 | 36 |
| *Sida schimperiana* Hochst. ex A.Rich. | 4 | 6 | 6 | 0.095 | 0.063 | 0.214 | 0.001 | 35 | 32 | 22 | 24 | -- | -- | -- | -- | | -- | -- | -- | -- | -- | -- | -- | -- | -- | -- | -- | -- | -- | -- | -- | | -- | -- | -- |
| *Solanecio gigas* (Vatke) C.Jeffrey | 13 | 20 | 10 | 0.317 | 0.206 | 0.47 | 0.01 | 6 | 6 | 6 | 6 | -- | -- | -- | -- | | -- | -- | -- | -- | -- | -- | -- | -- | -- | -- | -- | -- | -- | -- | -- | | -- | -- | -- |
| *Solanum incanum* L. | -- | -- | -- | -- | -- | -- | -- | -- | -- | -- | -- | 5 | 9 | 3 | 0.143 | | 0.079 | 0.187 | 0.001 | 22 | 28 | 33 | 38 | 6 | 6 | 3 | 0.095 | 0.095 | 0.276 | 0 | 32 | | 25 | 31 | 29 |
| *Solanum marginatum* L.f. | -- | -- | -- | -- | -- | -- | -- | -- | -- | -- | -- | 5 | 7 | 6 | 0.111 | | 0.079 | 0.255 | 0.001 | 31 | 32 | 23 | 26 | -- | -- | -- | -- | -- | -- | -- | -- | | -- | -- | -- |
| *Stephania abyssinica* (Quart.-Dill. & A.Rich.) Walp. | -- | -- | -- | -- | -- | -- | -- | -- | -- | -- | -- | -- | -- | -- | -- | | -- | -- | -- | -- | -- | -- | -- | 6 | 6 | 2 | 0.095 | 0.095 | 0.243 | 0 | 33 | | 26 | 35 | 34 |
| *Syzygium guineense* (Willd.) DC. | 4 | 6 | 5 | 0.095 | 0.063 | 0.19 | 0 | 36 | 33 | 33 | 32 | 5 | 6 | 5 | 0.095 | | 0.079 | 0.233 | 0.001 | 38 | 33 | 32 | 32 | 4 | 6 | 4 | 0.095 | 0.063 | 0.251 | 0 | 34 | | 36 | 30 | 32 |
| *Vepris nobilis* (Delile) Mziray | -- | -- | -- | -- | -- | -- | -- | -- | -- | -- | -- | 4 | 6 | 5 | 0.095 | | 0.063 | 0.209 | 0 | 39 | 39 | 34 | 37 | -- | -- | -- | -- | -- | -- | -- | -- | | -- | -- | -- |
| *Trigonella foenum-graecum* L. | 4 | 12 | 10 | 0.19 | 0.063 | 0.31 | 0.002 | 14 | 26 | 13 | 12 | -- | -- | -- | -- | | -- | -- | -- | -- | -- | -- | -- | -- | -- | -- | -- | -- | -- | -- | -- | | -- | -- | -- |
| *Urtica simensis* Hochst. ex A.Rich. | -- | -- | -- | -- | -- | -- | -- | -- | -- | -- | -- | -- | -- | -- | -- | | -- | -- | -- | -- | -- | -- | -- | 6 | 6 | 2 | 0.095 | 0.095 | 0.243 | 0 | 35 | | 27 | 36 | 35 |
| *Gymnanthemum amygdalinum* (Delile) Sch.Bip. | 11 | 17 | 8 | 0.27 | 0.175 | 0.387 | 0.006 | 8 | 7 | 8 | 8 | 21 | 34 | 15 | 0.54 | | 0.333 | 0.841 | 0.042 | 3 | 2 | 2 | 2 | 13 | 25 | 6 | 0.397 | 0.206 | 0.582 | 0.008 | 3 | | 5 | 7 | 7 |
| *Vicia faba* L. | 6 | 6 | 1 | 0.095 | 0.095 | 0.131 | 0 | 37 | 20 | 40 | 40 | -- | -- | -- | -- | | -- | -- | -- | -- | -- | -- | -- | 10 | 10 | 1 | 0.159 | 0.159 | 0.327 | 0 | 18 | | 10 | 38 | 22 |
| *Withania somnifera* (L.) Dunal | -- | -- | -- | -- | -- | -- | -- | -- | -- | -- | -- | 5 | 8 | 5 | 0.127 | | 0.079 | 0.233 | 0.001 | 25 | 29 | 29 | 29 | 8 | 10 | 3 | 0.159 | 0.127 | 0.335 | 0.001 | 19 | | 16 | 27 | 21 |
| *Zingiber officinale* Roscoe | 3 | 7 | 5 | 0.111 | 0.048 | 0.173 | 0 | 30 | 36 | 34 | 34 | 8 | 16 | 11 | 0.254 | | 0.127 | 0.44 | 0.005 | 9 | 12 | 8 | 10 | 12 | 46 | 13 | 0.73 | 0.19 | 0.786 | 0.03 | 1 | | 6 | 2 | 2 |
| *Ziziphus spina-christi* (L.) Willd. | -- | -- | -- | -- | -- | -- | -- | -- | -- | -- | -- | 4 | 10 | 8 | 0.159 | | 0.063 | 0.277 | 0.001 | 19 | 35 | 18 | 21 | -- | -- | -- | -- | -- | -- | -- | -- | | -- | -- | -- |
